# Hierarchical settlement behaviours of coral larvae to common coralline algae

**DOI:** 10.1038/s41598-023-32676-4

**Published:** 2023-04-09

**Authors:** M. A. Abdul Wahab, S. Ferguson, V. K. Snekkevik, G. McCutchan, S. Jeong, A. Severati, C. J. Randall, A. P. Negri, G. Diaz-Pulido

**Affiliations:** 1 Australian Institute of Marine Science, PMB No.3, Townsville, QLD 4810 Australia; 2 School of Environment and Science, Coastal and Marine Research Centre and Australian Rivers Institute, Griffith University, Brisbane, QLD 4111 Australia

**Keywords:** Behavioural ecology, Restoration ecology, Marine biology

## Abstract

Natural regeneration of degraded reefs relies on the recruitment of larvae to restore populations. Intervention strategies are being developed to enhance this process through aquaculture production of coral larvae and their deployment as spat. Larval settlement relies on cues associated with crustose coralline algae (CCA) that are known to induce attachment and metamorphosis. To understand processes underpinning recruitment, we tested larval settlement responses of 15 coral species, to 15 species of CCA from the Great Barrier Reef (GBR). CCA in the family Lithophyllaceae were overall the best inducer across most coral species, with *Titanoderma* cf. *tessellatum* being the most effective species that induced at least 50% settlement in 14 of the coral species (mean 81%). Taxonomic level associations were found, with species of *Porolithon* inducing high settlement in the genus *Acropora*; while a previously understudied CCA, *Sporolithon* sp., was a strong inducer for the Lobophyllidae. Habitat-specific associations were detected, with CCA collected from similar light environment as the coral inducing higher levels of settlement. This study revealed the intimate relationships between coral larvae and CCA and provides optimal coral-algal species pairings that could be utilized to increase the success of larval settlement to generate healthy spat for reef restoration.

## Introduction

For sessile marine invertebrates, the generation of motile planktonic reproductive propagules, such as larvae, is critical for range expansion and the genetic mixing and maintenance of populations^1–3^. Larvae may remain in the water column for periods ranging from minutes^4^ to months^5^, with longer durations aiding dispersal from natal habitats. Upon reaching competency for metamorphosis, larvae migrate to the benthos, where they explore and sense the substate in search of a suitable habitat^6^. The process of habitat selection can be complex, with several factors including substrate orientation, microtopography, and biochemical cues (reviewed in^7^) reported to induce larvae to attach and metamorphose, a process commonly termed ‘settlement’. The habitat where larvae choose to settle can have significant repercussions on post-settlement survival and growth^8,9^.

The larvae of scleractinian corals may encounter a variety of suitable habitats for settlement on tropical reefs. An optimal habitat for settlement would promote the survival of spat (early single polyp recruits) through consolidation (e.g. reef matrix that won’t easily be dislodged^10,11^), protection from grazing, predation and sediment smothering^12,13^, and adequate irradiance, requirements which could differ amongst coral species^14^. Importantly, substrata that have developed a microbial biofilm community can often induce metamorphosis^15^. In addition to microbial biofilms, colonisers such as coralline algae (phylum Rhodophyta, subclass Corallinophycidae) have been demonstrated as important biochemical inducers for coral larval settlement^16,17^. The potential for coralline algae to facilitate the recovery of coral populations, highlights their importance for the resilience of coral reefs, which are in a state of global decline.

Coralline red algae are a group of calcifying macroalgae that deposit primarily high-magnesium calcite in their cell walls. Coralline algae include two major functional groups: the non-geniculate crustose coralline algae (CCA), and the geniculate, articulated coralline algae, both of which are comprised of > 750 described species^18^. CCA are distributed worldwide from the tropics to polar regions, and from the intertidal to depths of > 260 m. On tropical coral reefs, in addition to their role in reef resilience, they are important for reef cementation and accretion. However, our understanding of the role of CCA in inducing settlement in coral larvae is limited to a handful of species, across both the algal and coral counterparts. Research in the Caribbean and Pacific on well-studied coral species in the families Agariicidae and Acroporidae has shown that larvae exhibit selectivity and hierarchical settlement responses to different species of CCA, with *Titanoderma* spp. (family Lithophylloideae) typically settlement across a range of coral species from both regions^8,19–22^. Other coralline algal genera that have been investigated previously for their inductive potential include *Hydrolithon*, *Porolithon*, *Amphiroa*, *Lithophorella*, and *Neogoniolithon,* with varying degrees of success*.* For example, acroporid larvae have been shown to avoid settlement to *Neogoniolithon fosliei,* possibly as a result of settlement inhibition strategies deployed by the CCA (e.g. epithallus shedding, overgrowth and chemical deterrents^8^). Clearly, these intimate CCA-coral associations could have direct implications for the survival of early-life stages and, consequently, species distributions. However, whether the inductive or inhibitory properties of these CCA are relevant beyond the Agariicidae and Acroporidae, and if larval settlement responses across CCA species are consistent within, and between, coral families is currently unknown.

**Figure 1:**
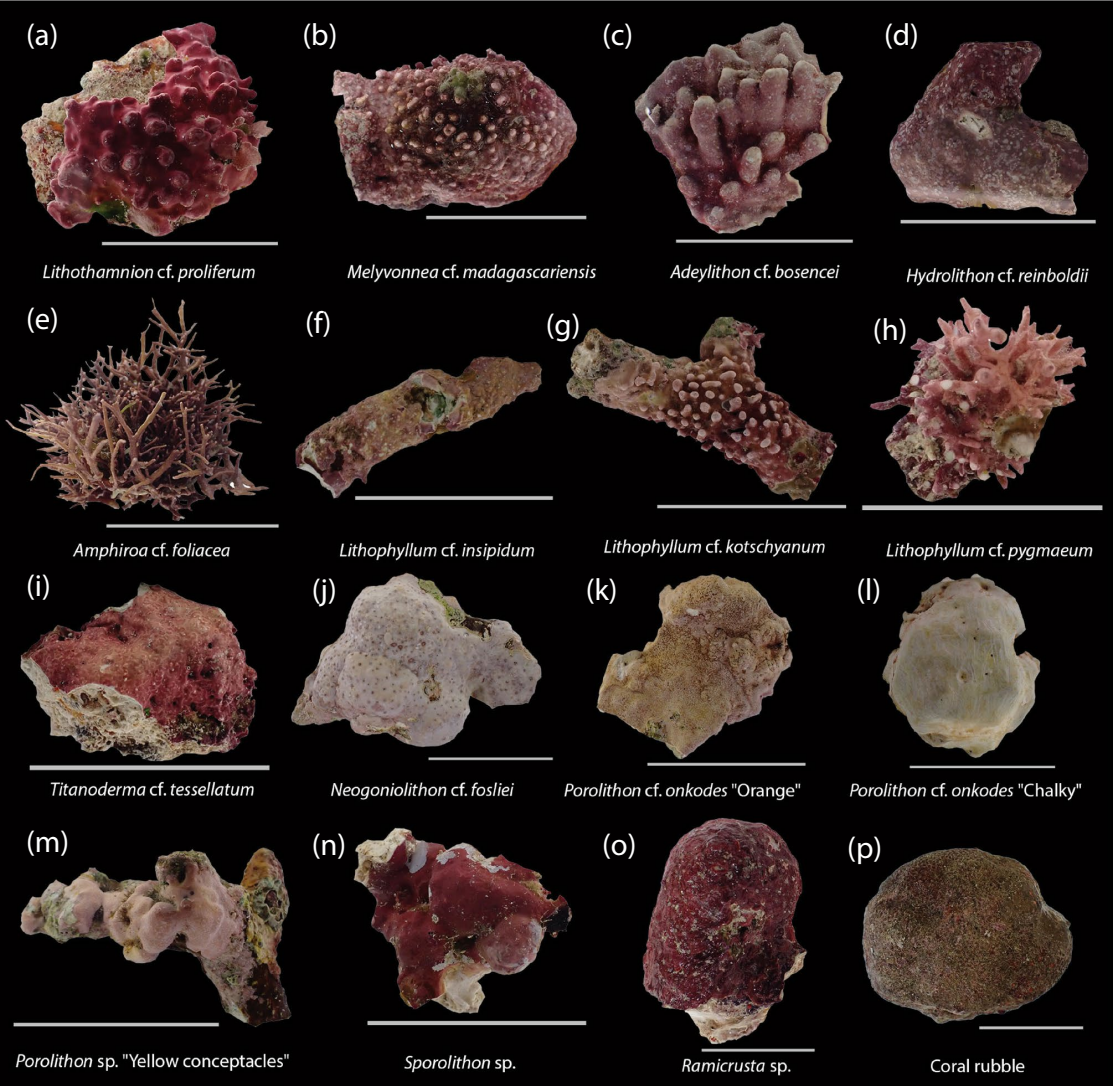
(**a**‒**n**) Sample fragments of coralline red algae (including crustose coralline algae and articulated, geniculate coralline algae), (**o**) *Ramicrusta* sp. (Peyssonneliaceae) and (**p**) coral rubble collected from the Great Barrier Reef and tested in the study. Scale bar = 5 cm.

To better understand the role of CCA in coral larval settlement more broadly, we tested settlement responses of larvae across 15 Great Barrier Reef (GBR) coral species from 5 taxonomic families that included the Acroporidae, Merulinidae, Lobophyllidae, Poritidae and Fungiidae from two consecutive spawning periods in October and November 2021 (Table 1), to 14 species of CCA from 7 taxonomic subfamilies/families that included the Lithophylloideae, Hydrolithoideae, Metagoniolithoideae, Mesophyllumaceae, Hapalidiaceae and Sporolithaceae, and a non-coralline species of crustose red calcifying alga, *Ramicrusta* sp. (family Peyssonneliaceae) (Fig. 1); collectively termed CCA hereafter for ease of communication. Here, we aimed to elucidate species-specific, intra-generic, and familial patterns of coral settlement preferences to common CCA species. Additionally, we assessed whether the origin and habitat from which CCA species were collected had an influence on coral larval settlement. Using a broad range of coral and coralline algal taxonomic groups, our study provides a comprehensive platform for further investigations into the intimate interaction between coral larval settlement and coralline algae and provides fundamental information on coral-algal species pairings useful to optimise larval settlement in aquaculture for reef restoration.

**Table 1:** Collection, modes of reproduction, spawning details, and the age of larvae at the start of the experiment for the 15 coral species used in the study.

Family	Coral species	Mode of reproduction	Gamete release type	Collection location	Number of colonies	Spawning date	Spawning time	Larval age (days)
Acroporidae	*Acropora tenuis* (October)	Hermaphroditic	Egg and sperm bundles; gentle	Palm and Magnetic Islands	13	24th October 2021	18:00–19:05 h	6
	*Acropora tenuis* (November)	Hermaphroditic	Egg and sperm bundles; gentle	Palm Islands	6	24th November 2021	18:35 h	7
	*Acropora anthocercis*	Hermaphroditic	Egg and sperm bundles; gentle	Magnetic Island	7	20th October 2021	21:16–22:30 h	6
	*Acropora hyacinthus*	Hermaphroditic	Egg and sperm bundles; gentle	Davies Reef	14	29th November 2021	21:45–23:37 h	7
	*Montipora aequituberculata*	Hermaphroditic	Egg and sperm bundles; gentle	Palm Islands	4	25th October 2021	19:38–20:50 h	6
Merulinidae	*Coeloastrea aspera*	Hermaphroditic	Egg and sperm bundles; gentle	Magnetic Island	14	24th October 2021	21:20–21:34 h	5, 8, 18
	*Caulastrea furcata*	Hermaphroditic	Egg and sperm bundles; gentle	Palm Islands	3	23rd November 2021	19:50–20:39 h	6
	*Dipsastrea favus*	Hermaphroditic	Egg and sperm bundles; vigorous	Magnetic Island	4	23rd October 2021	19:45–20:07 h	4
	*Goniastrea favulus*	Hermaphroditic	Eggs and sperm separately; passive	Magnetic Island	6	24th October 2021	19:20 h	8
	*Mycedium elephantotus*	Hermaphroditic	Egg and sperm bundles; gentle	Davies Reef	5	24th November 2021	20:54 h	8
	*Platygyra sinensis*	Hermaphroditic	Egg and sperm bundles; gentle	Magnetic Island	2	24th October 2021	19:34–20:04 h	4
	*Platygyra daedalea*	Hermaphroditic	Egg and sperm bundles; gentle	Palm Islands	6	23rd November 2021	18:50 h	6
Lobophyllidae	*Echinophyllia aspera*	Hermaphroditic	Egg and sperm bundles; gentle	Magnetic Island	4	28th October 2021	20:15–20:30 h	5
	*Lobophyllia corymbosa*	Hermaphroditic	Eggs and sperm separately; gentle	Palm Islands	6	26th November 2021	19:27 h	8
Poritidae	*Porites lobata*	Gonochoric	Eggs and sperm separately; vigorous	Palm Islands	1 male and 4 females	25th November 2021	21:15–2131 h	6
Fungiidae	*Fungia fungites*	Gonochoric	Eggs and sperm separately; vigorous	SeaSim captive	4 males and 2 females	25th November 2021	18:37–19:26 h	8

The full moon was on the 21st of October (00:56 h) and 19th of November 2021 (18:57 h) during the respective spawning periods.

This study aimed to test the hypotheses that (1) there are taxonomic associations between CCA and coral species for larval settlement, and (2) CCA species that occur in similar habitats to their coral counterpart would induce higher levels of settlement.

## Results

### Taxonomic identification and species delineation of CCA

**Figure 2:**
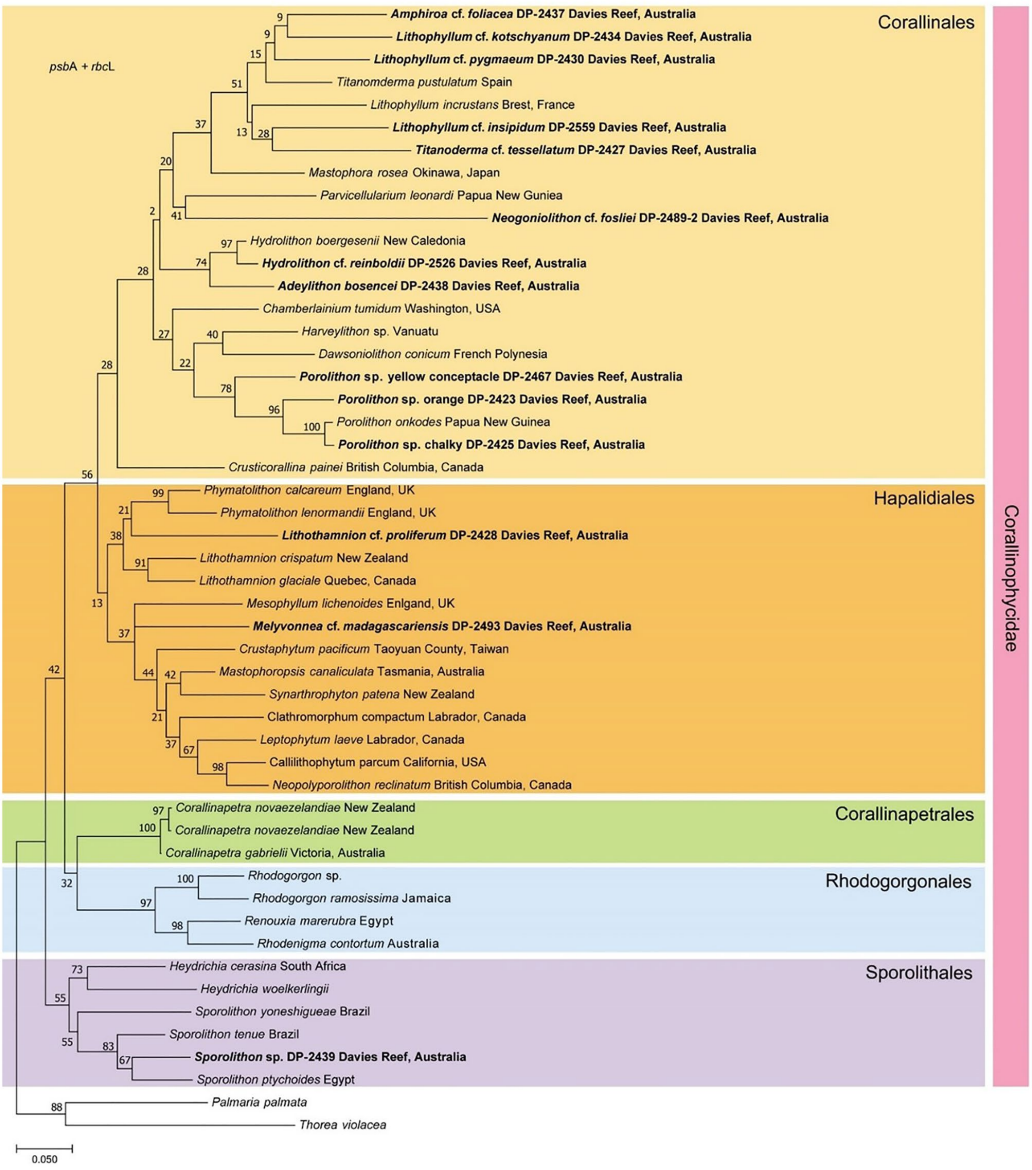
Phylogenetic tree based on Maximum Likelihood (ML) analysis of concatenated *psb*A and *rbc*L alignment. Values above branches denote maximum likelihood bootstrap values (BS) in %. Sequences obtained from this study are in bold.

A total of 30 DNA sequences were generated from representative specimens of each of the species used in the experiment (recently published in^23^), including 15 for *psb*A (368‒972 bp) and 15 for *rbc*L (349‒758 bp). The concatenated dataset comprised of 51 taxa with sequences ranging from 654 to 1173 nucleotides (Table 2, Fig. 2). Phylogenetic trees inferred from the *psb*A and *rbc*L sequences showed similar topologies in maximum-likelihood analyses to one another and to the concatenated phylogeny (Fig. 2, Supplementary Material 1). In total, eleven sequences were identified in the order Corallinales, two in the order Hapalidiales, and one in the order Sporolithales (Fig. 2). Identifications of majority of the studied genera were supported by *psbA* and *rbcL* sequences from type specimens (from recognised herbaria) including for *Lithophyllum*, *Adeylithon*, *Porolithon* and *Sporolithon* (Supplementary Material 1). At the species level, we were unable to match our collections to any sequences of type specimens as these were either different to the type sequence, or unavailable; we therefore referred to specimens from our collection with the suffix “cf.”, which refers to the species that they most closely resembled based on original species descriptions. The sequence of *Ramicrusta* sp., which is not included in the concatenated tree, had 96.2% match to the sequence of *Ramicrusta textilis* (MT215161.1) in *psb*A and 97.9% match to the sequence of *Ramicrusta arenea* (JX969780.1) in *rbc*L through a blast search on GenBank. The phylogenetic analyses support the delineation of species that was performed using morphological assessment for the sorting of the CCA collection used in this study.

**Table 2:** Taxonomic, morphological characters and ecological distributions (habitat types, irradiance and relative abundance across the Great Barrier Reef) of CCA species tested in this study.

Species	Family/Sub-family	Collection site	Key morpho-anatomical characteristics	Distribution		Relative abundance (GBR shelf)			Herbarium numbers	Genbank		Reference (Distribution and GBR abundance)
				Habitat	Irradiance level	Inner	Mid	Outer		psbA	rbcL	
*Lithothamnion* cf. *proliferum*	Hapalidiaceae	Davies Reef	Branching. Lobed branches; very smooth surface; multiporate conceptacles; cell fusions	Crevices, caves	Low	Rare	Rare	Rare	DP-2428	OP830448	OP830463	Dean et al.^15^
*Melyvonnea* cf. *madagascariensis*	Mesophyllumaceae	Davies Reef	Branching. Surface with protuberances; multiporate conceptacles; coaxial hypothallus and cell fusions	Shallow–deep reef	Low–mid	Rare	Common	Rare	DP-2493; DP-2436	OP830452	OP830467	Dean et al.^15^, G.D.-P. pers. obs
*Adeylithon* cf. *bosencei*	Hydrolithoideae	Davies Reef	Branching. Cylindrical branches; strongly tessellate surface; trichocytes in loosely defined fields; cell fusions	Shallow–deep reef	Low–high	Rare	Rare	Common	DP-2438	OP830454	OP830469	Peña et al.^49^, G.D.-P. pers. obs
*Hydrolithon* cf. *reinboldii*	Hydrolithoideae	Palm Island Group	Encrusting. Knobby protuberances; strongly tessellate surface; dimerous hypothallus; cell fusions	Shallow–deep reef	Mid	Common	Moderate	Moderate	DP-2526	OP830457	OP830472	Dean et al.^15^, G.D.-P. pers. obs
*Amphiroa* cf. *foliacea*	Lithophylloideae	Davies Reef	Branching. Articulated (geniculate); cylindrical to flattened branches; secondary pit connections	Shallow–mid reef	Mid–high	Rare	Moderate	Moderate	DP-2437	OP830453	OP830468	G.D.-P. pers. obs
*Lithophyllum* cf. *insipidum*	Lithophylloideae	Palm Island Group	Encrusting. Dimerous thallus; strongly tessellate surface; secondary pit connections present	Crest, shallow reef	Mid–high	Rare	Common	Common	DP-2559	OP830456	OP830471	Dean et al.^15^
*Lithophyllum* cf. *kotschyanum*	Lithophylloideae	Davies Reef	Branching. Thick and robust branches; slightly tessellate surface; secondary pit connections	Reef crest	Mid—high	Moderate	Moderate	Common	DP-2434	OP830451	OP830466	Dean et al.^15^
*Lithophyllum* cf. *pygmaeum*	Lithophylloideae	Davies Reef	Branching. Pointy to round branches; slightly tessellate surface; secondary pit connections	Crest, shallow reef	Mid–high	Rare	Moderate	Moderate	DP-2430	OP830449	OP830464	Dean et al.^15^
*Titanoderma* cf. *tessellatum*	Lithophylloideae	Davies Reef	Encrusting. Concentric whorls; large green–brown conceptacles; secondary pit connections	Shallow–deep reef	Low–mid	Rare	Rare	Rare	DP-2427	OP830447	OP830462	Dean et al.^15^
*Neogoniolithon* cf. *fosliei*	Neogoniolithoideae	Davies Reef	Encrusting. Very large conceptacles; skin chicken like surface; individual trichocytes; cell fusions	Reef crest	High	Rare	Common	Common	DP-2489-2	OP830450	OP830465	Dean et al.^15^
*Porolithon* cf. *onkodes* "Orange"	Metagoniolithoideae	Davies Reef	Encrusting. ‘Orange’ species in *Porolithon* cf. *onkodes* complex; trichocytes in well-defined fields; cell fusions	Reef crest	High	Moderate	Common	Common	DP-2423	OP830444	OP830460	Dean et al.^15^, G.D.-P. pers. obs
*Porolithon* cf. *onkodes* "Chalky"	Metagoniolithoideae	Davies Reef	Encrusting. ‘Chalky’ species in *Porolithon* cf. *onkodes* complex; trichocytes in well-defined fields; cell fusions	Reef crest	High	Moderate	Common	Common	DP-2425	OP830445	OP830460	Dean et al.^15^, G.D.-P. pers. obs
*Porolithon* cf. *onkodes* "Yellow conceptacles"	Metagoniolithoideae	Palm Island Group	Encrusting. Conspicuous yellow-green conceptacles; trichocytes in well-defined fields; cell fusions	Reef crest	High	Common	Rare	Rare	DP-2467	OP830446	OP830461	G.D.-P. pers. obs
*Sporolithon* sp.	Sporolithaceae	Davies Reef	Encrusting. Thick thallus; smooth surface; presence of sori	Crevices, caves	Low	Moderate	Rare	Rare	DP-2439	OP830455	OP830470	Dean et al.^15^, G.D.-P. pers. obs
*Ramicrusta* sp.	Peyssonneliaceae	Davies Reef	Encrusting. Thick thallus only partially calcified (surface soft)	Crevices, caves	Low	Rare	Moderate	Moderate	DP-2435	OP830458	OP830473	G.D.-P. pers. obs

Herbarium numbers refer to the collection of Diaz-Pulido at Griffith University, Brisbane, Australia. Representative sequences of each CCA species for the *psb*A and *rbc*L gene regions were submitted to GeneBank. G.D.-P. refers to Guillermo Diaz-Pulido.

### CCA inductive properties across coral species

A total of 15 coral species across 5 taxonomic families were tested, and there was a significant effect of CCA treatment on settlement across all coral species (Kruskal–Wallis: H = 876.062, df = 17, *p* < 0.001). Averaging across all coral species (n_species_ = 15, n_assay_ = 165 per CCA species), members of the CCA family Lithophyllaceae induced the highest settlement (~ 68–77%) and was comparable to settlement in the coral rubble treatment (64.8 ± 2.5%; see Supplementary Material 2 for coral species level results). Dunn’s pairwise tests showed that settlement in the *P*. cf. *onkodes* “Orange”, *L.* cf. *proliferum*, *L.* cf. *pygmaeum*, *P*. cf. *onkodes* “Chalky” and *A.* cf. *foliacea* were significantly lower than the coral rubble treatment (*p* < 0.05; Supplementary Material 2) while settlement in response to *A.* cf. *foliacea* was similar to that found in the sterile aragonite treatment (*p* > 0.05).

*Titanoderma* cf. *tessellatum* induced settlement of > 75% in 11 out of the 15 coral species, with moderate settlement in *C. aspera* (53.3 ± 7.6%), *M. aequituberculata* (59.7 ± 11.4%), *L. corymbosa* (62.5%) and *F. fungites* (13.8%; Table 3). Overall, *T.* cf. *tessellatum*, *L.* cf. *insipidum* and *L.* cf. *kotschyanum* were the most effective inducers based on mean settlement (see Supplementary Material 2 for pairwise comparisons). However, settlement in non-acroporid species (*C. furcata*, *M. elephantotus*, *P. daedalea*, *L. corymbosa*, and *F. fungites*) was consistently higher in the coral rubble and *Ramicrusta* sp. treatments than in the aforementioned CCA species (Table 3). *Amphiroa* cf. *foliacea* was the weakest inducer for settlement when all coral species were considered (Supplementary Material 2). While *L.* cf. *pygmaneum* and *P.* cf. *onkodes* “Chalky” performed well in most *Acropora* spp. assays and attained settlement of up to > 75%, they do not perform as well across non-*Acropora* taxa (Table 3).

**Table 3:**
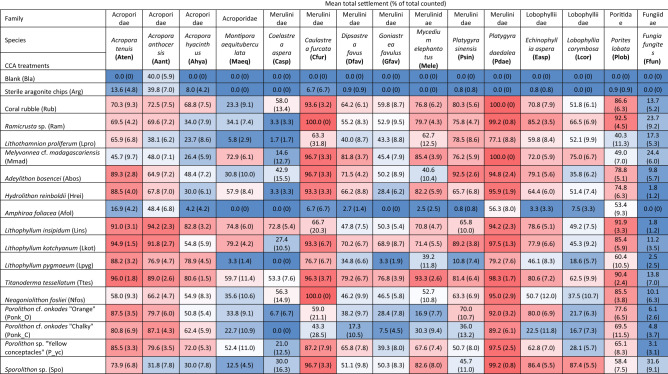
Mean total settlement (%; SE in parentheses) of coral larvae from 15 species across 5 taxonomic families when presented with 15 common encrusting red algal species across 8 taxonomic families from the Great Barrier Reef.

Coral and CCA abbreviations are provided in parentheses. Cells with warmer colours represent higher settlement values. Refer to Table 1 for details on coral larval species.

Larval settlement to the living surface of CCA was highly variable across all coral and CCA species tested, with live-surface settlement ranging from 0 to 56.3% across coral species, for between 0 and 13 CCA treatments (Supplementary Material 2). Species in the Acroporidae and Poritidae had the highest settlement onto the living surface of CCA and are detailed in family-level descriptions in Supplementary Material 2.

### Settlement trends of coral species and families to common CCA and their ecological traits

**Figure 3:**
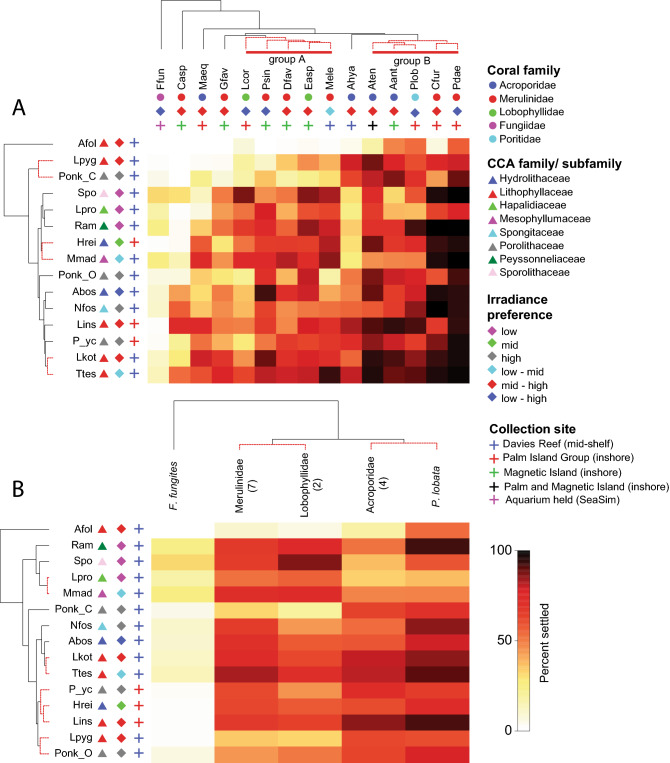
Shade plots ordered by cluster analyses dendrogram of coral and CCA taxa at the (**A**) coral species level and (**B**) coral family level (number of species represented per family indicated in parentheses). Cluster analyses were performed using the Bray–Curtis resemblance matrix for coral taxa and index of association matrix for CCA taxa, on the total settlement data averaged at the coral species and family level. Red dashed lines on the coral and CCA dendrograms reflect distinct groups as determined by SIMPROF analyses. Symbols corresponding to CCA and coral taxonomic families, irradiance preferences and collection sites (see legend) are included after the species abbreviations (See Table 3 for full species names).

Multivariate analyses using settlement data averaged at both the coral species and family levels, were performed to elucidate any ecologically relevant settlement trends associated with taxonomic grouping, irradiance preference, and CCA collection site. At the coral species level, two statistically distinct groups were found: group A was comprised of members of the Lobophyllidae and Merulinidae (*L. corymbosa*, *E. aspera*, *P. sinensis*, *D. favus*, and *M. elephantotus*), while group B comprised members of the Acroporidae, Merulinae and Poritidae (*A. tenuis*, *A. anthocercis*, *C. furcata*, *P. daedalea* and *P. lobata*; Fig. 3A, see Supplementary Material 3 for habitat and depth distribution of studied coral species). SIMPER analyses showed that both groups had high within-group similarity of > 90%, with a between-group comparison (dissimilarity = 12.4%) showing higher average settlement of group B corals to CCAs adapted to living under high irradiance (*A.* cf. *foliacea*, *P.* cf. *onkodes* “Chalky”, *L.* cf. *pygmaeum*, *P.* cf. *onkodes* “Orange”, *N.* cf. *fosliei*, *Porolithon* sp. “Yellow conceptacles” and *A.* cf. *boscensei*, contributing 65% to the between group dissimilarity). This trend corresponded to the structure in the CCA species dendrogram (Fig. 3A). Of note, two large clusters were recovered, one of which comprised CCA that prefer low-mid irradiance (*Sporolithon* sp., *L.* cf. *proliferum*, *Ramicrusta* sp., *H.* cf. *reinboldii* and *M.* cf. *madagascariensis*), and another that comprised mid-high irradiance adapted CCAs (*P.* cf. *onkodes* “Orange”, *A.* cf. *bosencei*, *N.* cf. *fosliei*, *L.* cf. *insipidum*, *Porolithon* sp. “Yellow conceptacles”, *L.* cf. *kotschyanum*)*,* and an outlier *T.* cf. *tessellatum* that prefers low-mid irradiance. *Titanoderma* cf. *tessellatum* and *L.* cf. *kotschyanum* formed a distinct SIMPROF cluster, reflecting their broadly inductive properties across the 15 coral species tested (Fig. 3A). *Lithophyllum* cf. *pygmaeum* and *P.* cf. *onkodes* “Chalky”, which also formed a distinct cluster induced higher settlement in group B corals (Fig. 3A). *Amphiroa* cf. *foliacea* was an outlier that induced settlement in only some members of group A corals. There was no clear trend in the CCA species dendrogram when the CCA family and collection site were considered (Fig. 3A).

At the coral family level, the Merulinidae and Lobophyllidae formed a cluster distinct from the Acroporidae and Poritidae (Fig. 3B). SIMPER analysis showed that both clusters had high within group similarity of > 94%, with between group dissimilarity of 9.56%. Similar to the species level analyses, the separation of these clusters could be explained by the irradiance preference of CCAs, with Merulinidae and Lobophyllidae species settling in higher numbers to low-light adapted CCA species (*Sporolithon* sp., *M.* cf. *madagascariensis* and *L.* cf. *proliferum*), while Acroporidae and Poritidae species settled in response to light-adapted CCA species (*P.* cf. *onkodes* “Chalky”, *A.* cf. *foliacea*, *L.* cf. *pygmaeum*, *P.* cf. *onkodes* “Orange” and *L.* cf. *insipidum*); the aforementioned 8 CCA species contributed to 72% of the between-group dissimilarity. The CCA species dendrogram recovered 2 major clusters and identified *A.* cf. *foliacea* as an outlier. Similar to the species-level analyses, the first cluster comprised of CCA species that are adapted to low irradiance (*Ramicrusta* sp., *Sporolithon* sp., *L.* cf. *proliferum* and *M.* cf. *madagascariensis)*, while the other cluster comprised species that are adapted to medium to high light (Fig. 3B).

## Discussion

Knowledge of the processes that underpin larval settlement behavior is critical for understanding how coral populations are maintained and recover following mortality events^24^. Our study tested the settlement of 15 common coral species against 15 species of coralline red algae from the GBR, and represents one of the most comprehensive studies undertaken to investigate the intimate relationships between coral larvae and crustose coralline algae (CCA) on coral reefs. We found that coral larval settlement to CCA species varied considerably amongst species, even within the same coral family and genus. This variability notwithstanding, our experiment confirmed the alga *Titanoderma* cf. *tessellatum* as a strong inducer of larvae settlement across Acroporidae and Merulinidae^8^. We also identified previously unknown species of CCA that are important inducers of larval settlement in the coral family Lobophyllidae, and in *Fungia fungites* and *Porites lobata*. We demonstrate that coral larval behaviour in response to CCA is highly dependent on the habitat of origin, with coral species from low-irradiance habitats settling in higher proportion on CCA adapted to similar light environments. Although intuitive, this has not been consistently demonstrated across coral and algal species (but see^25^). Our study provides important foundational information and identifies optimal coral-CCA associations that can be used to produce coral spat for reef restoration across a broad range of taxonomic families.

Larval settlement to CCA species varied considerably among and within the coral families examined. Within the Acroporidae, the plating coral *M. aequituberculata,* which is common around the turbid inshore reefs of the Great Barrier Reef (GBR;^26,27^), displayed settlement responses that were distinct from three *Acropora* spp. tested, which settled well (> 75%) in response to CCA species that are adapted to living in high light and high wave-energy environments (*Porolithon* spp. and *Lithophyllum* cf. *pygmaeum*)*.* Interestingly, out of the three *Porolithon* species tested, *M. aequituberculata* settled in higher numbers to *P.* cf. *onkodes* “Yellow conceptacles” (~ 52%) when compared to the other two *Porolithon* spp. (< 35%). Both the coral and CCA species were collected from inshore reefs, which may suggest that some of the variability in CCA-larval interactions may be related to the type of environment that the species pair share.

Intra-generic differences in larval settlement behaviour were also found within the *Acropora* and *Platygyra*. For example, *A. hyacinthus,* which is typically found on reef crest environments, settled up to 50% less on moderate‒low light adapted CCAs compared to *A. tenuis* and *A. anthocercis*, both of which occur in deeper and inshore habitats, indicating a potential innate ability of *A. hyacinthus* larvae to discriminate between CCA adapted to differing irradiance environments. This behaviour could significantly influence habitat selection by *A. hyacinthus* and may, consequently, influence post-settlement survival, growth, and ultimately species distributions on the reef. Within the genus *Platygyra*, *P. daedalea* is a generalist that settled in response to all the CCA species tested except *A.* cf. *foliacea*, while *P. sinensis* was more selective. As *P. daedalea* and *P. sinensis* co-occur in similar habitats across the GBR, this variability in settlement response may be driven by species-specific associations, as seen in other sympatric congeneric coral species^16^.

Larval settlement also varied widely depending on the taxonomic subfamilies of coralline algae considered. For example, while CCA species within the Metagoniolithoideae (which includes *Porolithon* spp.) were moderate to good (> 63% settlement) inducers for the Acroporidae, they did not perform as well for corals from other taxonomic families. For instance, in the Lobophyllidae, while none of the *Porolithon* species induced settlement of > 50% (and recording as low as 20% in *P.* cf. *onkodes* “Chalky”), *Sporolithon* sp., *Ramicrusta* sp., and *M.* cf. *madagascariensis* induced settlement of up to 87%. A similar trend was found for the Merulinidae and *Fungia fungites*, again highlighting the poor performance of *Porolithon* spp. in inducing larval settlement in these groups. The reverse is however true for the Acroporidae, whereby the latter 3 CCA species did not induce settlement over 60%. Interestingly, *Sporolithon* sp., *Ramicrusta* sp., and *M.* cf. *madagascariensis* are all adapted to living in habitats having low irradiances. It seems therefore that the coral taxa that occur in deeper or more turbid waters (e.g. inshore compared to mid-shelf reefs), seem to prefer CCA from low-light environments. At the broader level, these observed larval behaviours, and thus potential habitat selectivity, suggests that habitat selection and subsequent ecological partitioning of species on reefal habitats could occur as early as the larval settlement phase (see^28^).

The subfamily Lithophylloideae is overall the best inducer of coral settlement across the range of coral species and families tested, in particular for the Acroporidae. This finding corroborates a recent study in Guam using *Acropora surculosa*, which found that larvae settled 9 × more on an undescribed Lithophylloideae sp.1, compared to 26 other CCA species presented together on conditioned substrate^29^. Interestingly, CCA within the genus *Lithophyllum*, and the subfamily Lithophylloideae more broadly, include species that are morphologically diverse and that illicit a broad range of settlement responses in corals. Within the genus *Lithophyllum*, *L.* cf. *kotschyanum* (with short protuberances) and *L.* cf. *insipidum* (smooth encrusting), consistently out-performed *L.* cf. *pygmaeum* (longer branches) across most of the coral species tested, except for *Acropora* and some generalist Merulinidae (e.g. *C. furcata* and *P. daedalea*). The geniculate *A.* cf. *foliacea* was the poorest performing Lithophylloideae in which very low to no settlement was found across most coral species. Despite the variability in coral settlement capabilities across the Lithophylloideae, a species that gave relatively consistent settlement results across the 5 coral taxonomic families was *Titanoderma* cf. *tessellatum*. Why this species of *Titanoderma* is such an effective inducer across several coral taxa is little known, although recent studies indicate that the unique metabolites and microbial community associated with this CCA may be responsible for the settlement of some species of *Acropora*^22,30,31^.

Individual CCA species were presented in isolation and therefore may not fully elucidate the preferential behaviour of larvae as compared with a choice experiment. For example, *Neogoniolithon fosliei* has been shown to induce 15 × less settlement compared to *Titanoderma prototypum* in species of *Acropora* if they were presented together^8^. In our study, fairly high settlement to *N.* cf. *fosliei* (60%) was observed, although it remained lower than the congeneric *T.* cf. *tessellatum* (82%). Whether the use of freshly cut CCA chips, which may release cell-bound settlement cues, could have an effect on settlement is unknown, however species of CCA are known to heal injuries rapidly (e.g. < 2 weeks^32^); nevertheless, our method allowed for the isolation of CCA-specific morphogens, which would otherwise complicate the interpretation of inductive cues when larvae are tested in choice experiments (i.e. larvae in CCA choice experiments may recognize water-borne or surface cues from one CCA species but attach elsewhere, masking the origin of the cue). Importantly, our study confirms that coralline algae are, overall, strong inducers of larval settlement across all families of corals investigated, and the strength of the inductive potential not only varies depending on the families of corals and algae considered, but also the type of habitat from which both pairs originate.

The causes of the hierarchy in inductive potential of CCA species are not well understood. External algal morphology doesn’t seem to be a good predictor of coral larval settlement because species with very similar morphology elicit very different settlement responses. For example, both *M.* cf. *madagascariensis* and *L. kotschyanum* have a branching morphology with the presence of protuberances (see Fig. 1b and g) yet they had contrasting effects on *A. anthocercis* settlement. Similarly, smooth species with nearly identical morphological and anatomical features, such as the 3 taxonomically cryptic species within the *Porolithon* led to large differences in settlement responses (i.e. 22 vs. 87% settlement) in some coral species. A greater focus will therefore need to be placed on the accurate identification of CCA, most likely from using a combination of morphological and molecular approaches as performed in this study, for future studies on coral-CCA interactions.

Biological characteristics of some CCA species have been shown to affect coral larval settlement. For example, epithallial shedding (process by which the uppermost, surficial tissue, the epithallus, sloughs off) has been suggested as an important mechanism by which CCA deter coral larval settlement^8^. However, *N.* cf. *fosliei*, a CCA with strong epithallial shedding induced variable settlement across species. Another possible factor influencing variable settlement induction by Lithophylloideae could be the absence (or scarcity) of trichocytes on the thallus surface, as trichocytes may easily come off the CCA surface dislodging settled larvae. CCA from the other subfamilies in the Corallinaceae such as Hydrolithoideae (*Hydrolithon, Adeylithon*), Metagoniolithoideae (*Porolithon*) and Neogoniolithoideae (*Neogoniolithon*) have abundant trichocytes; however, this hypothesis needs further examination. Algal derived metabolites have also been suggested to drive the settlement behavior of coral larvae and could vary amongst CCA species, resulting in hierarchical induction^30,33^. Alternatively, microbially derived cues have also been identified as important inducers of coral larval settlement and these vary amongst CCA taxa^15,31^. It is also possible that cues from both the algal and microbial components interact to modulate coral settlement, as shown for *Acropora* on the GBR^30^, and it is also plausible that the nature of these cues is species-specific, given the large variability in surficial microbiome composition across CCA taxa^22,31,34,35^ and chemical constituents of the alga thallus^36^. Detailed microbiome and metabolomic studies across a range of coral and algal taxa (e.g.^22^) would be useful for a better understanding of the nature of larval settlement behavior and their preferences for CCA.

This study summarises the settlement responses of coral larvae to CCA across a broad range of taxa, both for the corals and algae, and will serve as an important platform from which further detailed investigations into the intimate mechanistic pathways for coral larval settlement could be progressed. Our findings here demonstrate the important role of an understudied CCA, *Sporolithon* sp., on the settlement of corals in the Lobophyllidae and *Fungia fungites*, and further investigations may uncover novel pathways for habitat selection and larval settlement in non-acroporid coral species. While some studies investigating larval settlement behaviours and competencies have previously used *Porolithon onkodes* as a routine biological morphogenic inducer^37,38^, we show here that variable settlement results could inevitably surface due to the cryptic species that are present and currently unresolved in this species complex (e.g.^39^). The same may be true for other coralline algae groups studied here. For reliable and consistent settlement across coral taxa, an alternative option would be to use *Titanoderma* cf. *tessellatum*, which is simpler to identify in the field due to its distinctive morphological characters (i.e. presence of whorls), although detailed taxonomic work is also needed to resolve the taxonomy and test for the presence of cryptic species within this group. Finally, with the world’s coral reefs currently under increasing stress, the optimal coral-algal species combinations reported in this study could be utilized to increase the success of larval settlement to generate healthy spat across a wide range of coral families to supply corals for reef restoration.

## Materials and methods

To assess the settlement preferences of coral larvae to CCA, we collected gravid coral colonies and CCA from several localities on the central GBR and transferred them to the National Sea Simulator aquarium facility at the Australian Institute of Marine Science, Townsville (AIMS SeaSim). Settlement assays were performed over a period of ~ 48 h under controlled environmental conditions to test the efficacy of each CCA species in inducing coral larval settlement.

### Coral collection, spawning and larval culture

Coral spawning was performed in October and November 2021 (refer to Table 1 for the list of species, reproductive modes and spawning details) to capture inshore and offshore GBR spawning, respectively. Reproductively mature corals were collected from depths of 1–9 m from reefal habitats around Magnetic Island (19°07′45.78″S 146°52′40.14″E), the Palm Island Group (18°45′56.4″S 146°32′2.58″E) and Davies Reef (18°49′13.5″S 147°38′40.32″E), central GBR between the 9th to 20th of October and 14th to 21st of November 2021 (GBRMPA Permit G21/45348.1). Habitats around Magnetic Island and the Palm Island group are representative of fringing reefs around inshore islands of the GBR, and Davies Reef is representative of a low-turbidity mid-shelf reef.

Approximately 1 week before the full moon, mature coral colonies were determined by visual inspection of pigmented eggs within the coral tissue in situ, and whole colonies, or fragments, collected using a hammer and chisel on SCUBA. As eggs were small (~ 200 μm) and unpigmented in the gonochoric coral *Porites lobata*, small fragments (n = 4–5; ~ 5 × 5 mm) containing live tissue were stained in 10 mL of Neutral Red for 20 min (following^40^) to highlight the mesenteries, ovaries and testes, and observed under a stereo microscope to confirm the sex and maturity of the colonies prior to collection. Coral colonies and fragments were left on the reef until the morning of transfer to the research facility, where they were transported in 70L bins (1‒4 colonies per bin) receiving constant flow-through seawater on board the vessel over a period of 4‒6 h. Upon reaching the AIMS SeaSim, corals were held in outdoor semi-recirculating aquaria that received new input of 1 µm filtered seawater (FSW) at a rate of ~ 3 turnovers per day and that profiled the ambient temperature experienced at mid-shelf reefs, based on the historic daily mean reef temperature at Davies Reef measured at 4 m water depth between 1991 and 2012 (~ 27.2 °C; see Supplementary Material 2, Fig. S4, for temperature profile).

Depending on species and colony sizes, between 10 and 20 individual colonies were held in each aquarium (deep aquaria: 100 × 280 × 50 cm, 2000 L; shallow aquaria: 80 × 140 × 28 cm, 280L). All aquaria received natural sunlight (midday max intensity ~ 200 μmol quanta m^−2^ s^−1^) and photoperiod (12‒13 h daylight) and were fitted with 2 gyres (Maxspect 350 series) at each end of the aquarium to provide consistent water circulation across aquaria.

Corals were monitored throughout the predicted evenings of spawning and individual colonies were isolated in 60 L aquaria when setting of gametes was observed. For coral species that released egg-sperm bundles, gametes were collected within 1 h of release by skimming from the water surface using a clean plastic cup and plastic pipettes. The bundles were gently agitated and filtered through a 106 μm mesh screen to separate eggs and sperm. Eggs were washed with FSW. For each coral species, gametes from all parent colonies were pooled for cross fertilization in a 60 L aquarium at approximately 1 × 10^6^ sperm mL^−1^. After 1 h, embryos were gently rinsed in FSW to remove excess sperm and transferred to either 500 L or 70 L flow-through culture tanks at a stocking density of approximately 0.3 larvae mL^−1^. For the gonochoric species (*P. lobata* and *Fungia fungites*), and hermaphroditic species that release eggs and sperm separately (*Lobophyllia corymbosa* and *Goniastrea favulus*), fertilization was performed in 60 L aquaria by transferring water containing sperm across aquaria and embryos transferred to culture tanks when first signs of cleavage were detected (~ after 30–45 min). Gentle aeration was provided around the culture tank standpipes to prevent embryos from sticking to the outlet filters (106–212 μm mesh depending on species embryo sizes) and aeration was gradually increased after 24 h (beyond the gastrula stage) to allow for in-water circulation. Larvae were maintained in the culture tanks until used in the settlement experiment.

### Algal collections and identifications

Thirteen common species of non-geniculate crustose coralline algae, a geniculate articulated coralline algal species, and an encrusting calcifying red algal species from the family Peyssonneliaceae, were collected from reefal habitats at Davies Reef and Palm Island Group where coral species co-occurred at depths of 1–10 m, between the 9th and 20th of October 2021 (Fig. 1). Collections were performed on SCUBA using a hammer and chisel. CCA were held in 70 L flow-through aquaria and were subsequently identified and sorted onboard the research vessel. CCA samples were identified based on morphological and anatomical characters, including thallus surficial texture, presence/absence of trichocytes and trichocyte fields, types of reproductive structures (conceptacles), and hypothallus arrangement, using a dissecting microscope and cell connections (fusions and secondary pits) under a compound microscope (e.g.^41,42^) and, where possible, species-level identification assigned. Representative individuals of the different species used in the experiment were preserved in silica gel for molecular identifications and voucher specimens deposited in the Coral Reef Algae Laboratory at Griffith University, Brisbane. Genomic DNA extraction and amplification followed^43^. Briefly, genomic DNA was extracted using a NucleoSpin Plant II Kit (Macherey–Nagel, Düren, Germany) following the manufacturer’s instructions. Two plastid-encoded markers (*psb*A; psbAF1 and psbAR2 primers and rbcL; F57/R1150 and F993/RrbcStart primers) were amplified to infer phylogenetic relationships of the experimental CCA species^44,45^. Each PCR reaction comprised a 30 μl mixture of 4–12 μl genomic DNA, 1 μl of 10 pmol forward + 1 μl of reverse primers, 0–8 μl distilled water, and 16 μl HelixAmp Ready-2x-Go Series (NanoHelix, Daejeon, Korea).Cycle sequencing was performed by Macrogen (Seoul, South Korea). Detailed description of sequence alignments and phylogenetic analyses are provided in Supplementary Material 1, with the concatenated *psb*A and *rbc*L tree presented here. Newly generated sequences were deposited in GenBank (Table 2).

All CCA species were collected from Davies Reef except for *Hydrolithon* cf. *reinboldii*, *Lithophyllum* cf. *insipidum* and *Porolithon* cf. *onkodes* “Yellow conceptacles”, which were collected from the Palm Island Group (Table 2). For Davies Reef CCA, species adapted to low-light conditions were collected from crevices and under overhangs and included *Ramicrusta* sp., *Lithothamnion* cf. *proliferum* and *Sporolithon* sp.; other species occurred commonly on shallow reef crests exposed to high light and high flow, and included species of *Porolithon* spp., *Neogoniolithon* cf*. fosliei*, *Adeylithon* cf. *bosencei* and *Lithophyllum* cf. *pygmaeum*. *Lithophyllum* cf. *kotschyanum* was collected from a field of branching acroporid coral rubble at ~ 3 m. *Titanoderma* cf. *tessellatum* and *Melyvonnea* cf. *madagascariensis* occurred in habitats with moderate illumination; *T.* cf. *tessellatum* was found on vertical walls or at the intersection of light–dark habitats (i.e. at the edge of overhangs) while *M.* cf. *madagascariensis* was found growing on deeper substrates (~ 8–9 m). The articulated coralline alga *Amphiroa* cf. *foliacea* was collected from the front of Davies Reef at 5‒6 m depth. Collectively, these CCA species were chosen because they are among the most common taxa on the GBR and they encompass the major orders of the subclass Corallinophycidae (Rhodophyta). ‘Coral rubble’ was also included as a treatment as this presents a common reefal substrate, comprising diverse community of potential alternative inducers for coral larvae^46^. Coral rubble chips were prepared from recently dead rubble of massive *Porites* sp. covered with thin microbial and algal biofilms (cyanobacteria, diatoms), brown algae *Sphacelaria* sp., green algae *Cladophora* sp., and red algae *Polysiphonia* spp., *Ceramium* spp., and ~ 40% cover of mixed CCA (characterized from n = 8 fragments).

CCA were transported in flow-through 70L bins, similar to the method used for corals, to AIMS SeaSim where they were held in indoor semi-recirculating aquaria (80 × 140 × 28 cm, 280 L) with water exchange flow rates and circulation as described above. Aquaria were outfitted with 2 LED panel lights, with a lighting profile comprising a linear ramp-up period of 6.5 h from darkness (05:30 h) to a maximum of ~ 120 μmol quanta m^−2^ s^−1^ (12:00 h) and a ramp-down over 6.5 h to darkness (18:30 h). Light intensities for low-light adapted (max midday 12.7‒15 μmol quanta m^−2^ s^−1^; LI-COR LI-250A) and moderate-light adapted (max midday 56‒58 μmol quanta m^−2^ s^−1^) CCA species were controlled using one to several 50% shade cloths in the holding tank.

### CCA maintenance and settlement assays

To assess settlement responses of coral larval species to the suite of CCA species, assays using live CCA chips were performed during the October and November 2021 spawning periods. CCA fragments were cut into 10 × 10 mm pieces using a wet diamond band saw (Gryphon), glued onto a poly-vinyl-chloride (PVC) rack, and allowed to recover for at least 2-weeks in their respective holding tanks. Immediately prior (< 1 h) to setting up settlement assays, CCA fragments were resized to 5 × 5 mm pieces to minimize any water quality issues (e.g. excessive organic loading and anoxic conditions) during the assays that could result from high biomass. Experimental treatments included the 15 algal species, the coral rubble treatment, a negative control that did not contain any substrate apart from the plastic surfaces of wells (blanks), and a procedural control that had a 5 × 5 mm fragment of sterile aragonite (autoclaved at 120 °C for 20 min).

Settlement assays were performed in wells (Costar® 6-well plates) filled with 10 ml of 0.1 μm FSW. Ten active and normal swimming larvae and one treatment chip (placed in the centre of the well with the live tissue facing up) were added to each well. The experiment was conducted in a temperature-controlled room (27.2 °C). The position of CCA treatments across wells was randomized to minimize any well or plate positioning bias. LED panel lights were positioned above the plates to maintain the healthy condition of live CCA in the assays with a linear ramp-up period of 6.5 h from darkness (05:30 h) to a maximum of ~ 50 μmol quanta m^−2^ s^−1^ (12:00 h) and a ramp-down over 6.5 h to darkness (18:30 h). Maximum illumination intensity of ~ 50 μmol quanta m^−2^ s^−1^ was selected to emulate light conditions on benthic substrates onto which coral larvae are likely to settle^47^.

Settlement assays were performed over 46‒55 h. Total larval settlement and the position on which larvae had successfully settled (i.e. on live CCA tissue or on the side or underside of CCA, in the matrix of CCA, and on the plastic surface of wells) were recorded (see Supplementary Material 2, Fig. S42, for images of larval settlement endpoints). Settlement was defined as the permanent attachment and metamorphosis of a larva into a primary polyp that is flattened on the oral-aboral axis to form a disc-shaped structure with obvious radial septal mesenteries^17^. Fluorescence was used to assist in the detection of smaller sized larvae, using a stereo microscope fluorescence adaptor (https://nightsea.com/; SFA RB – excitation 440–460 nm, emission filter 500 nm longpass) that excites the larval green fluorescent proteins.

As the same CCA specimens collected in October were used for assays following both spawning periods, any temporal changes in their bioactivity (e.g. from aging or being held in aquaria) were assessed by performing a duplicate assay in November using the coral species *Acropora tenuis*. Similar conditions were applied across the two assays, with the only difference being the testing of 6-day old larvae in October compared with 7-day old larvae in November (Table 1). In addition, assays were repeated for the coral species *Coeloastrea aspera* across three larval ages (5, 8 and 18 d) in October, because 5-day old larvae had low settlement across treatments and thus may not have been competent. Twelve treatment replicates were used in all coral settlement assays, except for 8- and 18-day old *C. aspera* (n = 6) and *Caulastrea furcata* (n = 3; Table 1), and all larvae tested were between 4 and 8 days old unless otherwise stated (Table 1).

### Statistical analyses

To assess broad settlement trends, settlement data for each CCA species were pooled to coral family (Acroporidae n_assays_ = 48; Merulinidae n_assays_ = 69; Lobophyllidae n_assays_ = 24; Poritidae n_assays_ = 12; and Fungiidae n_assays_ = 12). The assay for *A. tenuis* from November was omitted from family level analyses as this species exhibited similar settlement responses across the two test periods (see Supplementary Material 2 for temporal comparisons). Assay data from 5-day old *C. aspera* (Merulinidae) were omitted from the analysis as settlement competency was not reached for this species until day 8. Similarly, assay data from 18-day old *C. aspera* assay were omitted because results were similar to those obtained from 8-day old larvae (Supplementary Material 2) and to keep age consistent with other coral species tested. Additionally, data were pooled across all coral species to identify overall patterns in response to the CCA species. Family-specific settlement data did not meet model assumptions for parametric tests; therefore Kruskal–Wallis and Dunn’s post-hoc pairwise tests with Bonferroni adjustments were performed using the function *kwPlot* in the package ‘GMAMisc’, using the aragonite treatment as an experimental control. Figures were generated using the package ‘ggplot2’. Similarly, coral species level analyses were performed using Kruskal–Wallis and Dunn’s pairwise tests, as assumptions for parametric tests were not met. Only total settlement, regardless of settlement position in the assays, was assessed. General trends of larval settlement onto the living surface of CCA are briefly reported. Univariate analyses were performed in R Studio, using R version 4.0.4.

To assess whether groups of coral species or families has specific settlement preferences for CCA species, a multivariate dataset was generated by averaging total settlement in response to each CCA species at (1) the coral species level and (2) the coral family level. Cluster analyses (9999 permutations) were performed separately for coral species- and family-level data, on the Bray–Curtis similarity resemblance matrix (samples), and on the Index of Association similarity resemblance matrix for the CCA species (variables). Similarity profile tests (SIMPROF) were concurrently performed to identify statistically distinct clusters from the analyses. To visualize any trends in settlement by group, shade plots of average percent settlement, ordered by coral and CCA dendrograms, were plotted to include CCA family, irradiance preference and collection sites for each CCA species. CCA habitat and irradiance preferences were determined from^48,49^ and personal observations by Diaz-Pulido, where high irradiance refers to shallow reef crest environments, low irradiance to crevices and cave habitats, and moderate irradiance to reef slope and wall habitats (Table 2). Similarity percentage (SIMPER) analysis on the resulting SIMPROF groups identified CCA species that contributed to ~ 70% of the between-group dissimilarity. Multivariate analyses were performed in PRIMER v7.

## Supplementary Information

Supplementary Information 1.

Supplementary Information 2.

## Data Availability

All summary data generated in the study are provided as tables in the main article and the Electronic Supplementary Materials (ECM). Raw data is available from the Australian Institute of Marine Science Data Centre repository (https://apps.aims.gov.au/metadata/search). Molecular data for CCA species identifications used during the current study were uploaded to GenBank under accession numbers OP830444 to OP830473. Data from GenBank can be accessed by following instructions in the article https://academic.oup.com/nar/article/45/D1/D37/2605704.
