# Supplementary material for: Hierarchical settlement behaviours of coral larvae to common coralline algae
Source: Sci Rep. 2023 Apr 9;13:5795. doi: 10.1038/s41598-023-32676-4 (PMC10083175; doi:10.1038/s41598-023-32676-4)
Supplement: Supplementary file 1 — Supplementary Information 1. [file 41598_2023_32676_MOESM1_ESM.pdf]

Supplementary material 1: Hierarchical settlement behaviours of coral larvae to common coralline algae – CCA molecular identifications and species delineation

Authors: Abdul Wahab MA, Ferguson S, Snekkevik V, McCutchan G, Jeong S, Severati A, Randall C, Negri A, Diaz-Pulido G

Sequence and phylogenetic analyses of CCA

To confirm the identity of our algal specimens (Table S1) and determine how close our sequences are with type specimens, and explore phylogenetic relationships between taxa, the newly generated sequences (this study) and existing sequences obtained from GenBank were assembled into phylogenetic trees. Sequences were aligned with MEGA7 (Kumar et al. 2016). Phylogenetic and molecular evolutionary analyses for maximum likelihood (ML) analysis were conducted under the GTR+G+I model of sequence evolution with 1000 bootstrap replications using RAxMLGUI v1.5 (Stamatakis 2006; Stamatakis et al. 2008; Silvestro and Michalak 2012). PartitionFinder 2 (Lanfear et al. 2017) was used to determine the best partition scheme and model of evolution as implemented by RAxML. The individual *psb*A and *rbc*L sequence data included our sequences, and sequences from members of all five extant Corallinophycidae orders (Corallinales, Corallinapetrales, Hapalidiales, Rhodogorgonales and Sporolithales) to resolve phylogenetic relationships within the subclass Corallinophycidae. The available type sequences on GenBank were included to provide reliable taxonomic reference points for existing names. The concatenated *psb*A and *rbc*L alignment was also created for each of the five orders. *Acrochaetium arcuatum*, *Audouinella hermannii*, *Ceramium kondoi*, *Gracilaria secunda*, *Nemalion* sp. *Palmaria palmata* and *Thorea violacea* were chose as outgroups.

Table S1: List of coralline algae specimens used in molecular analyses and details of collection and herbarium numbers, collection data (locality, date, depth and collectors: GDP = G. Diaz-Pulido, SJ = Saskia Jurriaans, JMP = Jose Montalvo Proano; MAW = Muhammad Abdul Wahab), and whether or not psbA and rbcL gene sequences were successfully obtained (y= yes, n= no).

No.	Species name	Collection No.	Herbarium No.	Collection data	*psb*A	*rbc*L
Order: Corallinales						
1	*Adeylithon bosencei*	17	DP-2438	Davies Reef, NE side, 10.10.2021, 2-4m depth, Coll. GDP	y	y
2	*Amphiroa* cf. *foliacea*	16	DP-2437	Davies Reef, SW side, 12.10.2021, 4m, Coll. GDP	y	y
3	*Hydrolithon* cf. *reinboldii*	104	DP-2526	Havannah Island, Reef flat, SE side, 16.10.2021, 2-3m depth, Coll. SJ & JMP	y	y
4	*Lithophyllum* cf. *insipidum*	137	DP-2559	Havannah Island, Reef flat, SE side,16.10.2021, 2-3m depth, Coll. SJ & JMP	y	y
5	*Lithophyllum* cf. *kotchysanum*	13	DP-2434	Davies Reef, Leeward W side, 4m, 10.10.2021, Coll. GDP	y	y
6	*Lithophyllum* cf. *pygmaeum*	9	DP-2430	Davies Reef, Leeward W side, 10.10.2021, 4m, Coll. GDP	y	y
7	*Neogoniolithon* cf. *fosliei*	67	DP-2489-2	Davies Reef, N side, 13.10.2021, 3m, Coll. GDP	y	y

8	*Porolithon* cf. *onkodes* "chalky"	4	DP-2425	Davies Reef, S side, 11.10.2021, 2m depth, Coll. GDP	y	y
9	*Porolithon* cf. onkodes "orange"	2	DP-2423	Davies Reef, Leeward W side, 10.10.2021, 2m, Coll. GDP	y	y
10	*Porolithon* cf. *onkodes* "yellow conceptacles"	45	DP-2467	Davies Reef, N side, 13.10.2021, 3m, Coll. GDP	y	y
11	*Titanoderma* cf. *tessellatum*	6	DP-2427	Davies Reef, N side, 10.10.2021, 4m depth, Coll. GDP	y	y
Order: Hapalidiales						
12	*Lithothamnion* cf. *proliferum*	7	DP-2428	Davies Reef, Leeward W side, 10.10.2021, 6m depth, Coll. GDP	y	y
13	*Melyvonnea* cf. *madagascariensis*	15	DP-2436	Davies Reef, S side, 12.x.2021, 5m, Coll. MAW	n	y
14	*Melyvonnea* cf. *madagascariensis*	71	DP-2493	Davies Reef, Leeward side, 12.10.2021, 4m, Coll. GDP	y	n
Order: Sporolithales						
15	*Sporolithon* sp.	18	DP-2439	Davies Reef, SW side, 12.10.2021, 6m depth, Coll. GDP	y	y
Order: Peyssonneliales						
16	*Ramicrusta* sp.	14	DP-2435	Davies Reef, NE side, 10.10.2021, 4m, Coll. GDP	y	y

A total of 30 sequences were newly generated: 15 *psb*A (368-972bp) and 15 *rbc*L (349-758 bp). The concatenated dataset had 51 taxa with sequences ranging from 654 to 1173 nucleotides. Phylogenetic trees inferred from the *psb*A and *rbc*L sequences showed similar topologies in maximum-likelihood analyses. In total, eleven sequences were identified in the order Corallinales, two in the order Hapalidiales, one in the order Sporolithales (Figures S1-S2). The sequence from the order Peyssonneliales was not included in the phylogenetic tree for this analysis.

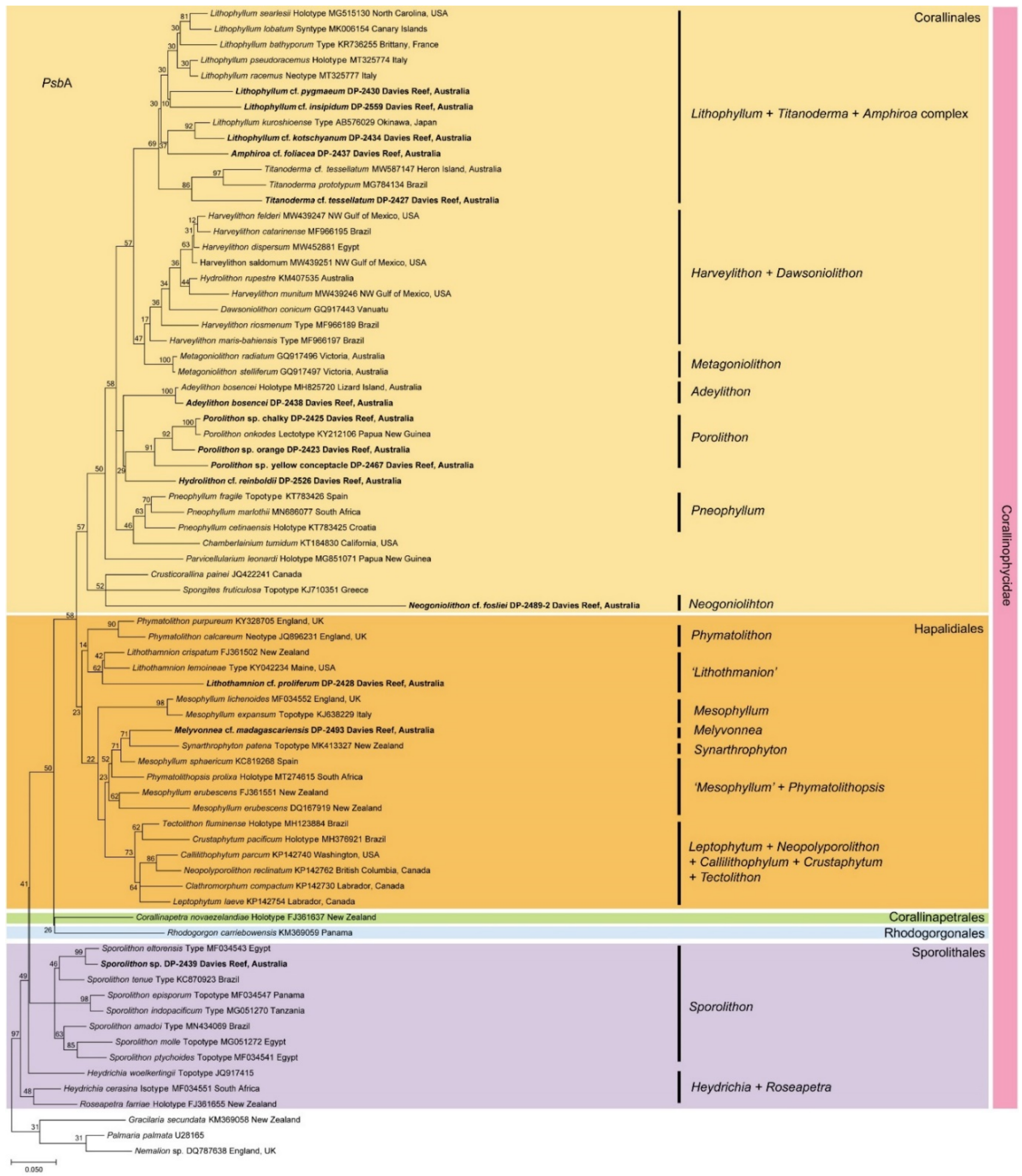

Figure S1: Phylogenetic tree based on Maximum Likelihood (ML) analysis of *psb*A sequences. Values above branches denote maximum likelihood bootstrap values (BS) in %. Sequences obtained from this study are in bold.

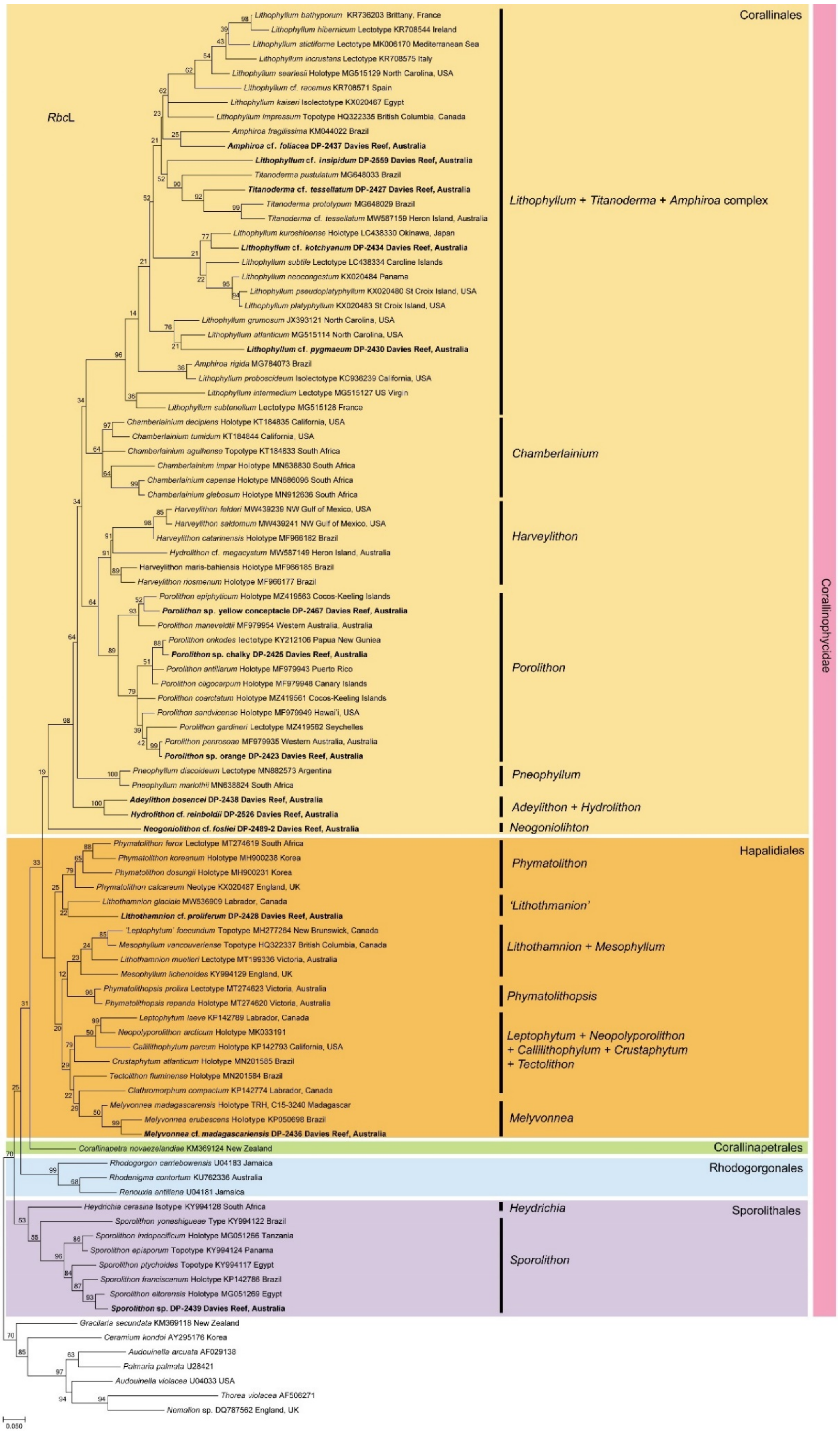

Figure S2: Phylogenetic tree based on Maximum Likelihood (ML) analysis of rbcL sequences. Values above branches denote maximum likelihood bootstrap values (BS) in %. Sequences obtained from this study are in bold.

Supplementary material 2: Hierarchical settlement behaviours of coral larvae to common coralline algae – Temporal, species- and family-specific larval settlement patterns

Authors: Abdul Wahab MA, Ferguson S, Snekkevik V, McCutchan G, Jeong S, Severati A, Randall C, Negri A, Diaz-Pulido G

Map of the study area with coral and CCA collection sites

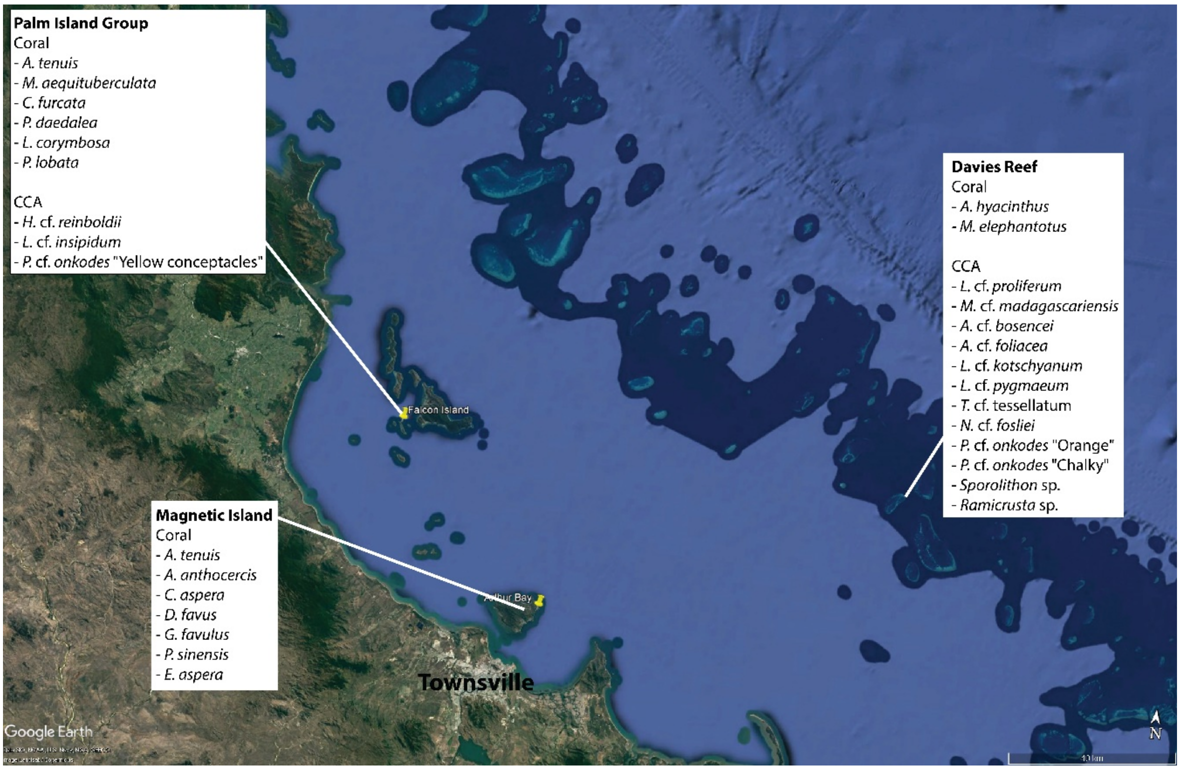

Figure S3: Map of the study area showing collection sites for both coral and crustose coralline algae (CCA) species used for settlement assays. The map was generated in Google Earth 2023 using Landsat/ Copernicus imagery.

20-year average temperature profile of Davies Reef used for maintaining broodstock corals

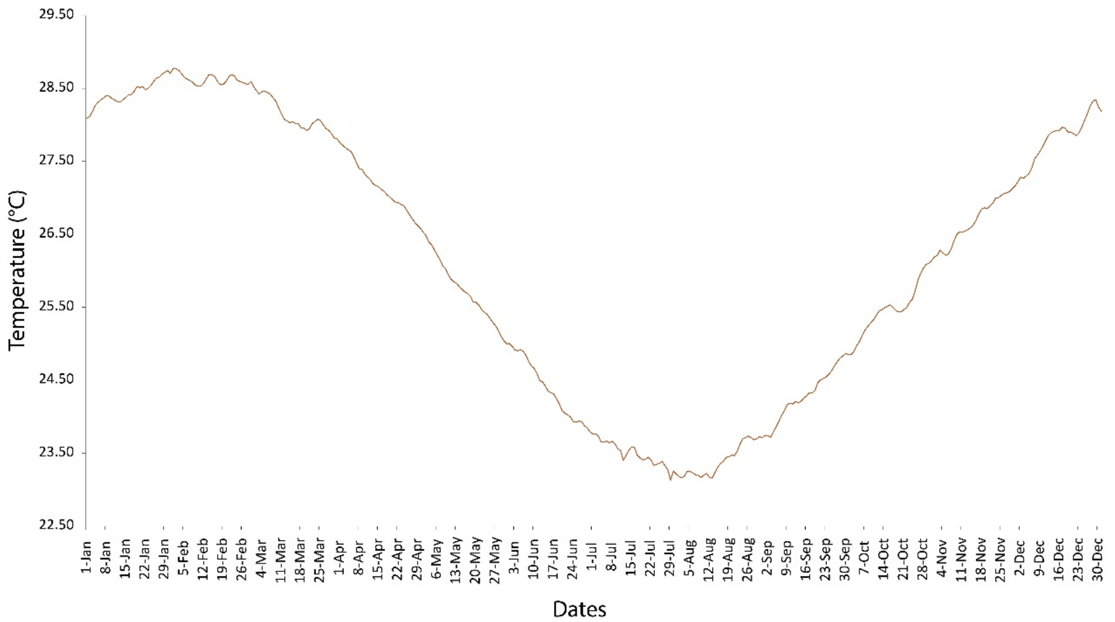

Figure S4: 20-year average temperature profile at Davies Reef that was used to maintain broodstock corals at the National Sea Simulator for spawning. Source: Australian Institute of Marine Science Data Centre.

Settlement inducive properties of CCAs kept in aquaria

Settlement behaviours of *A. tenuis* larvae were investigated in both October and November 2021 to assess if there was a temporal effect on the settlement inducive bioactivity of CCAs that had been maintained in aquaria between spawning periods. There was a significant effect of CCA treatments on larval settlement in both October and November 2021 (Kruskal-Wallis; October: H = 129.781, df = 17, $p < 0.001$, November: H = 96.486, df = 17, $p < 0.001$). Zero settlement in blank treatment wells across both time periods indicates that larvae from both batches were still healthy at the end of the assay (Figure S5 and S6), with all larvae exhibiting normal swimming behaviours; this assumption applies to all subsequent assays performed on other coral larval species.

The inducive properties of CCAs were similar between the two time periods with median larval settlement exceeding 75% for *T.* cf. *tessellatum*, *L.* cf. *kotschyanum*, *L.* cf. *insipidum*, *A.* cf. *boscensei*, *H.* cf. *reinboldii*, *L.* cf. *pygmaeum*, *P.* cf. *onkodes* “Orange”, *P.* cf. *onkodes* “Yellow conceptacles”, *P.* cf. *onkodes* “Chalky”, *Sporolithon* sp., and coral rubble (Figure S5 and S6). Larval settlement to some of the CCA treatments that had low (<50%) to moderate (between 50 to 75%) settlement in October were found to be higher in November, reaching >75% settlement that include *M.* cf. *madagascariensis* (1.1× higher mean settlement), *N.* cf. *fosliei* (1.5×), *L.* cf. *proliferum* (1.1x), and *Ramicrusta* sp. (1.3×; Table 3 of main article), and is likely due to larvae (1-day older) in November being less selective as shown by higher settlement in the sterile aragonite treatment (substrate control), with 13.6 ± 4.8% mean settlement recorded in October compared to 50 ± 10.9% settlement in November. In both months, settlement to *A.* cf. *foliacea* remained low (<20%) indicating that this CCA species is not a good inducer for settlement for *A. tenuis* larvae.

In October, most CCA species significantly induced higher levels of settlement when compared to the aragonite control (Dunn’s test, $p < 0.05$; Figure S7), except for *A.* cf. *foliacea*, *M.* cf. *madagascariensis*, *N.* cf. *fosliei*, *L.* cf. *proliferum*, *Sporolithon* sp., *Ramicrusta* sp. and coral rubble. In November, the differentiation between CCA and aragonite treatments was less prominent with only *Ramicrusta* sp., *A.* cf. *bosencei* and the coral treatment inducing higher settlement than the

aragonite control (Dunn's test, $p < 0.05$). Of note, all CCA species tested except for *A.* cf. *foliacea* induced similar levels of total settlement in both months when compared to the coral rubble treatment (Figure S5 and S6), indicating that the broad bioactivity of CCA chips were not affected when held in artificial aquaria over a period of 1 month.

It is noteworthy that there were changes in settlement patterns of larvae directly to the living surface of CCA chips, between the two experimental periods (Table S2). Specifically, *A. tenuis* larvae settled onto the living surface of most (13) CCA treatment chips in October except for *L.* cf. *proliferum*, *N.* cf. *fosliei* and *Ramicrusta* sp., while settlement to living surface was reduced to 8 CCA treatment chips in November (Figure S5 and S6). *L.* cf. *pygmaeum* which induced settlement to its living surface of 56.3 ± 6.5% in October (highest amongst all CCA species tested), did not induce any settlement (0%) to living surface in November. A reduction in settlement to living surface of CCAs was broadly seen across all other CCA treatments from October to November, ranging from 33–100% reduction. Out of all CCAs, *Titanoderma* cf. *tessellatum* had one of the lowest reductions in settlement to living surface from 51.2 ± 7.1% in October to 32.8 ± 6.5% in November (36% reduction).

Coral species-specific settlement behaviours to CCAs

ACROPORIDAE

Acropora tenuis

Settlement behaviours of *A. tenuis* larvae were investigated in both October and November 2021 to assess if there was a temporal effect on the settlement inducive properties of CCAs that had been maintained in aquaria between spawning periods. Zero settlement in blank treatment wells across both time periods indicates that larvae from both batches were still healthy at the end of the assay (Figure S5 and S6), with all larvae exhibiting normal swimming behaviours; this applies to all subsequent assays performed on other coral larval species.

The inducive properties of CCAs were similar between the two time periods with median larval settlement exceeding 75% for *T.* cf. *tessellatum*, *L.* cf. *kotschyanum*, *L.* cf. *insipidum*, *A.* cf. *boscensei*, *H.* cf. *reinboldii*, *L.* cf. *pygmaeum*, *P.* cf. *onkodes* "Orange", *P.* cf. *onkodes* "Yellow conceptacles", *P.* cf. *onkodes* "Chalky", *Sporolithon* sp., and coral rubble (Figure S5A and S6A). Larval settlement to some of the CCA treatments that had low (<50%) to moderate (between 50 to 75%) settlement in October were found to be higher in November, reaching >75% settlement that include *M.* cf. *madagascarense* (1.1× higher mean settlement), *N.* cf. *fosliei* (1.5×), *L.* cf. *proliferum* (1.1x), and *Ramicrusta* sp. (1.3×; Table 3 of main article), and is likely due to larvae (1 day older) in November being less selective as shown by higher settlement in the sterile aragonite treatment (substrate control), with 13.6 ± 4.8% mean settlement recorded in October compared to 50 ± 10.9% settlement in November. In both months, settlement to *A.* cf. *foliacea* remained low (<20%) indicating that this CCA species is not a good inducer for settlement for *A. tenuis* larvae.

There was a significant effect of CCA treatments on larval settlement in both October and November 2021 (Kruskal-Wallis, $p < 0.001$; Figure S7). In October, most CCA species significantly induced higher levels of settlement when compared to the aragonite (substrate) control (Dunn's test, $p < 0.05$; Figure S7), except for *A.* cf. *foliacea*, *M.* cf. *madagascarense*, *N.* cf. *fosliei*, *L.* cf. *proliferum*, *Sporolithon* sp., *Ramicrusta* sp. and coral rubble. In November, the differentiation between CCA and aragonite treatments was less prominent with only *Ramicrusta* sp., *A.* cf. *bosencei* and the coral rubble treatment inducing higher settlement than the aragonite control (Dunn's test, $p < 0.05$). Of note, all CCA species tested except for *A.* cf. *foliacea* induced similar levels of settlement in both

months when compared to the coral rubble treatment (Figure S5A and S6A), suggesting *A. tenuis* to be a generalist that would settle in the presence of most common species of CCA species.

A. tenuis larvae settled onto the living surface of most (13) CCA treatment chips in October except for *L.* cf. *proliferum*, *N.* cf. *fosliei* and *Ramicrusta* sp., while settlement to living surface was reduced to 8 CCA treatment chips in November (Figure S5B and S6B). *L.* cf. *pygmaeum* which induced settlement to its living surface of 56.3 ± 6.5% in October (highest amongst CCA species tested), did not induce any settlement (0%) to living surface in November; a reduction in settlement to living surface of CCAs was broadly seen across all other treatments from October to November. Out of all CCAs, *Titanoderma* cf. *tessellatum* had one of the lowest reductions in settlement to living surface from 51.2 ± 7.1% in October to 32.8 ± 6.5% in November (36% reduction; Figure S5B and S6B).

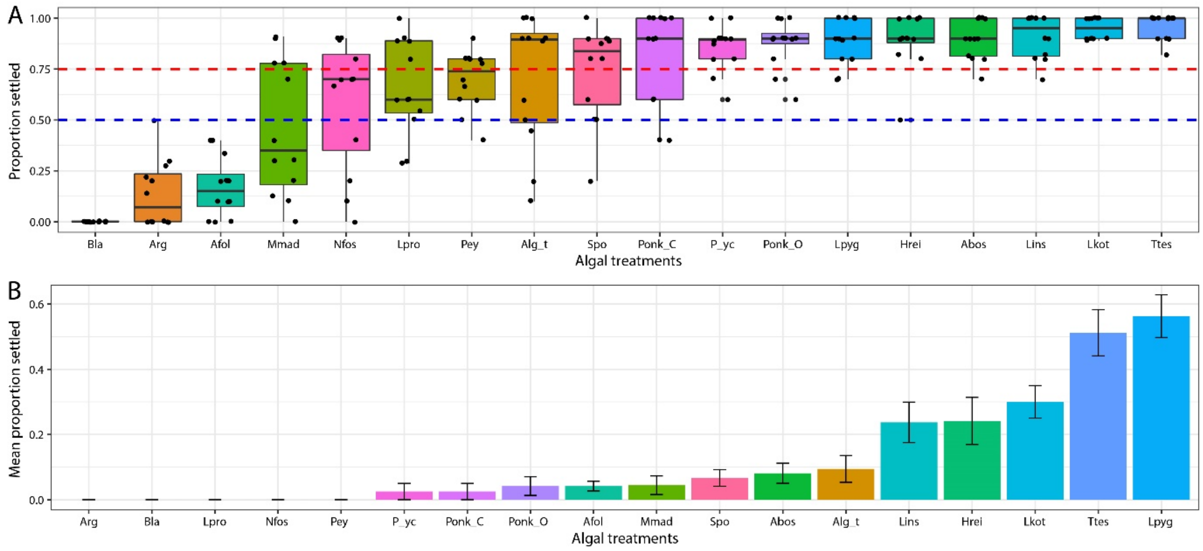

Figure S5: *Acropora tenuis*. October. Larval settlement responses as proportion of total larvae counted at the end of the experiment that had successfully settled to the algal treatments, as A) box plots of total settlement and B) mean settlement (± SE) to the live surface of CCA chips. Responses to algal treatments are ordered in ascending order from left to right based on mean proportion settled. The horizontal blue dashed line on boxplots represents the 50% settlement threshold and horizontal red dashed line represents the 75% settlement threshold. Black dots represent data points for assay replicates within each algal treatment. Alg_t refers to the coral rubble treatment and Pey refers to the *Ramicrusta* sp. treatment.

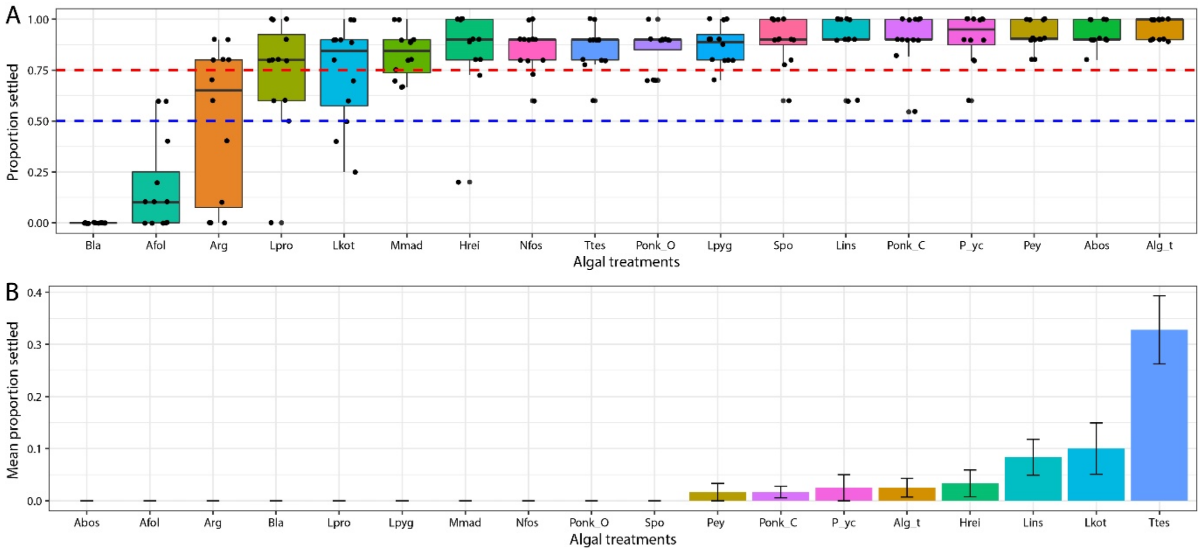

Figure S6: *Acropora tenuis*. November. Larval settlement responses as proportion of total larvae counted at the end of the experiment that had successfully settled to the algal treatments, as A) box plots of total settlement and B) mean settlement (± SE) to the live surface of CCA chips. Responses to algal treatments are ordered in ascending order from left to right based on mean proportion settled. The horizontal blue dashed line on boxplots represents the 50% settlement threshold and horizontal red dashed line represents the 75% settlement threshold. Black dots represent data points for assay replicates within each algal treatment. Alg_t refers to the coral rubble treatment and Pey refers to the *Ramicrusta* sp. treatment.

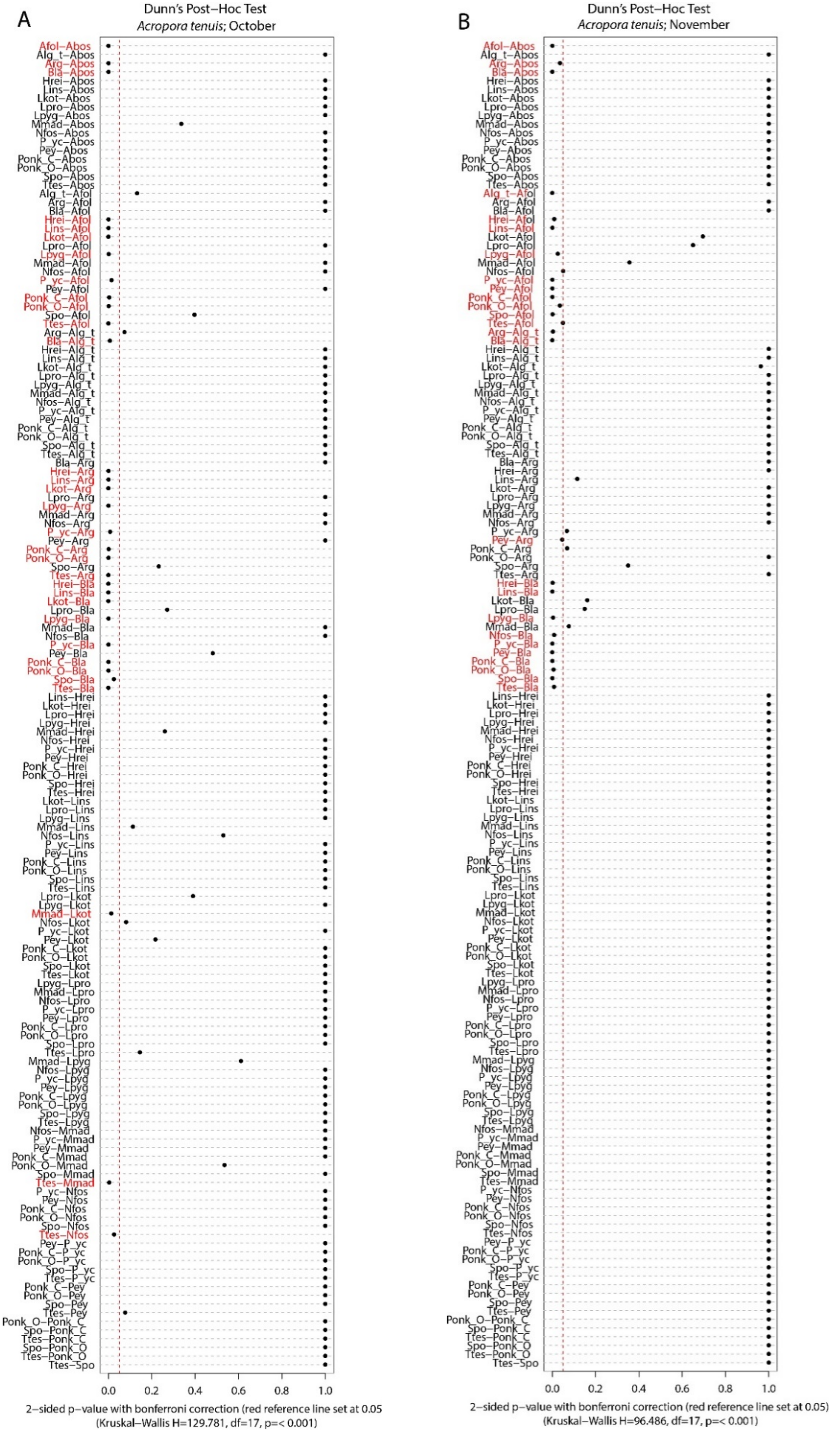

Figure S7: *Acropora tenuis.* Dunn's pairwise tests with Bonferroni correction for assays performed in A) October 2021 and B) November 2021. The red vertical dashed lines indicate p-value of 0.05, with tests (dots) falling to the left the line showing significant tests. Alg_t refers to the coral rubble treatment and Pey refers to the *Ramicrusta* sp. treatment.

Table S2: Mean settlement onto living surface, as a percentage of total settled, of CCA treatment chips (%; SE in parentheses) of coral larvae from 15 species across 5 taxonomic families when presented with 15 common encrusting red algal species across 8 taxonomic families from the Great Barrier Reef. Coral and CCA abbreviations are provided in parentheses. Cells with warmer colours represent higher settlement values. Refer to Table 1 of main article for details on coral larval species

**Family**	Acroporidae	Acroporidae	Acroporidae	Acroporidae	Merulinidae	Merulinidae	Merulinidae	Merulinidae	Merulinidae	Merulinidae	Merulinidae	Lobophylliidae	Lobophylliidae	Poritidae	Fungiidae
**Species**<br>**CCA treatments**	*Acropora tenuis* **(Aten)**	*Acropora anthocersis* **(Aant)**	*Acropora hyacinthus* **(Ahya)**	*Montipora aequituberculata* **(Maeq)**	*Coelastrea aspera* **(Casp)**	*Caulastrea furcata* **(Cfur)**	*Dipsastrea favus* **(Dfav)**	*Goniastrea favulus* **(Gfav)**	*Mycedium elephantotus* **(Mele)**	*Platygyra sinensis* **(Psin)**	*Platygyra daedalea* **(Pdae)**	*Echinophyllia aspera* **(Easp)**	*Lobophyllia corymbosa* **(Lcor)**	*Porites lobata* **(Plob)**	*Fungia fungites* **(Ffun)**
Blank **(Bla)**	0.0 (0)	0.0 (0)	0.0 (0)	0.0 (0)	0.0 (0)	0.0 (0)	0.0 (0)	0.0 (0)	0.0 (0)	0.0 (0)	0.0 (0)	0.0 (0)	0.0 (0)	0.0 (0)	0.0 (0)
Sterile aragonite chips **(Arg)**	0.0 (0)	0.0 (0)	0.0 (0)	0.0 (0)	0.0 (0)	0.0 (0)	0.0 (0)	0.0 (0)	0.0 (0)	0.0 (0)	0.0 (0)	0.0 (0)	0.0 (0)	0.0 (0)	0.0 (0)
Coral rubble **(Rub)**	19.6 (8.9)	2.8 (2.8)	1.0 (1.0)	6.9 (4.8)	0.0 (0)	7.4 (7.4)	5.8 (3.1)	0.8 (0.8)	0.0 (0)	2.1 (2.1)	0.0 (0)	0.9 (0.9)	0.0 (0)	28.2 (10.0)	0.0 (0)
Ramicrusta sp. **(Ram)**	0.0 (0)	0.0 (0)	1.7 (1.7)	0.0 (0)	0.0 (0)	0.0 (0)	0.0 (0)	0.0 (0)	0.0 (0)	0.0 (0)	0.0 (0)	0.0 (0)	0.0 (0)	0.0 (0)	0.0 (0)
Lithothamnion proliferum **(Lpro)**	0.0 (0)	0.0 (0)	0.0 (0)	0.0 (0)	0.0 (0)	0.0 (0)	0.0 (0)	0.0 (0)	0.0 (0)	0.0 (0)	0.0 (0)	0.0 (0)	0.0 (0)	0.0 (0)	0.0 (0)
Melyvonnea cf. *madagascariensis* **(Mmad)**	7.3 (4.3)	14.4 (5.0)	21.1 (11.1)	0.0 (0)	0.0 (0)	0.0 (0)	0.0 (0)	1.4 (1.4)	0.0 (0)	0.0 (0)	0.0 (0)	0.0 (0)	1.2 (1.2)	2.6 (1.8)	0.0 (0)
Adeylithon bosencei **(Abos)**	9.6 (3.7)	6.8 (3.3)	0.0 (0)	0.0 (0)	0.0 (0)	0.0 (0)	0.0 (0)	0.0 (0)	0.0 (0)	0.0 (0)	0.0 (0)	0.0 (0)	0.0 (0)	13.3 (6.2)	0.0 (0)
Hydrolithon reinboldii **(Hrei)**	25.5 (7.4)	5.4 (3.5)	1.2 (1.2)	2.4 (1.7)	0.0 (0)	0.0 (0)	0.0 (0)	3.3 (2.2)	0.0 (0)	0.0 (0)	0.0 (0)	0.0 (0)	0.0 (0)	6.6 (2.9)	0.0 (0)
Amphiroa foliacea **(Afol)**	25.0 (11.1)	28.5 (10.6)	0.0 (0)	0.0 (0)	0.0 (0)	0.0 (0)	8.3 (8.3)	0.0 (0)	0.0 (0)	8.3 (8.3)	0.0 (0)	0.0 (0)	0.0 (0)	4.2 (3.0)	0.0 (0)
Lithophyllum insipidum **(Lins)**	25.9 (6.9)	6.9 (3.6)	16.3 (5.7)	1.0 (1.0)	0.0 (0)	4.8 (4.8)	0.0 (0)	0.0 (0)	0.0 (0)	0.0 (0)	0.0 (0)	0.0 (0)	0.0 (0)	0.9 (0.9)	0.0 (0)
Lithophyllum kotchyanum **(Lkot)**	31.6 (5.2)	8.3 (3.0)	5.4 (3.5)	2.3 (1.6)	0.0 (0)	0.0 (0)	0.0 (0)	1.7 (1.7)	0.0 (0)	0.0 (0)	0.0 (0)	0.8 (0.8)	0.9 (0.9)	1.9 (1.9)	0.0 (0)
Lithophyllum pygmaeum **(Lpyg)**	64.9 (7.5)	22.2 (7.5)	0.0 (0)	0.0 (0)	0.0 (0)	0.0 (0)	7.2 (5.7)	12.5 (9.0)	0.0 (0)	8.3 (8.3)	0.0 (0)	30.3 (10.2)	0.0 (0)	4.3 (2.4)	0.0 (0)
Titanoderma tessellatum **(Ttes)**	53.6 (7.4)	13.9 (3.4)	29.0 (5.3)	3.7 (2.8)	0.0 (0)	0.0 (0)	0.0 (0)	0.0 (0)	0.0 (0)	0.0 (0)	0.0 (0)	0.0 (0)	0.0 (0)	51.3 (8.8)	0.0 (0)
Neogoniolithon fosliei **(Nfos)**	0.0 (0)	0.0 (0)	0.0 (0)	0.0 (0)	0.0 (0)	0.0 (0)	0.0 (0)	0.0 (0)	0.0 (0)	0.0 (0)	0.0 (0)	0.0 (0)	0.0 (0)	0.0 (0)	0.0 (0)
Porolithon cf. *onkodes* "Orange" **(Ponk_O)**	4.6 (3.2)	15.1 (7.4)	8.3 (5.6)	0.0 (0)	0.0 (0)	0.0 (0)	0.0 (0)	0.0 (0)	0.0 (0)	0.0 (0)	0.0 (0)	0.0 (0)	0.0 (0)	16.9 (8.1)	0.0 (0)
Porolithon cf. *onkodes* "Chalky" **(Ponk_C)**	6.3 (6.3)	0.9 (0.9)	2.6 (1.7)	0.0 (0)	0.0 (0)	33.3 (33.3)	0.0 (0)	0.0 (0)	0.0 (0)	0.0 (0)	0.0 (0)	0.0 (0)	0.0 (0)	1.8 (1.2)	0.0 (0)
Porolithon sp. "Yellow conceptacles" **(P_yc)**	2.8 (2.8)	3.5 (2.4)	15.1 (6.6)	0.0 (0)	0.0 (0)	4.2 (4.2)	0.0 (0)	0.0 (0)	0.0 (0)	0.0 (0)	0.0 (0)	3.6 (3.6)	0.0 (0)	29.4 (11.5)	0.0 (0)
Sporolithon sp. **(Spo)**	7.8 (3.0)	0.0 (0)	0.0 (0)	0.0 (0)	0.0 (0)	0.0 (0)	0.0 (0)	0.0 (0)	0.0 (0)	0.0 (0)	0.0 (0)	0.0 (0)	0.0 (0)	0.0 (0)	0.0 (0)

Acropora anthocercis

There was a significant effect of CCA treatment on settlement (Kruskal-Wallis, h = 115.67, df = 17, $p < 0.001$). Unlike all the other coral species assessed in the study, low–moderate levels of mean settlement (mean 40 ± 5.9%) was recorded for *A. anthocercis* in the blank treatment, indicating that there were potential sub-optimal health conditions of larvae that were raised in culture that resulted in this indiscriminate settlement behaviours (Figure S8A). This could have been due to the early release of gametes, 4–6 days ahead of its predicted spawning in the wild (Table 1 in main article; reported as *A. hyacinthus* in Willis et al. 1985, likely from handling stress. As such, the absolute value of settlement across treatments, and thus inter-specific comparisons, must be treated with caution, with the settlement level in blank treatments serving as baseline for all other treatments within this species.

Pairwise tests showed no difference in larval settlement in most of the CCA treatments to the blank, except for *L.* cf. *insipidum*, *L.* cf. *kotschyanum*, *P.* cf. *onkodes* "chalky" and *T.* cf. *tessellatum*, which all induced significantly higher levels of settlement (Dunn's test, $p < 0.05$; Figure S9). Similarly, all of the aforementioned CCA species also induced higher levels of settlement compared to the aragonite control (Dunn's test, $p < 0.05$; Figure S9). The coral rubble treatment induced moderate mean settlement of 72.5 ± 7.5 % (Figure S8A), with the highest settlement found in *L.* cf. *insipidum* (94.2 ± 2.3%), followed by *L.* cf. *kotschyanum* (91.8 ± 2.7%) and *T.* cf. *tessellatum* (89.0 ± 2.6%), however settlement in these latter 3 CCA were not significantly different to that found in the coral rubble treatment (Table 3 in main article; Dunn's test, $p > 0.05$, Figure S9). The lowest settlement was found in the *L.* cf. *proliferum* treatment (31.8 ± 4.6%).

A. anthocercis larvae settled onto the living surface of most of (12) the CCA treatment chips except *L.* cf. *proliferum*, *N.* cf. *fosliei*, *Ramicrusta* sp. and *Sporolithon* sp. (Figure S8B). Highest level of settlement to living surface was found in *L.* cf. *pygmaeum* (15.2 ± 4.5%), followed by *T.* cf. *tessellatum* (12.3 ± 3.1%) and *P.* cf. *onkodes* "Orange" (10.6 ± 4.7%).

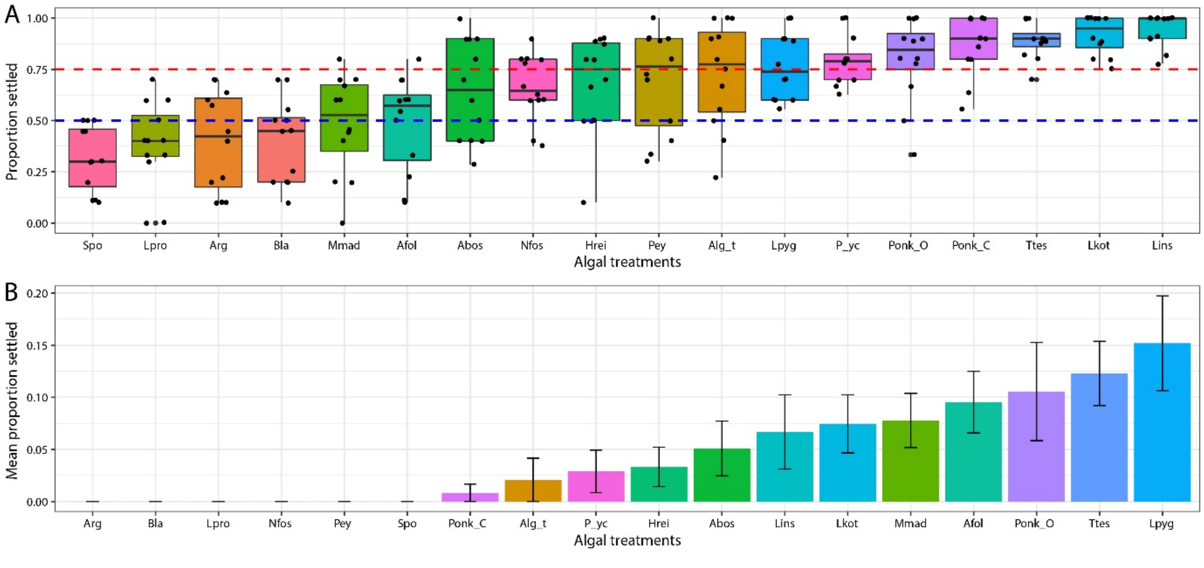

Figure S8: *Acropora anthocercis*. October. Larval settlement responses as proportion of total larvae counted at the end of the experiment that had successfully settled to the algal treatments, as A) box plots of total settlement and B) mean settlement (± SE) to the live surface of CCA chips. Responses to algal treatments are ordered in ascending order from left to right based on mean proportion settled. The horizontal blue dashed line on boxplots represents the 50% settlement threshold and horizontal red dashed line represents the 75% settlement threshold. Black dots represent data points for assay replicates within each algal treatment. Alg_t refers to the coral rubble treatment and Pey refers to the *Ramicrusta* sp. treatment.

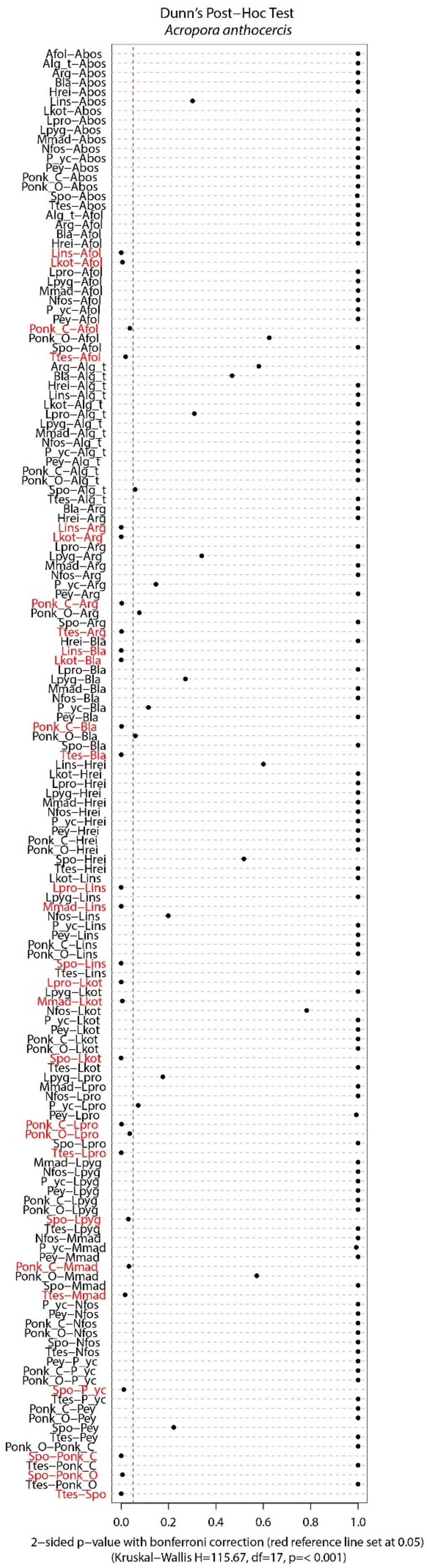

Figure S9: *Acropora anthocercis.* Dunn's pairwise tests with Bonferroni correction. The red vertical dashed line indicates p-value of 0.05, with tests (dots) falling to the left the line showing significant tests. Alg_t refers to the coral rubble treatment and Pey refers to the *Ramicrusta* sp. treatment.

Acropora hyacinthus

There was a significant effect of CCA treatments on larval settlement (Kruskal-Wallis: H = 136.004, p < 0.001) and zero settlement was found in the blank treatment and low level of settlement in the aragonite substrate control (8.0 ± 4.2%) for *A. hyacinthus* larvae (Table 3 of main article, Figure S10A). *P.* cf. *onkodes* "chalky", coral rubble, *P.* cf. *onkodes* "yellow conceptacle"*, L.* cf. *pygmaeum, T.* cf. *tessellatum* and *L.* cf. *insipidum* induced significantly higher settlement than the aragonite control (Dunn's test p < 0.05; Figure S11). High level of settlement (>75%) was found in the *L.* cf. *insipidum* (82.8 ± 3.2%), *T.* cf. *tessellatum* (80.6 ± 1.5%), *L.* cf. *pygmaeum* (78.9 ± 4.5%) treatments, however, were not statistically different to settlement levels found in the in the coral rubble treatment (68.8 ± 7.5%; Figure S11). While *A. tenuis* settled at high levels to the majority of CCA tested (i.e. 9 out of 15, and 12 out of 15 CCA species in October and November respectively), *A. hyacinthus* larvae are comparatively more selective with only 3 out of 15 CCA species inducing >75% settlement.

A. hyacinthus settled onto the living surfaces of most (10) CCA treatment chips except for *A.* cf. *boscensei*, *L.* cf. *proliferum*, *L.* cf. *pygmaeum*, *N.* cf. *fosliei* and *Sporolithon* sp. (Figure S10B). *Titanoderma* cf. *tessellatum* induced the highest settlement to living surface (23.7 ± 4.3%), followed by *L.* cf. *insipidum* (12.8 ± 4.2%) and *P.* cf. *onkodes* "Yellow conceptacles" (10.8 ± 4.8%).

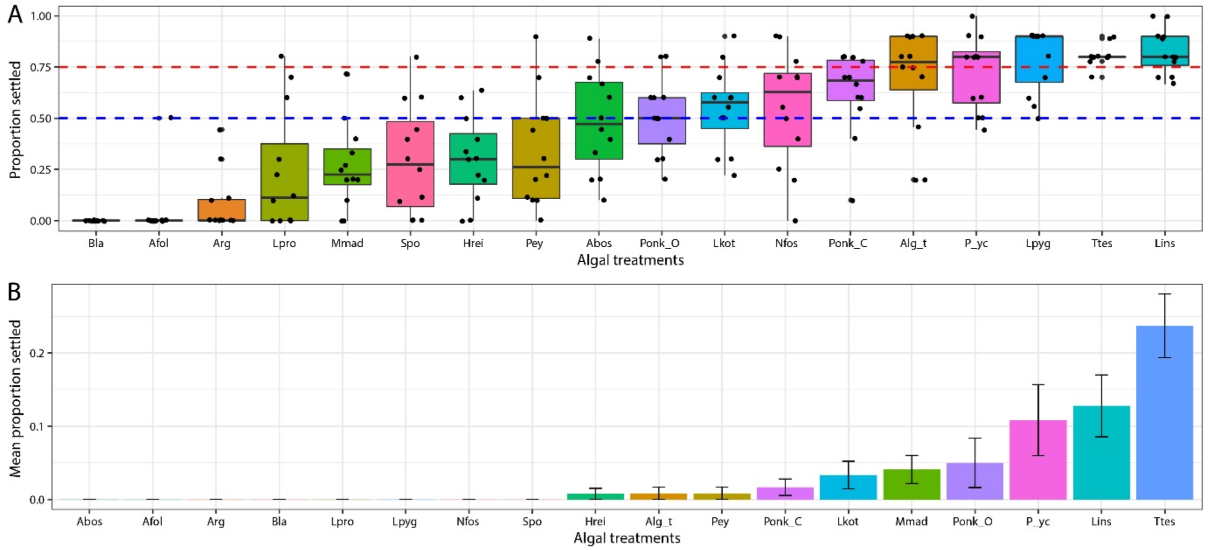

Figure S10: *Acropora hyacinthus*. November. Larval settlement responses as proportion of total larvae counted at the end of the experiment that had successfully settled to the algal treatments, as A) box plots of total settlement and B) mean settlement (± SE) to the live surface of CCA chips. Responses to algal treatments are ordered in ascending order from left to right based on mean proportion settled. The horizontal blue dashed line on boxplots represents the 50% settlement threshold and horizontal red dashed line represents the 75% settlement threshold. Black dots represent data points for assay replicates within each algal treatment. Alg_t refers to the coral rubble treatment and Pey refers to the *Ramicrusta* sp. treatment.

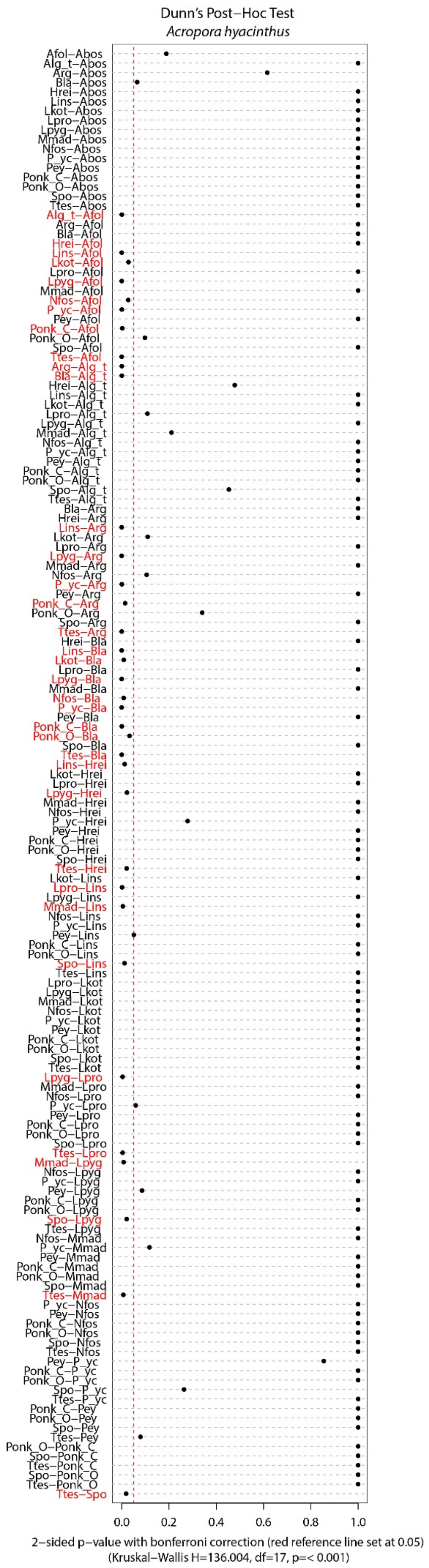

Figure S11: *Acropora hyacinthus.* Dunn's pairwise tests with Bonferroni correction. The red vertical dashed line indicates p-value of 0.05, with tests (dots) falling to the left the line showing significant tests. Alg_t refers to the coral rubble treatment and Pey refers to the *Ramicrusta* sp. treatment.

Montipora aequituberculata

There was a significant effect of CCA treatments on *M. aequituberculata* settlement (Kruskal-Wallis: H = 116.26, df = 17, p < 0.001) and no settlement was detected in both the blank and aragonite treatments in *M. aequituberculata* (Table 3 in main article, Figure S12A). Mean settlement was low in majority of the CCA treatment tested, with 9 out 15 CCA treatments inducing <50% settlement, along with the coral rubble treatment (23.3 ± 9.1%). *Amphiroa* cf. *foliacea* induced zero settlement in this species. *Hydrolithon* cf. *reinboldii*, *L.* cf. *insipidum*, *L.* cf. *kotschyanum*, *M.* cf. *madagascariensis*, *P.* cf. *onkodes* "Yellow conceptacles" and *T.* cf. *tessellatum* all induced significantly higher settlement compared to the aragonite control (Dunn's test, p < 0.05, Figure S13). Highest settlement was found in the *L.* cf. *kotschyanum* (79.2 ± 4.2%) treatment, followed by *L.* cf. *insipidum* (74.8 ± 6.0%) and *M.* cf. *madagascariensis* (72.9 ± 6.1%), although these were not significantly higher when compared to settlement in the coral rubble treatment (Dunn's test, p > 0.05; Figure S13). While median settlement was high in *T.* cf. *tessellatum* (>75%), high variability was found in this treatment resulting in mean settlement of 59.7 ± 11.4% (Figure S12A). *Lithophyllum* cf. *pygmaeum*, which was a good inducer in other species tested in the family Acroporidae (i.e. >75% settlement in *Acropora* spp.), performed poorly in this species (3.3 ± 1.4%).

M. aequituberculata larvae settled on to the living surface of only *L.* cf. *insipidum*, *H.* cf. *reinboldii*, *L.* cf. *kotschyanum*, *T.* cf. *tessellatum* and the coral rubble chips (Figure S12B). Settlement to living surface was typically low not exceeding 5%, with *T.* cf. *tessellatum* inducing the highest settlement of 3.1 ± 2.3%.

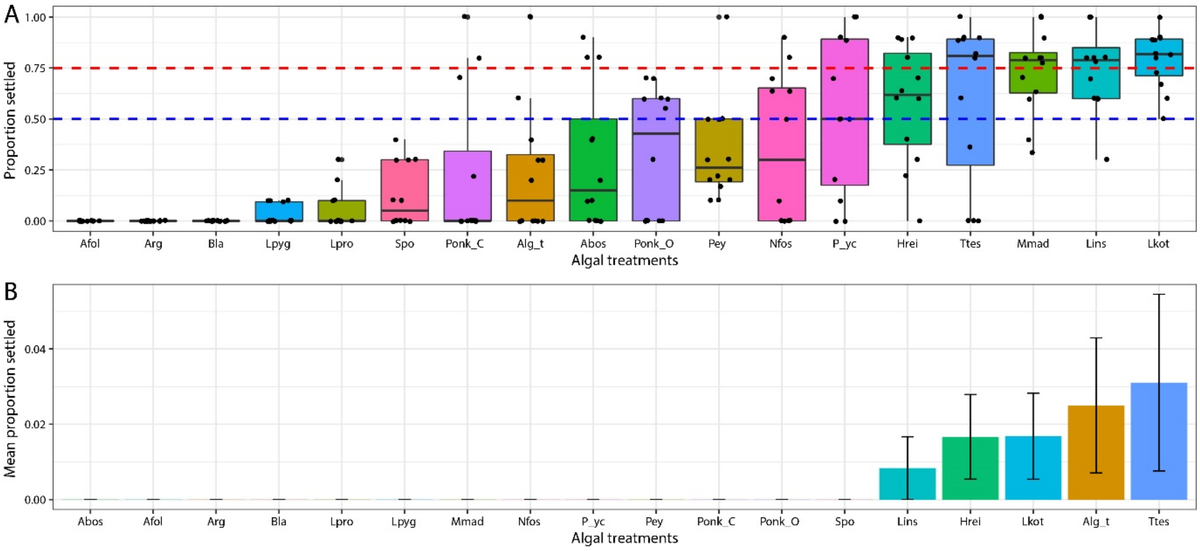

Figure S12: *Montipora aequituberculata*. October. Larval settlement responses as proportion of total larvae counted at the end of the experiment that had successfully settled to the algal treatments, as A) box plots of total settlement and B) mean settlement (± SE) to the live surface of CCA chips. Responses to algal treatments are ordered in ascending order from left to right based on mean proportion settled. The horizontal blue dashed line on boxplots represents the 50% settlement threshold and horizontal red dashed line represents the 75% settlement threshold. Black dots represent data points for assay replicates within each algal treatment. Alg_t refers to the coral rubble treatment and Pey refers to the *Ramicrusta* sp. treatment.

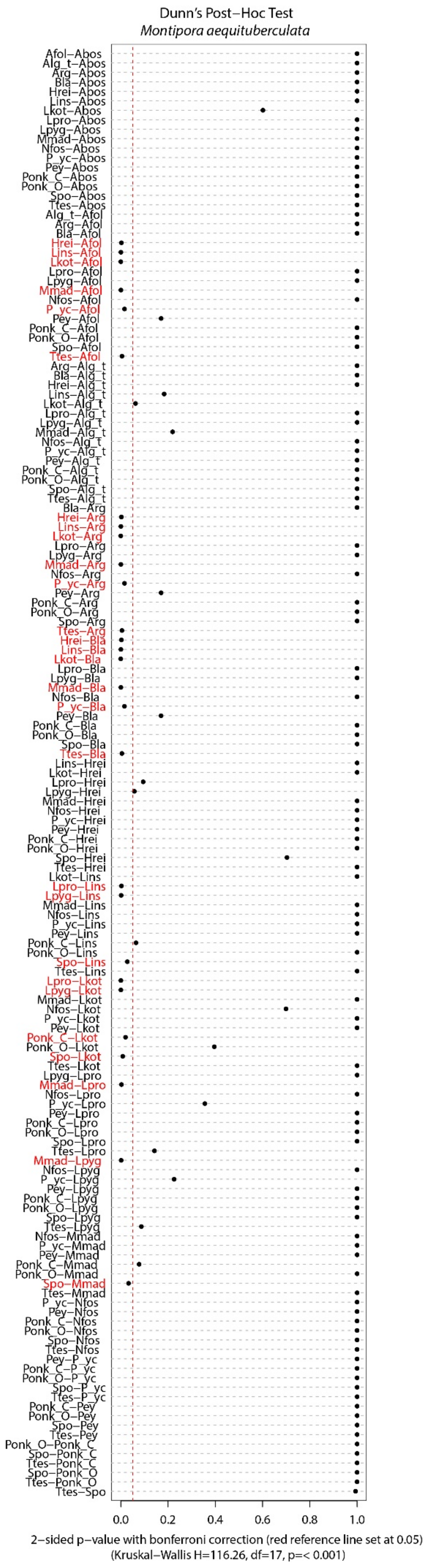

Figure S13: *Montipora aequituberculata.* Dunn's pairwise tests with Bonferroni correction. The red vertical dashed line indicates p-value of 0.05, with tests (dots) falling to the left the line showing significant tests. Alg_t refers to the coral rubble treatment and Pey refers to the *Ramicrusta* sp. treatment.

MERULINIDAE

Coelastrea aspera

Larvae at three ages were tested for *C. aspera*, namely 5-day, 8-day and 18-day old at the start of assays, to assess for changes in settlement competency and CCA preferences with time. There was no settlement in the blank and aragonite treatments in 5-day and 8-day old larvae with low levels of settlement (1.7 ± 1.7%) detected in 18-day old larvae in the aragonite treatment (Figure S14A). There was a significant effect of CCA treatments on 5-day old *C. aspera* (Kruskal-Wallis: H = 71.74, df =17, p < 0.001), with all treatments inducing <32% settlement, and 7 out of the 15 CCAs tested inducing <5% settlement indicating likelihood that competency for settlement has not yet been reached (Figure S14A). Mean settlement in the coral rubble treatment was 18.9 ± 5.5% with the highest settlement found in the *N.* cf. *fosliei* treatment at 31.0 ± 12.0% (Figure S14A). Only the coral rubble treatment induced significantly higher settlement compared to the aragonite control (Dunn's test <0.05, Figure S15), with low levels of settlement to the living surface of the coral rubble chip at 3.4 ± 1.9% (Figure S14B).

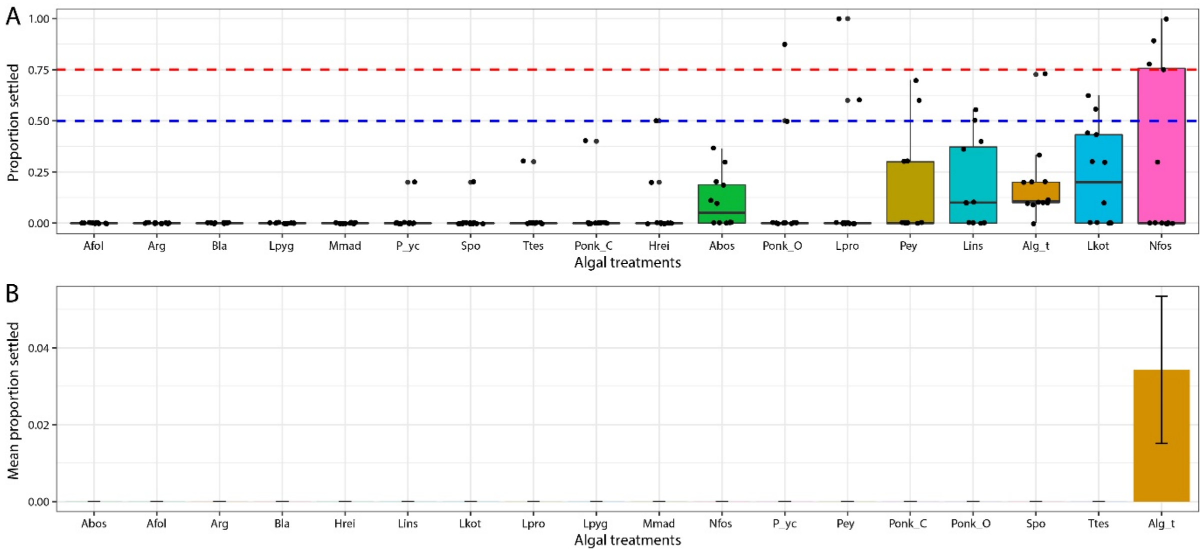

Figure S14: *Coelastrea aspera*. 5-day old larvae. October. Larval settlement responses as proportion of total larvae counted at the end of the experiment that had successfully settled to the algal treatments, as A) box plots of total settlement and B) mean settlement (± SE) to the live surface of CCA chips. Responses to algal treatments are ordered in ascending order from left to right based on mean proportion settled. The horizontal blue dashed line on boxplots represents the 50% settlement threshold and horizontal red dashed line represents the 75% settlement threshold. Black dots represent data points for assay replicates within each algal treatment. Alg_t refers to the coral rubble treatment and Pey refers to the *Ramicrusta* sp. treatment.

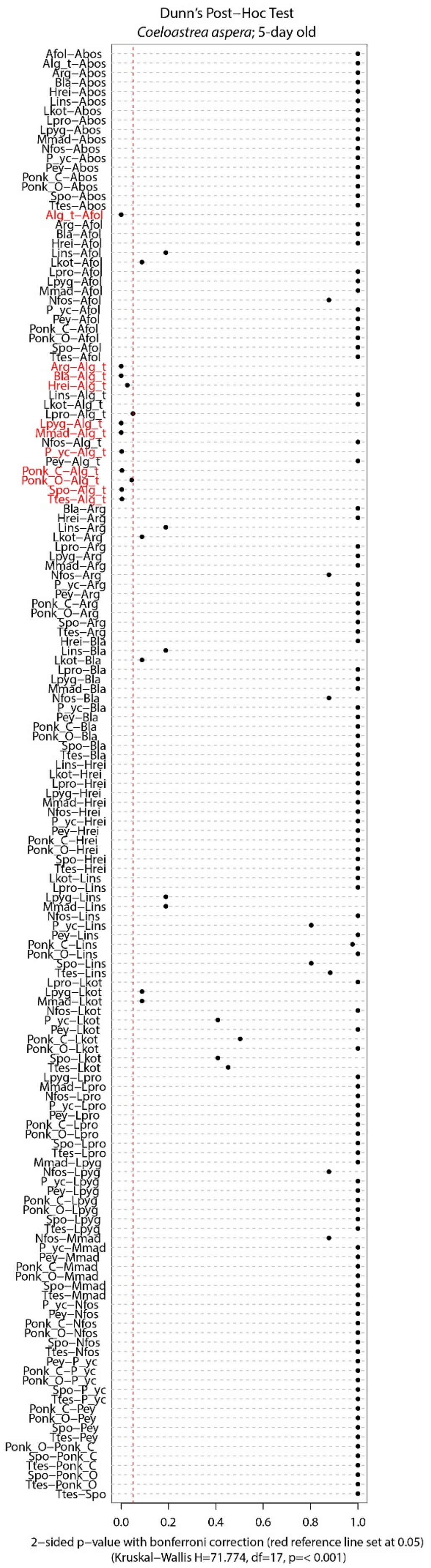

Figure S15: *Coeloastrea aspera.* 5-day old. Dunn's pairwise tests with Bonferroni correction. The red vertical dashed line indicates p-value of 0.05, with tests (dots) falling to the left the line showing significant tests. Alg_t refers to the coral rubble treatment and Pey refers to the *Ramicrusta* sp. treatment.

There was a significant effect of CCA treatments on larval settlement in 8-day and 18-day old *C. aspera* larvae (Kruskal-Wallis: 8-day old H = 65.625, df = 17. $p < 0.001$; 18-day old H = 63.749, df = 17, $p < 0.001$; Figure S17 and S19). Mean settlement in coral rubble treatment tripled, with 8-day day and 18-day old larvae inducing similar levels settlement of 58.0 ± 13.4% and 54.1 ± 14.7% respectively, suggesting an improvement in settlement competencies in older *C. aspera* beyond 5-days of age. While there were slight differences in the preferential order of settlement to CCA between 8-day and 18-day old larvae, the trends were in general similar, with *L.* cf. *insipidum* and *N.* cf. *fosliei* being the best inducer of settlement in *C. aspera*, with *L.* cf. *insipidum* inducing the highest settlement in 8-day old larvae (72.8 ± 5.4%) and the *N.* cf. *fosliei* treatment in 18-day old larvae (71.7 ± 11.7%; Figure S16A and S18A). In both cases, the CCA treatments induced significantly higher settlement compared to the aragonite control (Dunn's test, $p < 0.05$; Figure S19). Mean settlement in the *L.* cf. *insipidum* and *N.* cf. *fosliei* treatments were not significantly different to that for the coral rubble treatment (Dunn's test, $p > 0.05$; Figure S17 and S19). CCA species that were good inducers in acroporid species including *T.* cf. *tessellatum* and *L.* cf. *pygmaeum* were ineffective for *C. aspera*, scoring a maximum mean settlement of 53.3 ± 11.7% and 0% respectively across larval ages. *Amphiroa* cf. *foliacea* did not induce settlement in *C. aspera*.

There was no settlement to the living surface of CCA treatment chips in 8-day old *C. aspera* larvae, and similar levels of settlement in 18-day old larvae to living surface of the coral rubble chips found in 5-day old larval assays (3.3 ± 2.1%; Figure S16B and S17B).

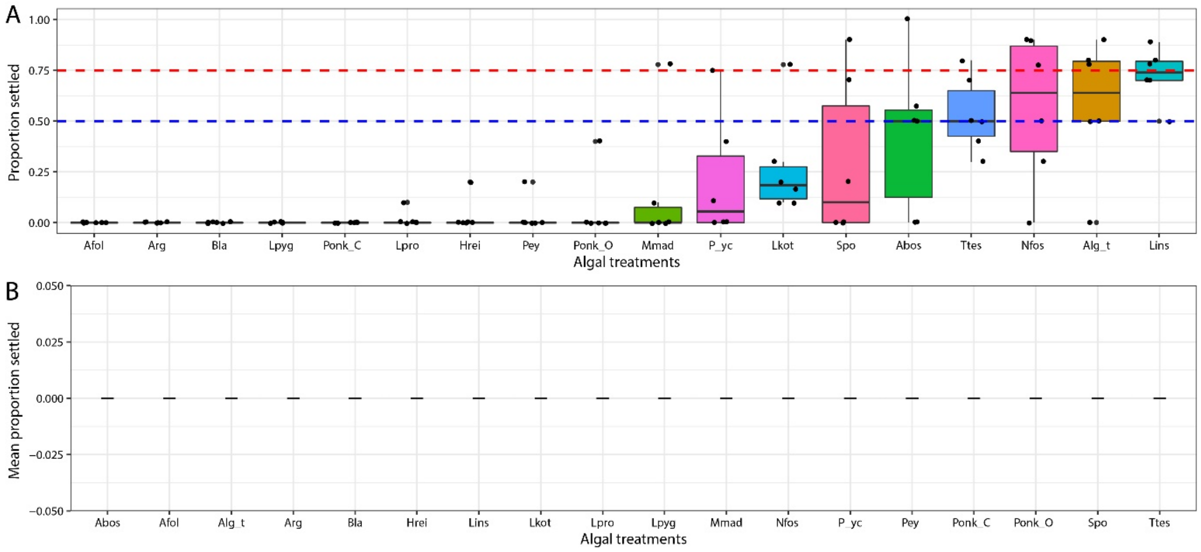

Figure S16: *Coelastrea aspera*. 8-day old larvae. October. Larval settlement responses as proportion of total larvae counted at the end of the experiment that had successfully settled to the algal treatments, as A) box plots of total settlement and B) mean settlement (± SE) to the live surface of CCA chips. Responses to algal treatments are ordered in ascending order from left to right based on mean proportion settled. The horizontal blue dashed line on boxplots represents the 50% settlement threshold and horizontal red dashed line represents the 75% settlement threshold. Black dots represent data points for assay replicates within each algal treatment. Alg_t refers to the coral rubble treatment and Pey refers to the *Ramicrusta* sp. treatment.

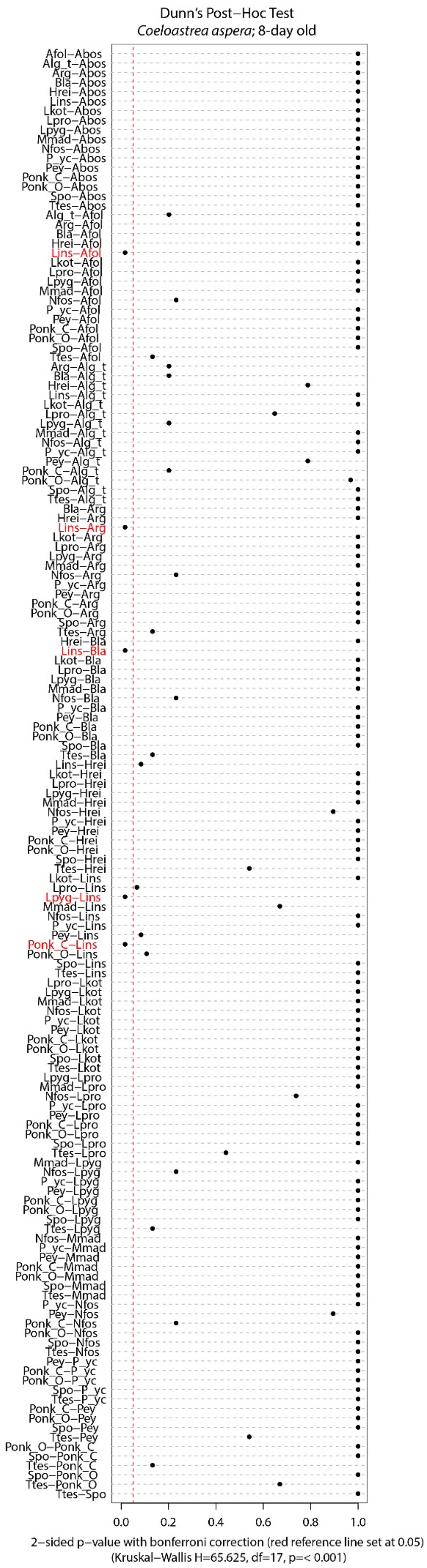

Figure S17: *Coeloastrea aspera.* 8-day old. Dunn's pairwise tests with Bonferroni correction. The red vertical dashed line indicates p-value of 0.05, with tests (dots) falling to the left the line showing significant tests. Alg_t refers to the coral rubble treatment and Pey refers to the *Ramicrusta* sp. treatment.

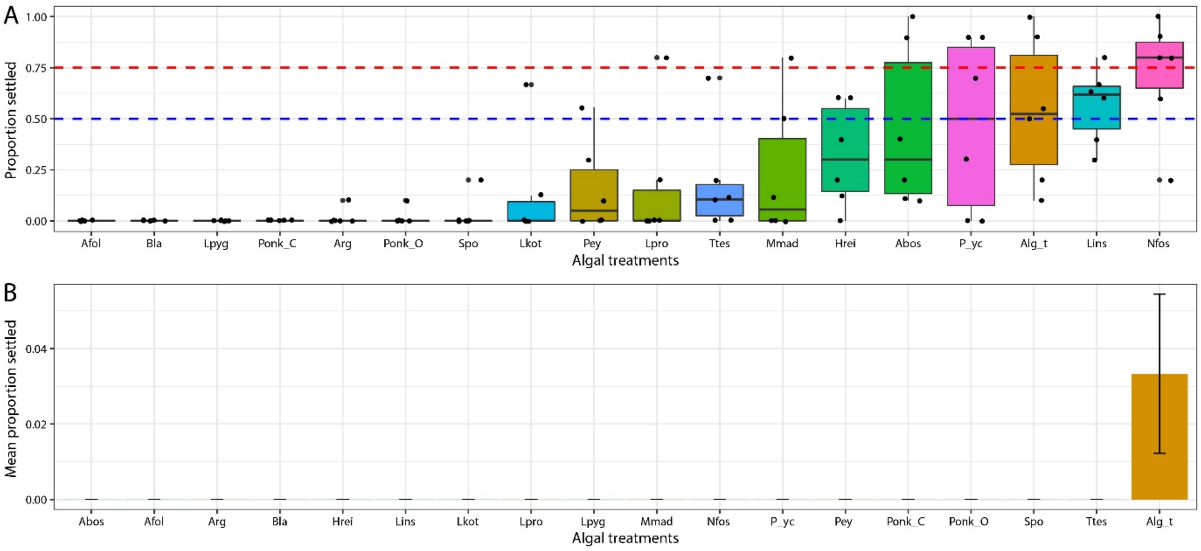

Figure S18: *Coelastrea aspera*. 18-day old larvae. October. Larval settlement responses as proportion of total larvae counted at the end of the experiment that had successfully settled to the algal treatments, as A) box plots of total settlement and B) mean settlement (± SE) to the live surface of CCA chips. Responses to algal treatments are ordered in ascending order from left to right based on mean proportion settled. The horizontal blue dashed line on boxplots represents the 50% settlement threshold and horizontal red dashed line represents the 75% settlement threshold. Black dots represent data points for assay replicates within each algal treatment. Alg_t refers to the coral rubble treatment and Pey refers to the *Ramicrusta* sp. treatment.

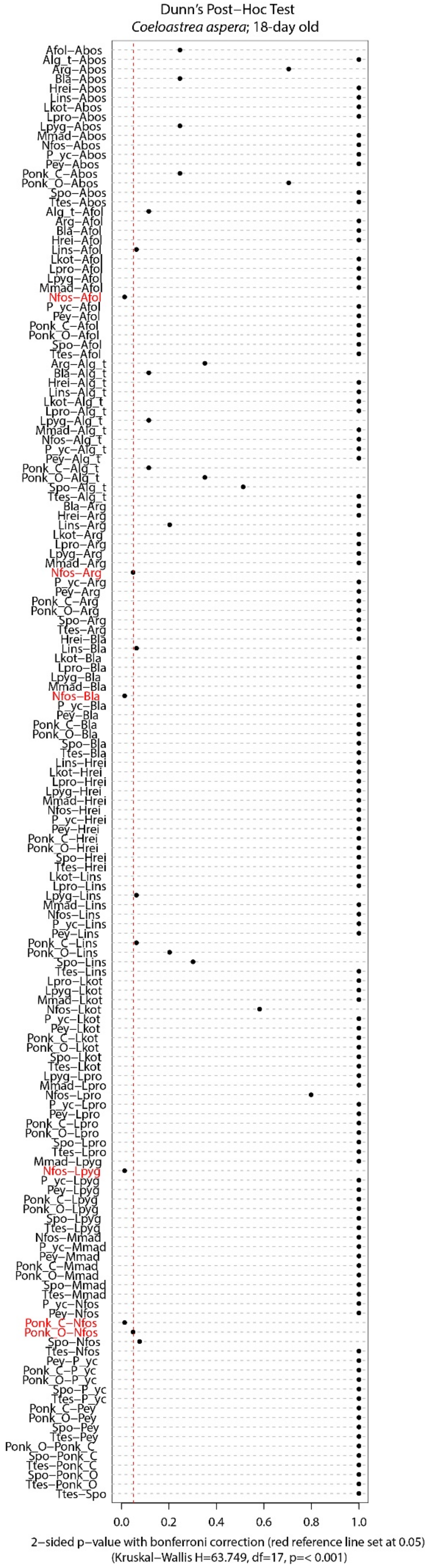

Figure S19: *Coeloastrea aspera.* 18-day old. Dunn's pairwise tests with Bonferroni correction. The red vertical dashed line indicates p-value of 0.05, with tests (dots) falling to the left the line showing significant tests. Alg_t refers to the coral rubble treatment and Pey refers to the *Ramicrusta* sp. treatment.

Caulastrea furcata

There was a significant effect of CCA treatments on *C. furcata* settlement (Kruskal-Wallis: H = 35.036, df = 17. p < 0.01), with 11 out of 15 CCA species inducing >75% mean settlement, and 100% settlement recorded for the *Ramicrusta* sp. and *N.* cf. *fosliei* treatments ($n_{treatment\ replicate}$ = 3, Table 3 of main article, Figure S20A and S21). There was no settlement in the blank treatment (0%) and low levels of settlement in the aragonite treatment (6.7 ± 6.7%) for *C. furcata* (Figure S20A). CCA species that induced moderate levels of mean settlement included *L.* cf. *proliferum* (63.3 ± 31.8%), *L.* cf. *insipidum* (66.7 ± 20.3%) and *P.* cf. *onkodes* "Orange" (59.0 ± 21.1%). The coral rubble treatment induced mean settlement of 93.6 ± 3.2%, with all CCA tested inducing statistically similar levels of settlement to the aragonite control (Dunn's test, p > 0.05, Figure S21), however it is notable that only 3 treatment replicates were used for this species compared to 12 used in other coral species assays. *Amphiroa* cf. *foliacea* induced settlement of 6.7 ± 6.7%, similar to the aragonite treatment. *Caulastrea furcata* is a generalist and would settle in the presence of most CCA species.

C. furcata settled in low numbers to the living surfaces of *P.* cf. *onkodes* "Yellow conceptacles" (3.0 ± 3.0%), *L.* cf. *insipidum* (3.3 ± 3.3%), *P.* cf. *onkodes* "Chalky" (3.3 ± 3.3%) and coral rubble chips (6.7 ± 6.7%).

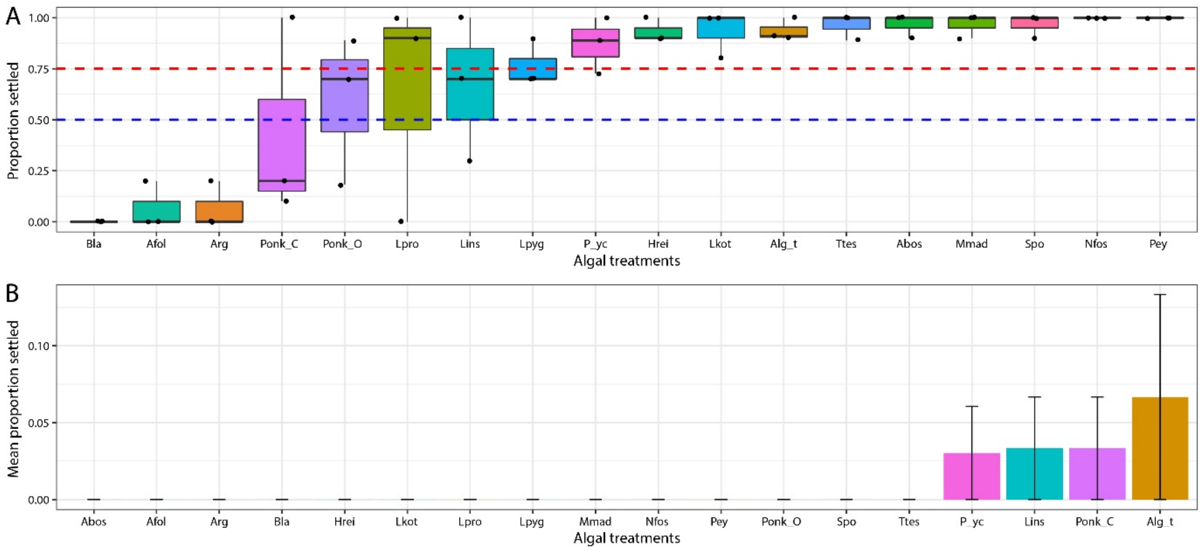

Figure S20: *Caulastrea furcata*. November. Larval settlement responses as proportion of total larvae counted at the end of the experiment that had successfully settled to the algal treatments, as A) box plots of total settlement and B) mean settlement (± SE) to the live surface of CCA chips. Responses to algal treatments are ordered in ascending order from left to right based on mean proportion settled. The horizontal blue dashed line on boxplots represents the 50% settlement threshold and horizontal red dashed line represents the 75% settlement threshold. Black dots represent data points for assay replicates within each algal treatment. Alg_t refers to the coral rubble treatment and Pey refers to the *Ramicrusta* sp. treatment.

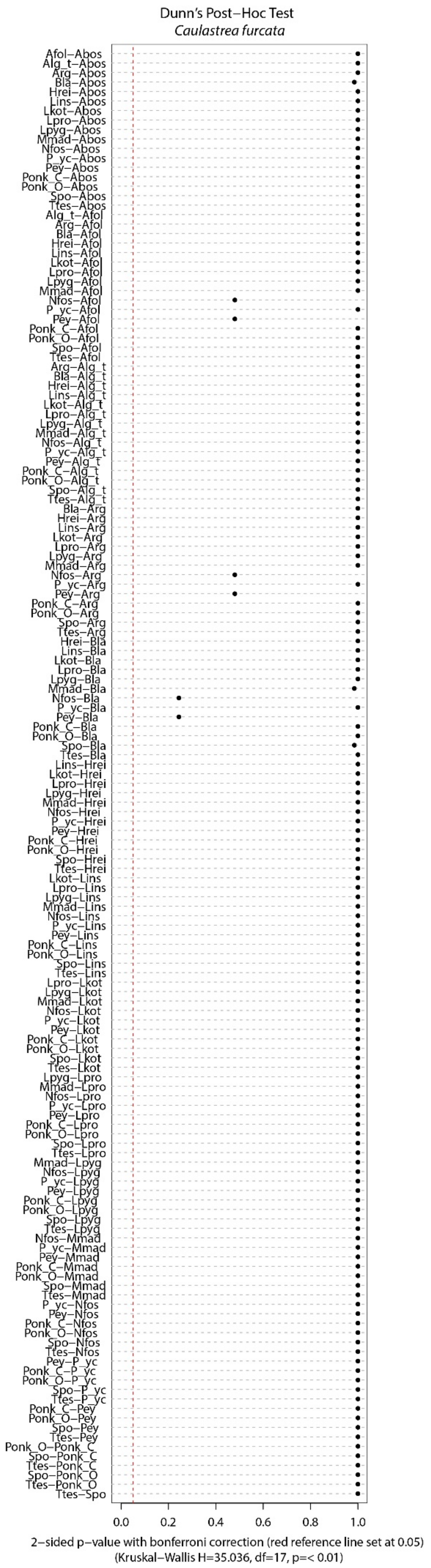

Figure S21: *Caulastrea furcata*. Dunn's pairwise tests with Bonferroni correction. The red vertical dashed line indicates p-value of 0.05, with tests (dots) falling to the left the line showing significant tests. Alg_t refers to the coral rubble treatment and Pey refers to the *Ramicrusta* sp. treatment.

Dipsastrea favus

There was a significant effect of CCA treatments on *D. favus* settlement (Kruskal-Wallis: H = 112.217, df = 17. p < 0.001), with 2 of the CCA tested inducing >75% mean settlement and that included *M.* cf. *madagascarense* (81.8 ± 3.7%) and *T.* cf. *tessellatum* (79.2 ± 6.7%; Table 3 of main article, Figure S22A). The blank treatment induced no settlement in *D. favus* (0%), with very low mean settlement recorded in the aragonite treatment (0.9 ± 0.9%, Figure S22A). *A.* cf. *bosencei*, *H.* cf. *reinboldii*, *L.* cf. *kotschyanum*, *M.* cf. *madagascariensis*, *P.* cf. *onkodes* "Yellow conceptacles", *Ramicrusta* sp. and *T.* cf. *tessellatum*, induced significantly higher settlement than the aragonite control (Dunn's test, p < 0.05; Figure S23). The coral rubble treatment induced moderate settlement (64.2 ± 6.1%) that is comparable to settlement found in all CCA treatments, except for *A.* cf. *foliacea* (2.7 ± 1.4%) that induced significantly lower settlement in *D. favus* (Dunn's test, p < 0.05; Figure S23), indicating this CCA species to be a poor inducer for *D. favus* settlement. CCA that induced moderate levels of settlement include *A.* cf. *boscensei* (71.5 ± 4.2%), *L.* cf. *kotschyanum* (70.2 ± 6.7%), *H.* cf. *reinboldii* (66.2 ± 8.8%), *P.* cf. *onkodes* "Yellow conceptacles" (65.8 ± 7.8%), *Ramicrusta* sp. (55.2 ± 8.3%), and *Sporolithon* sp. (51.1 ± 9.8%).

Settlement to the living surface of CCA treatment chips occurred at low levels <5% and were restricted to *A.* cf. *foliacea* (0.8 ± 0.8%), coral rubble (3.3 ± 1.9%) and *L.* cf. *pygmaeum* (4.2 ± 3.4%; Figure S22B)

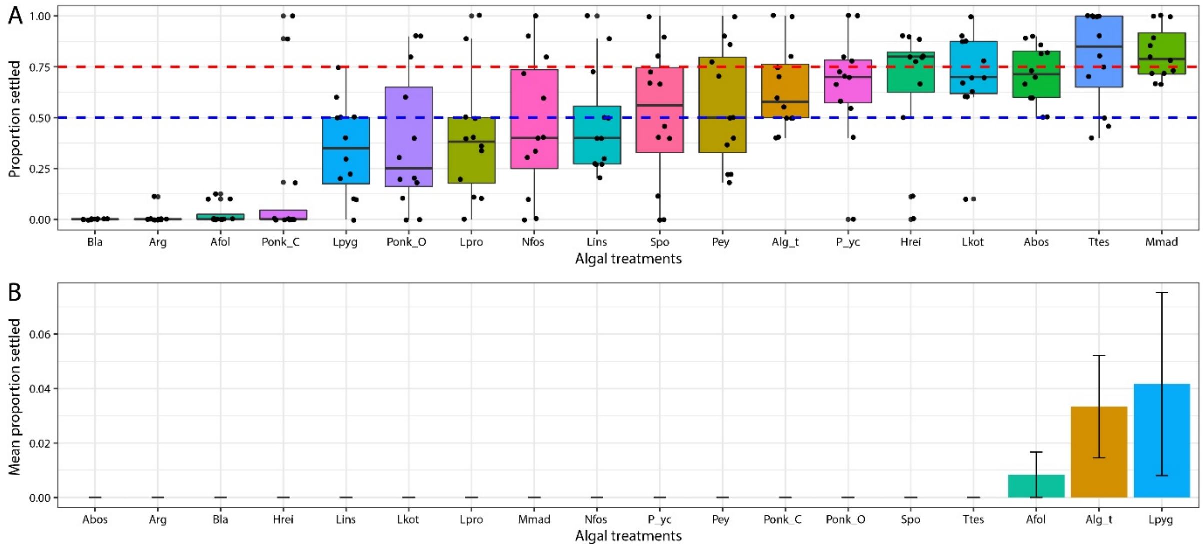

Figure S22: *Dipsastrea favus*. October. Larval settlement responses as proportion of total larvae counted at the end of the experiment that had successfully settled to the algal treatments, as A) box plots of total settlement and B) mean settlement (± SE) to the live surface of CCA chips. Responses to algal treatments are ordered in ascending order from left to right based on mean proportion settled. The horizontal blue dashed line on boxplots represents the 50% settlement threshold and horizontal red dashed line represents the 75% settlement threshold. Black dots represent data points for assay replicates within each algal treatment. Alg_t refers to the coral rubble treatment and Pey refers to the *Ramicrusta* sp. treatment.

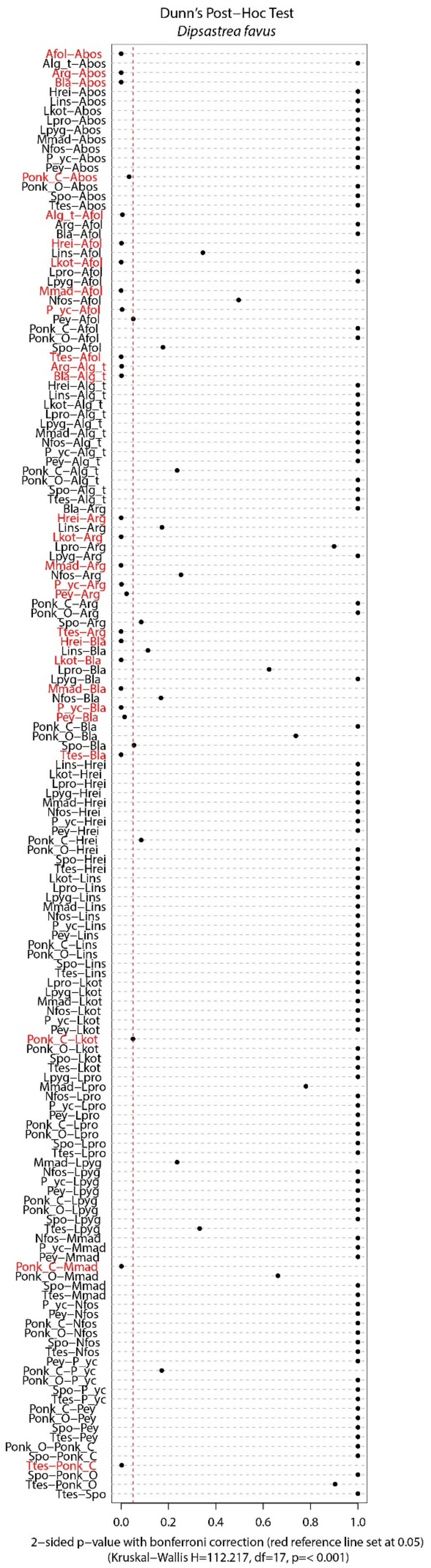

Figure S23: *Dipsatrea favus*. Dunn's pairwise tests with Bonferroni correction. The red vertical dashed line indicates p-value of 0.05, with tests (dots) falling to the left the line showing significant tests. Alg_t refers to the coral rubble treatment and Pey refers to the *Ramicrusta* sp. treatment.

Goniastrea favulus

There was a significant effect of CCA treatments on *G. favulus* settlement (Kruskal-Wallis: H = 129.211, df = 17. p < 0.001), with the blank, aragonite and *A.* cf. *foliacea* treatments not inducing any settlement (0%) and the coral rubble treatment inducing moderate settlement of 59.8 ± 8.7% (Table 3 of main article, Figure S24A). *A.* cf. *boscensei*, *L.* cf. *insipidum*, *L.* cf. *kotschyanum*, *L.* cf. *proliferum*, *M.* cf. *madagascariensis*, *N.* cf. *fosliei*, *Ramicrusta* sp., *Sporolithon* sp. and *T.* cf. *tessellatum* induced higher levels of settlement compared to the aragonite control (Dunn's test, p < 0.05; Figure S25). The highest mean settlement was seen in the *T.* cf. *tessellatum* treatment (76.8 ± 3.9%), with 5 CCA species (*Ramicrusta* sp., *A.* cf. *bosencei*, *L.* cf. *insipidum*, *L.* cf. *kotschyanum*, and *Sporolithon* sp.) inducing moderate settlement (50–70%) and the rest of CCA species inducing low levels of settlement (Figure S24A). Pairwise tests showed that the majority of CCA species induced similar levels of settlement to the coral rubble treatment, with *L.* cf. *pygmaeum* (3.3 ± 1.9%) and *P.* cf. *onkodes* "Chalky" (7.5 ± 4.5%) inducing significantly lower settlement indicating these species, in addition to *A.* cf. *foliacea*, to be poor inducers of settlement for *G. favulus* (Dunn's test, p < 0.05; Figure S25).

G. favulus larvae settled at low levels to the living surface of 5 of the CCA treatment chips, including coral rubble (0.8 ± 0.8%), *L.* cf. *kotschyanum* (0.8 ± 0.8%), *M.* cf. *madagascariensis* (0.9 ± 0.9%), *H.* cf. *reinboldii* (1.7 ± 1.1%) and *L.* cf. *pygmaeum* (1.7 ± 1.1%; Figure S24B).

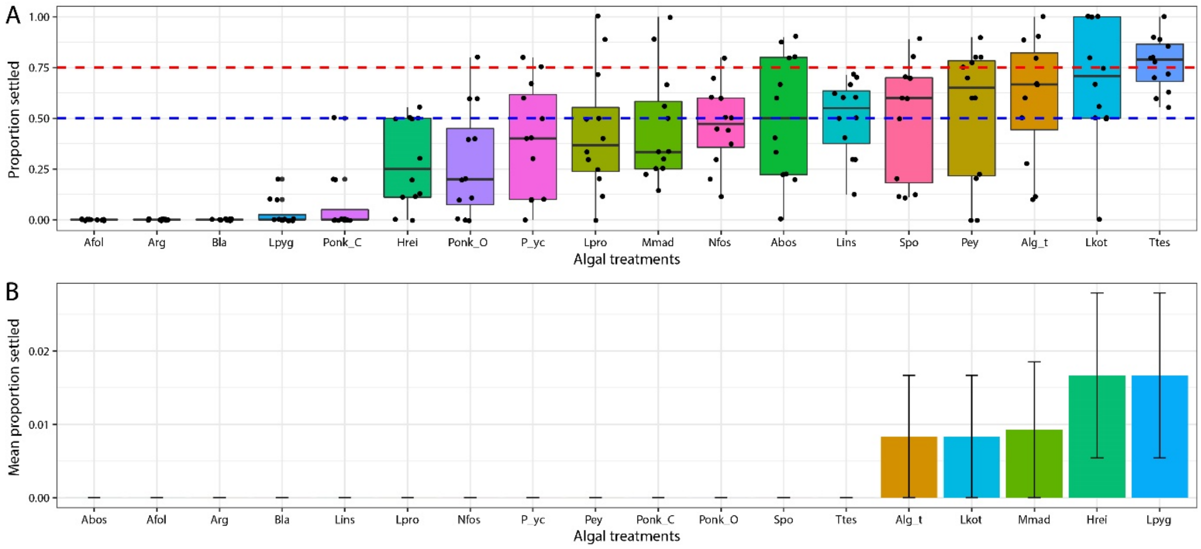

Figure S24: *Goniastrea favulus*. October. Larval settlement responses as proportion of total larvae counted at the end of the experiment that had successfully settled to the algal treatments, as A) box plots of total settlement and B) mean settlement (± SE) to the live surface of CCA chips. Responses to algal treatments are ordered in ascending order from left to right based on mean proportion settled. The horizontal blue dashed line on boxplots represents the 50% settlement threshold and horizontal red dashed line represents the 75% settlement threshold. Black dots represent data points for assay replicates within each algal treatment. Alg_t refers to the coral rubble treatment and Pey refers to the *Ramicrusta* sp. treatment.

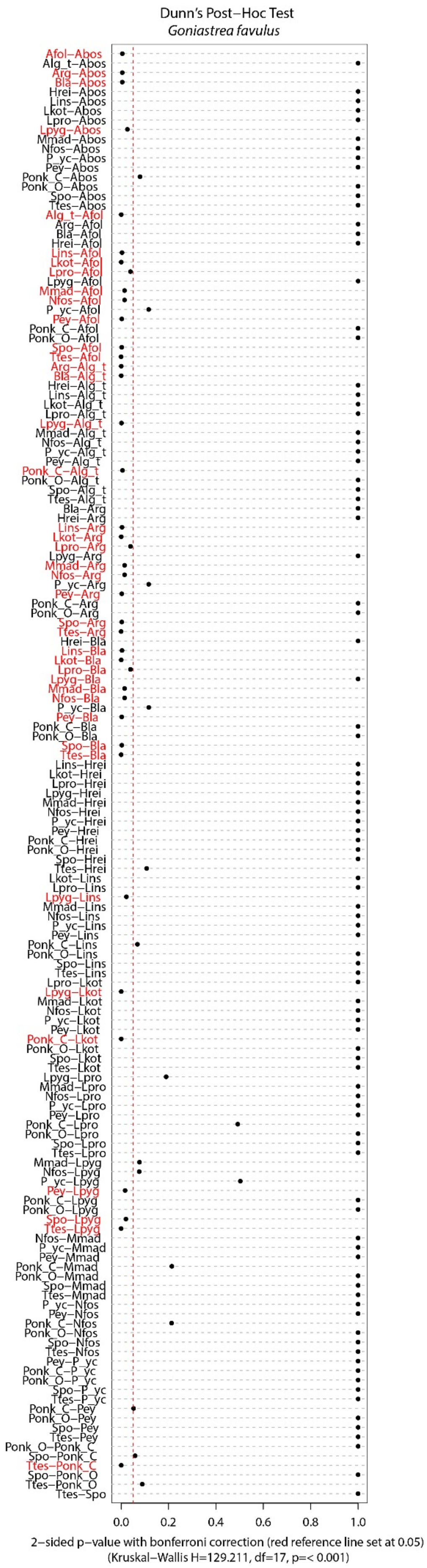

Figure S25: *Goniastrea favulus*. Dunn's pairwise tests with Bonferroni correction. The red vertical dashed line indicates p-value of 0.05, with tests (dots) falling to the left the line showing significant tests. Alg_t refers to the coral rubble treatment and Pey refers to the *Ramicrusta* sp. treatment.

Mycedium elephantotus

CCA treatments had a significant effect on larval settlement (Kruskal-Wallis: H = 123.999, df = 17. p < 0.001), with 5 out of the 15 CCA species inducing >75% settlement that include *T.* cf. *tessellatum* (93.3 ± 2.6%), *M.* cf. *madagascariensis* (85.4 ± 3.9%), *Sporolithon* sp. (82.6 ± 8.0%), *H.* cf. *reinboldii* (82.2 ± 5.9%) and *Ramicrusta* sp. (79.7 ± 4.3%; Table 3 of main article, Figure S26A). No settlement was found in the blank and aragonite treatments (0%) in *M. elephantotus*, with mean settlement in the coral rubble treatment being 76.8 ± 6.2% (Figure S26A). *H.* cf. *reinboldii*, *L.* cf. *insipidum*, *L.* cf. *kotschyanum*, *L.* cf. *proliferum*, *M. cf. madagascariensis*, *P.* cf. onkodes "Yellow conceptacles", *Ramicrusta* sp., *Sporolithon* sp. and *T.* cf. *tessellatum* induced significantly higher settlement compared to the aragonite control (Dunn's test, p < 0.05; Figure S27). All CCA species induced a similar settlement response compared to the coral rubble treatment except for *P.* cf. *onkodes* "Orange" and *A.* cf. *foliacea* which recorded significantly lower settlement of 16.9 ± 7.7% and 2.5 ± 2.5% respectively (Dunn's test, p < 0.05; Figure S27). *M. elephantotus* did not settle to the living surface of any of the CCA treatment chips (Figure S26B).

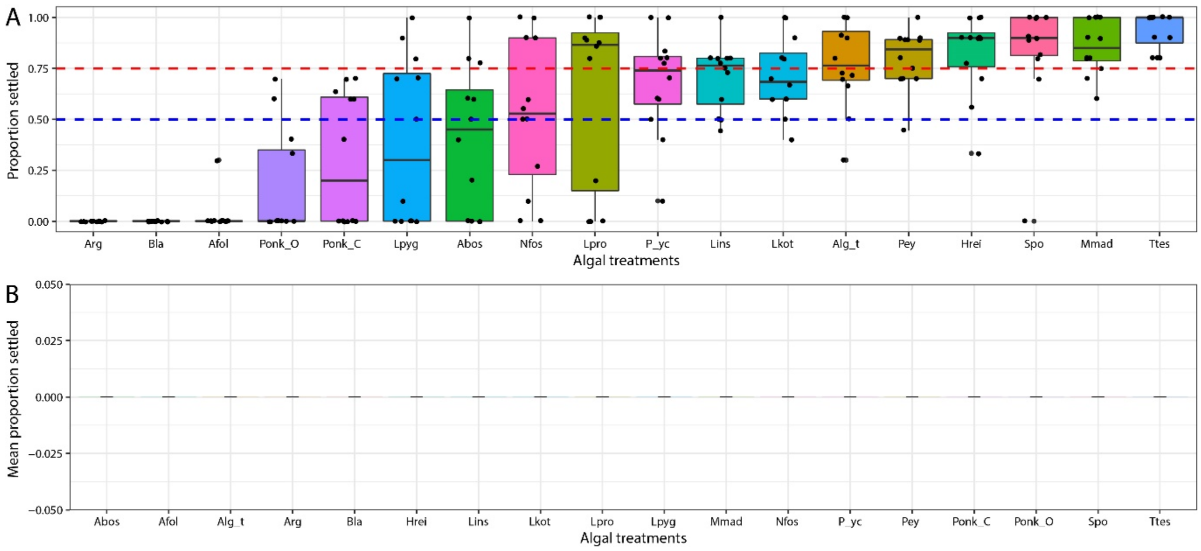

Figure S26: *Mycedium elephantotus*. November. Larval settlement responses as proportion of total larvae counted at the end of the experiment that had successfully settled to the algal treatments, as A) box plots of total settlement and B) mean settlement (± SE) to the live surface of CCA chips. Responses to algal treatments are ordered in ascending order from left to right based on mean proportion settled. The horizontal blue dashed line on boxplots represents the 50% settlement threshold and horizontal red dashed line represents the 75% settlement threshold. Black dots represent data points for assay replicates within each algal treatment. Alg_t refers to the coral rubble treatment and Pey refers to the *Ramicrusta* sp. treatment.

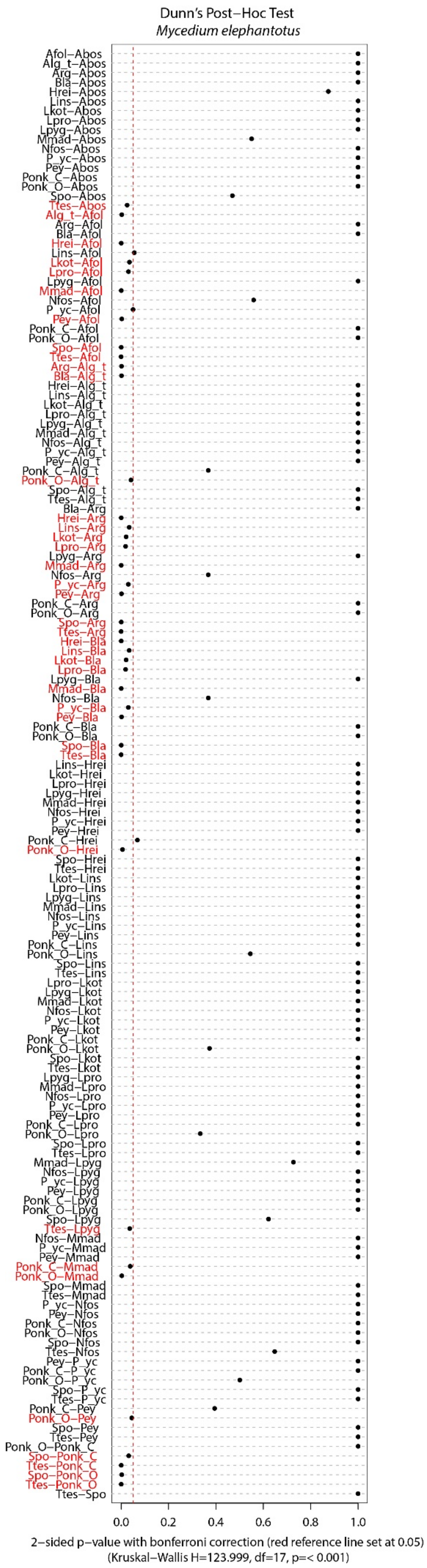

Figure S27: *Mycedium elephantotus*. Dunn's pairwise tests with Bonferroni correction. The red vertical dashed line indicates p-value of 0.05, with tests (dots) falling to the left the line showing significant tests. Alg_t refers to the coral rubble treatment and Pey refers to the *Ramicrusta* sp. treatment.

Platygyra sinensis

CCA treatments had a significant effect on the settlement of *P. sinensis*, with the coral rubble treatment inducing 80.3 ± 5.6% settlement and 6 out of 15 CCA species inducing >75% mean settlement in *P. sinensis* (Kruskal-Wallis: H = 117.784, df = 17. p < 0.001). There was no settlement in the blank treatment and very low settlement (0.8 ± 0.8%) in the aragonite treatment for *P. sinensis* (Table 3 of main article, Figure S28A). *A.* cf. *bosencei*, *L.* cf. *kotschyanum*, *L.* cf. *proliferum*, *M.* cf. *madagascariensis*, *Ramicrusta* sp., *P.* cf. *onkodes* "Orange" and *T.* cf. *tessellatum* induced significantly higher settlement compared to the aragonite control (Dunn's test, p <0.05, Figure S29). Highly inducive CCAs include *A.* cf. *bosencei* (92.5 ± 2.6%), *L.* cf. *kotschyanum* (89.2 ± 3.8%), *T.* cf. *tessellatum* (81.4 ± 6.4%), *L.* cf. *proliferum* (78.5 ± 2.6%), *M.* cf. *madagascariensis* (76.2 ± 5.9%) and *Ramicrusta* sp. (75.8 ± 4.3%; Figure S28A). Pairwise tests showed that most CCA species induced similar settlement compared to the coral rubble treatment, except for *L.* cf. *pygmaeum* and *A.* cf. *foliacea* that recorded significantly lower settlement of 10.8 ± 7.4% and 0.8 ± 0.8% respectively (Dunn's test, p <0.05, Figure S29).

P. sinensis larvae settled in low numbers to the living surface of *A.* cf. *foliacea* (0.8 ± 0.8%), *L.* cf. *pygmaeum* (0.8 ± 0.8%) and coral rubble treatment chips (1.7 ± 1.7%; Figure S28B).

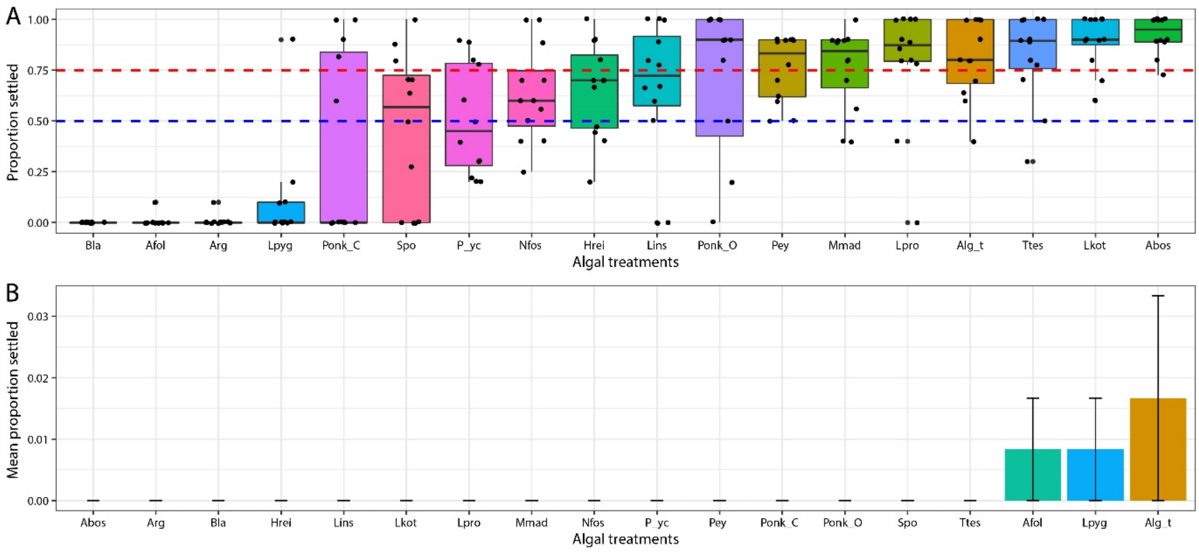

Figure S28: *Platygyra sinensis*. October. Larval settlement responses as proportion of total larvae counted at the end of the experiment that had successfully settled to the algal treatments, as A) box plots of total settlement and B) mean settlement (± SE) to the live surface of CCA chips. Responses to algal treatments are ordered in ascending order from left to right based on mean proportion settled. The horizontal blue dashed line on boxplots represents the 50% settlement threshold and horizontal red dashed line represents the 75% settlement threshold. Black dots represent data points for assay replicates within each algal treatment. Alg_t refers to the coral rubble treatment and Pey refers to the *Ramicrusta* sp. treatment.

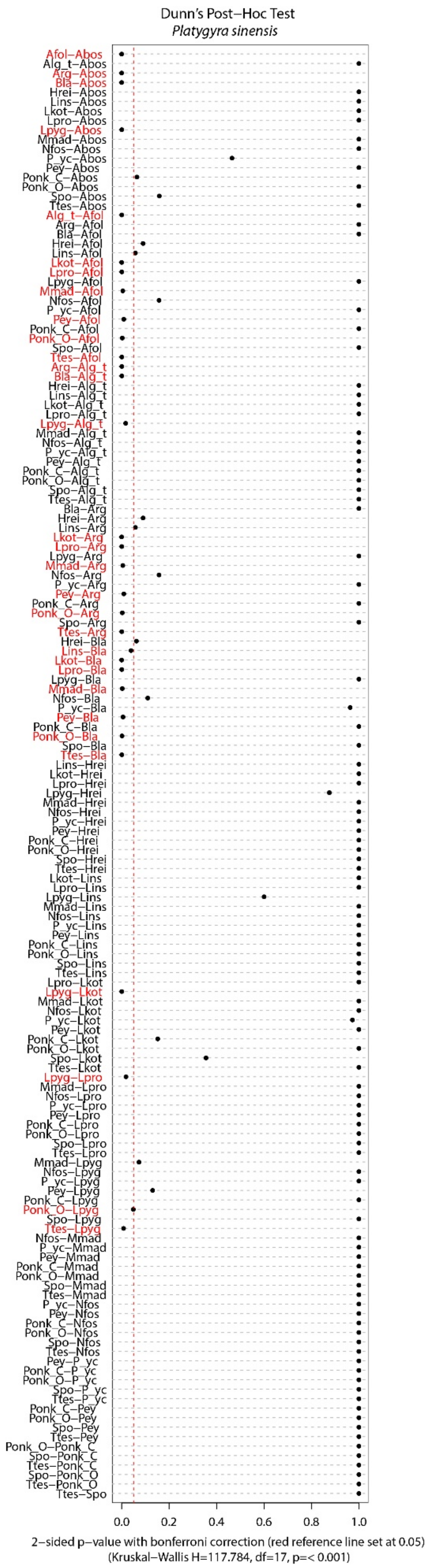

Figure S29: *Platygyra sinensis*. Dunn's pairwise tests with Bonferroni correction. The red vertical dashed line indicates p-value of 0.05, with tests (dots) falling to the left the line showing significant tests. Alg_t refers to the coral rubble treatment and Pey refers to the *Ramicrusta* sp. treatment.

Platygyra daedalea

CCA treatments had a significant effect on settlement with 14 out 15 CCA species inducing >75% mean settlement (Kruskal-Wallis: H = 129.551, df = 17. p < 0.001, Table 3 of main article, Figure S30A). There were no settlement of *P. daedalea* larvae in the blank and aragonite treatments. *Platygyra daedalea* larvae are generalist settlers with the coral rubble and *M.* cf. *madagascariensis* treatments inducing 100% settlement, and 10 other CCA species inducing mean settlement of >90% (Figure S30A). All CCA treatment chips induced significantly higher settlement compared to the aragonite control except for *A.* cf. *foliacea*, *L.* cf. *proliferum* and *L.* cf. *pygmaeum* (Dunn's test, p > 0.05, Figure S31). *Porolithon* cf. *onkodes* "Chalky", *L.* cf. *pygmaeum* and *L.* cf. *proliferum* induced high settlement at 89.2 ± 6.1%, 79.2 ± 7.6% and 77.1 ± 8.8% respectively. *Amphiroa* cf. *foliacea*, which induced low settlement (<20%) in most other coral species tested, induced a mean settlement of 56.3 ± 8.0% in *P. daedalea*. Pairwise tests showed that all CCA species induced similar levels of settlement to the coral rubble treatment except for *A.* cf. *foliacea*, which induced significantly lower settlement (Dunn's test, p < 0.05, Figure S31). *P. daedalea* larvae did not settle to the living surface of any of the CCA treatment chips (Figure S30B).

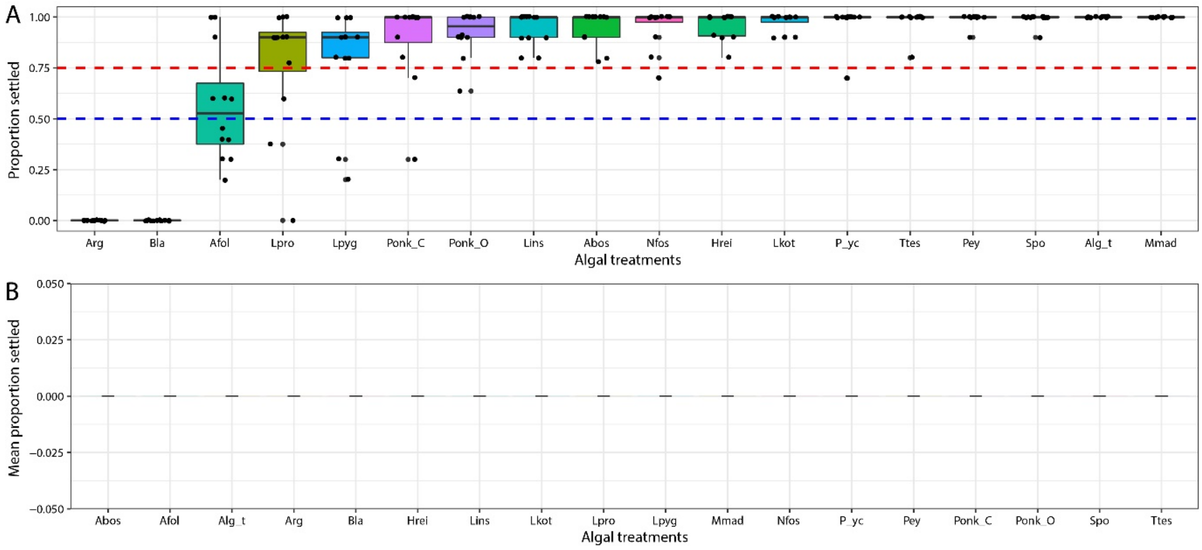

Figure S30: *Platygyra daedalea*. November. Larval settlement responses as proportion of total larvae counted at the end of the experiment that had successfully settled to the algal treatments, as A) box plots of total settlement and B) mean settlement (± SE) to the live surface of CCA chips. Responses to algal treatments are ordered in ascending order from left to right based on mean proportion settled. The horizontal blue dashed line on boxplots represents the 50% settlement threshold and horizontal red dashed line represents the 75% settlement threshold. Black dots represent data points for assay replicates within each algal treatment. Alg_t refers to the coral rubble treatment and Pey refers to the *Ramicrusta* sp. treatment.

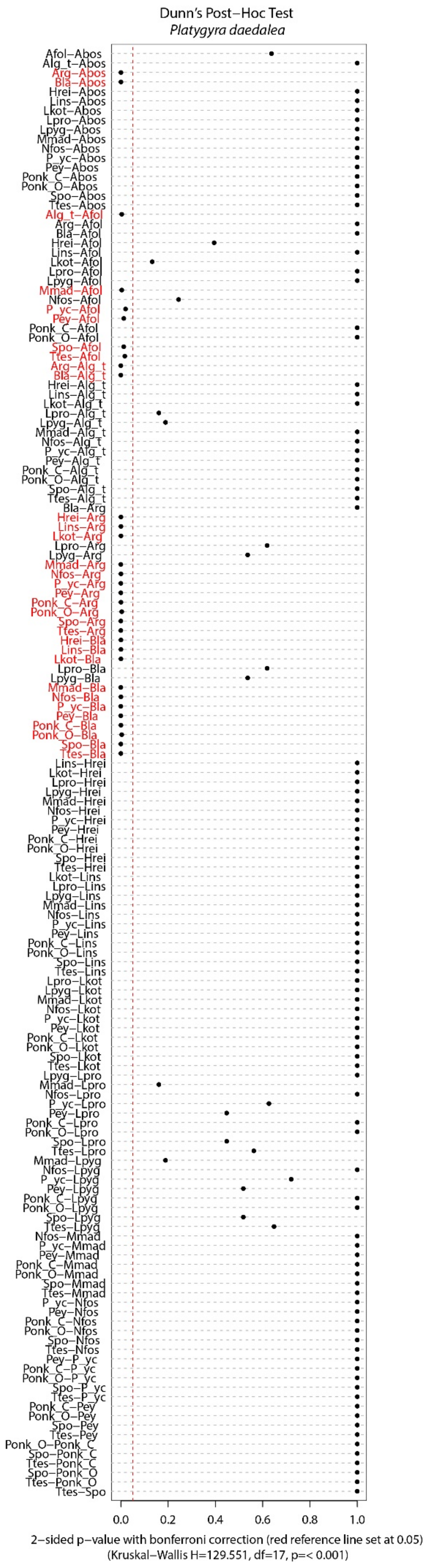

Figure S31: *Platygyra daedalea*. Dunn's pairwise tests with Bonferroni correction. The red vertical dashed line indicates p-value of 0.05, with tests (dots) falling to the left the line showing significant tests. Alg_t refers to the coral rubble treatment and Pey refers to the *Ramicrusta* sp. treatment.

LOBOPHYLLIIDAE

Echinophyllia aspera

CCA treatments significantly influenced larval settlement, with the highest mean settlement found in the *Sporolithon* sp. treatment (86.4 ± 5.5%), followed by *Ramicrusta* sp. (85.2 ± 2.5%) and *T.* cf. *tessellatum* (80.6 ± 7.2%; Kruskal-Wallis: H = 111.872, df = 17. $p < 0.001$; Table 3 of main article, Figure S32A). The blank treatment did not induce any settlement (0%) and aragonite treatment induced very low settlement (0.8 ± 0.8%) in *E. aspera* larvae. *A.* cf. *boscensei*, *L.* cf. *insipidum*, *L.* cf. *kotschyanum*, *M. cf. madagascariensis*, *Ramicrusta* sp., *P.* cf. *onkodes* "Orange", *Sporolithon* sp. and *T.* cf. *tessellatum* induced significantly higher settlement compared to the aragonite control (Dunn's test, $p < 0.05$; Figure S33). Settlement in the coral rubble treatment was 70.8 ± 7.9%. Other CCA that induced high settlement in *E. aspera* larvae (>75%) included *P.* cf. *onkodes* "Orange" (80.0 ± 6.9%), *A.* cf. *boscensei* (79.1 ± 5.6%), *L.* cf. *insipidum* (78.6 ± 5.1%) and *L.* cf. *kotschyanum* (77.9 ± 6.6%; Table S3). Pairwise comparisons showed no difference in settlement between the coral rubble treatment to any of the CCA species, except for *A.* cf. *foliacea* which induced significantly lower settlement (3.3 ± 3.3%; Dunn's test, $p < 0.05$; Figure S33).

E. aspera larvae settled to the living surface of 4 CCA treatment chips including *L.* cf. *kotschyanum* (0.8 ± 0.8%), coral rubble (0.9 ± 0.9%), *P.* cf. *onkodes* "Yellow conceptacle" and *L.* cf. *pygmaeum* (15.8 ± 5.1%; Figure S32B).

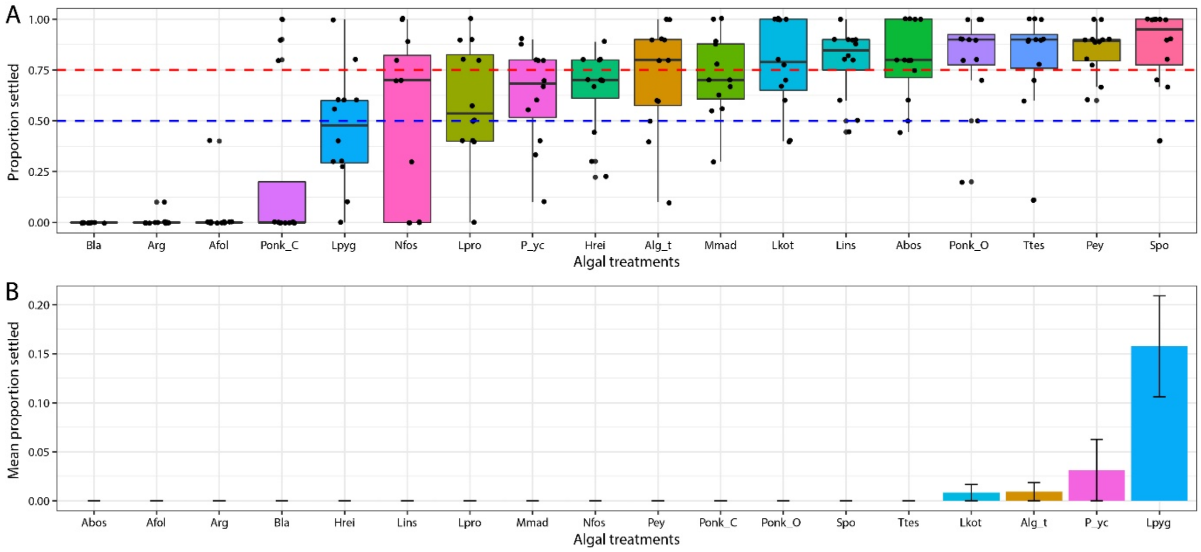

Figure S32: *Echinophyllia aspera*. October. Larval settlement responses as proportion of total larvae counted at the end of the experiment that had successfully settled to the algal treatments, as A) box plots of total settlement and B) mean settlement (± SE) to the live surface of CCA chips. Responses to algal treatments are ordered in ascending order from left to right based on mean proportion settled. The horizontal blue dashed line on boxplots represents the 50% settlement threshold and horizontal red dashed line represents the 75% settlement threshold. Black dots represent data points for assay replicates within each algal treatment. Alg_t refers to the coral rubble treatment and Pey refers to the *Ramicrusta* sp. treatment.

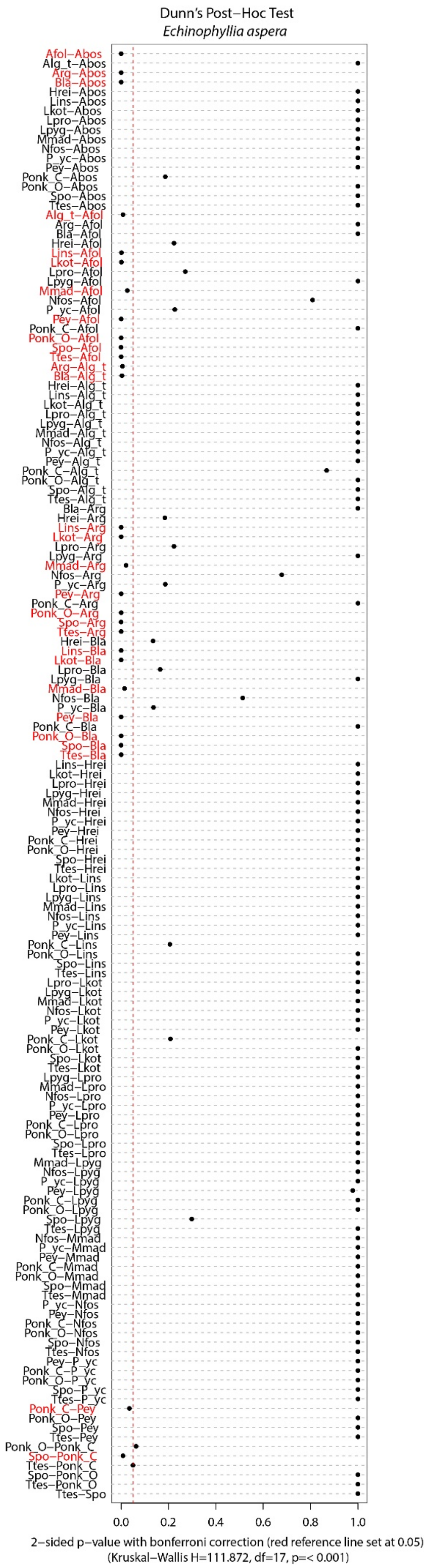

Figure S33: *Echinophyllia aspera*. Dunn's pairwise tests with Bonferroni correction. The red vertical dashed line indicates p-value of 0.05, with tests (dots) falling to the left the line showing significant tests. Alg_t refers to the coral rubble treatment and Pey refers to the *Ramicrusta* sp. treatment.

Lobophyllia corymbosa

CCA treatments had a significant effect on settlement, with the highest settlement found in the *Sporolithon* sp. treatment (97.4 ± 5.5%; Kruskal-Wallis: H = 118.491, df = 17. p < 0.001; Table 3 of main article, Figure S34A). There was no settlement (0%) of *L. corymbosa* larvae in the blank and aragonite treatments. *H.* cf. *reinboldii*, *L.* cf. *insipidum*, *L.* cf. *kotschyanum*, *L.* cf. *proliferum*, *M.* cf. *madagascariensis*, *Ramicrusta* sp., *Sporolithon* sp. and *T.* cf. *tessellatum*, induced significantly higher settlement compared to the aragonite control (Dunn's test; p < 0.05, Figure S35). High settlement (>75%) was found in one other CCA, *M.* cf. *madagascariensis* (75.0 ± 6.7%), while 4 out 15 CCA tested induced moderate levels of settlement that included *Ramicrusta* sp. (66.5 ± 6.9%), *T.* cf. *tessellatum* (62.5 ± 9.9%), *L.* cf. *proliferum* (52.1 ± 9.9%) and *H.* cf. *reinboldii* (51.4 ± 7.4%; Figure S34A). The coral rubble treatment induced 51.8 ± 6.1% settlement. Pairwise tests showed that all CCA species induced similar levels of settlement to the coral rubble treatment (Dunn's test; p > 0.05, Figure S35).

Settlement of *L. corymbosa* larvae to living surface of CCA treatment chips occurred in *L.* cf. *kotschyanum* and *M.* cf. *madagascariensis*, however in very low numbers (both 0.8 ± 0.8%; Figure S34B)

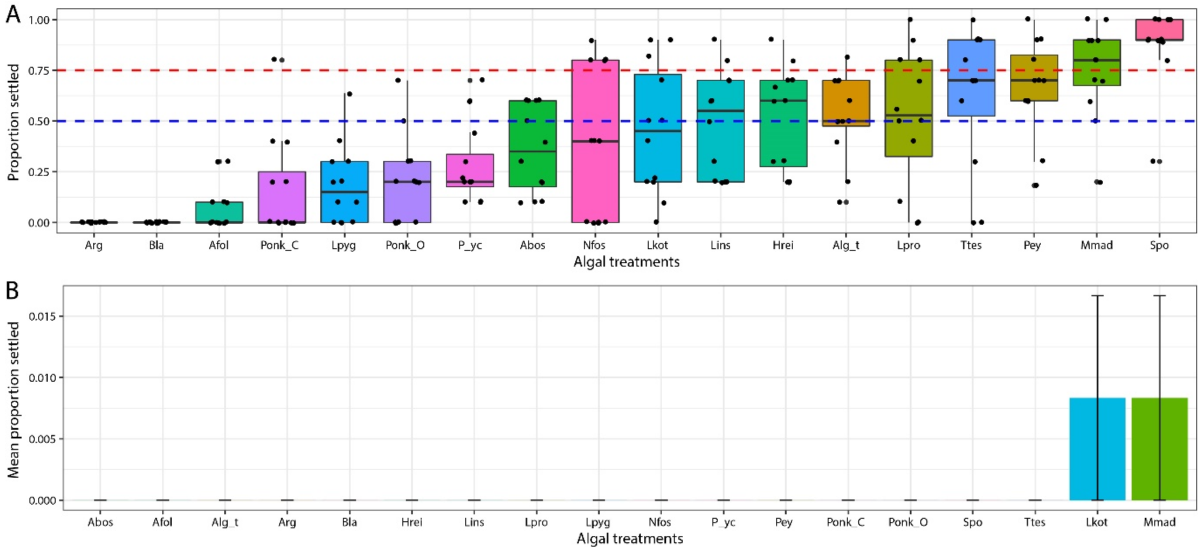

Figure S34: *Lobophyllia corymbosa*. November. Larval settlement responses as proportion of total larvae counted at the end of the experiment that had successfully settled to the algal treatments, as A) box plots of total settlement and B) mean settlement (± SE) to the live surface of CCA chips. Responses to algal treatments are ordered in ascending order from left to right based on mean proportion settled. The horizontal blue dashed line on boxplots represents the 50% settlement threshold and horizontal red dashed line represents the 75% settlement threshold. Black dots represent data points for assay replicates within each algal treatment. Alg_t refers to the coral rubble treatment and Pey refers to the *Ramicrusta* sp. treatment.

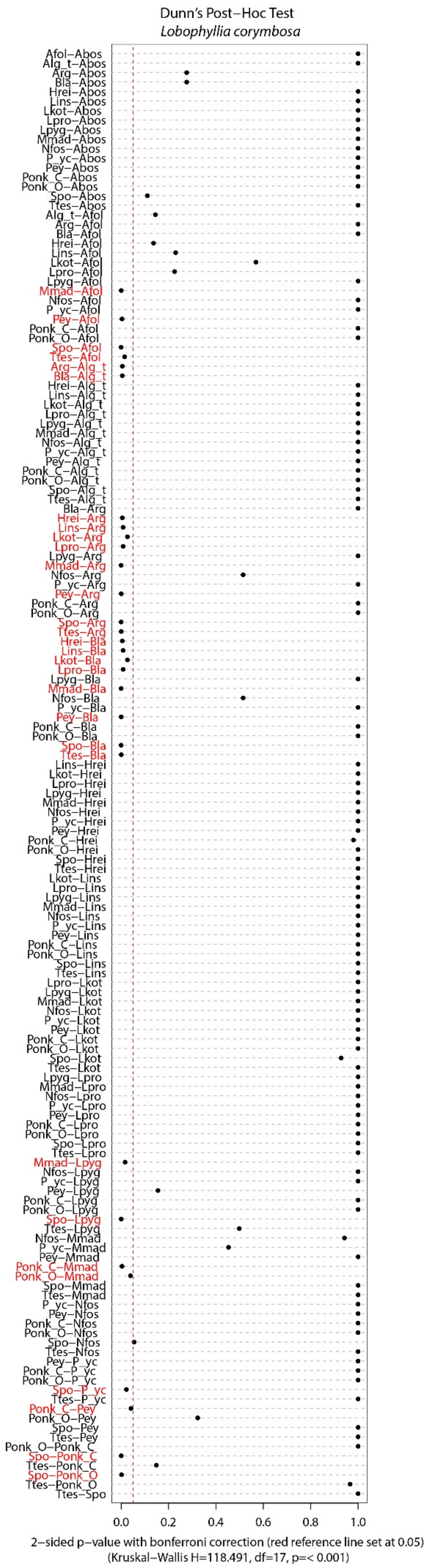

Figure S35: *Lobophyllia corymbosa*. Dunn's pairwise tests with Bonferroni correction. The red vertical dashed line indicates p-value of 0.05, with tests (dots) falling to the left the line showing significant tests. Alg_t refers to the coral rubble treatment and Pey refers to the *Ramicrusta* sp. treatment.

PORITIDAE

Porites lobata

CCA treatments had a significant effect on larval settlement, with the coral rubble treatment inducing 86.6 ± 6.3% settlement and highest settlement detected in the *Ramicrusta* sp. treatment (92.5 ± 4.5%), followed by *L.* cf. *insipidum* (91.9 ± 3.3%) and *T.* cf. *tessellatum* (90.4 ± 2.4%; Kruskal-Wallis: H = 101.751, df = 17. p < 0.001; Table 3 of main article, Figure S36A). There was no settlement (0%) of *P. lobata* larvae in the blank treatment and very low settlement (0.9 ± 5.5%) found in the aragonite treatment (Figure S36A). *A.* cf. *boscensei*, *H.* cf. *reinboldii*, *L.* cf. *insipidum*, *L.* cf. *kotschyanum*, *N.* cf. *fosliei*, *Ramicrusta* sp., *P.* cf. *onkodes* "Chalky", *P.* cf. *onkodes* "Orange" and *T.* cf. *tessellatum* induced significantly higher settlement compared to the aragonite control (Dunn's test; p < 0.05, Figure S37). Four other CCAs induced high larval settlement (>75%) in *P. lobata* that included *N.* cf. *fosliei* (85.5 ± 3.8%), *L.* cf. *kotschyanum* (85.4 ± 5.9%), *A.* cf. *boscensei* (78.8 ± 5.1%) and *P.* cf. *onkodes* "Orange" (77.6 ± 6.5%; Figure S36A). Lowest settlement was found in the *L.* cf. *proliferum* treatment (40.3 ± 11.8%). Pairwise test showed no difference in settlement to any of the CCA when compared to the coral rubble treatment (Dunn's test; p > 0.05, Figure S37).

P. lobata settled onto the living surfaces of 12 of the CCA treatment chips, with highest settlement found in *T.* cf. *tessellatum* (46.2 ± 8.4%), followed by coral rubble (19.4 ± 6.2%) and *P.* cf. *onkodes* "Yellow conceptacle" (14.6 ± 4.8%; Figure S36B).

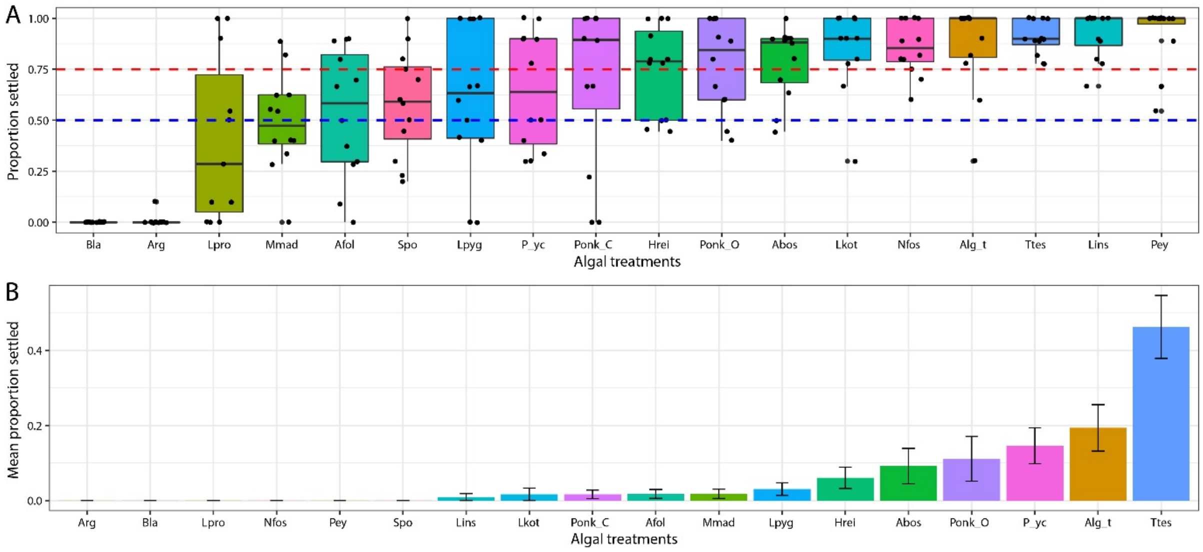

Figure S36: *Porites lobata*. November. Larval settlement responses as proportion of total larvae counted at the end of the experiment that had successfully settled to the algal treatments, as A) box plots of total settlement and B) mean settlement (± SE) to the live surface of CCA chips. Responses to algal treatments are ordered in ascending order from left to right based on mean proportion settled. The horizontal blue dashed line on boxplots represents the 50% settlement threshold and horizontal red dashed line represents the 75% settlement threshold. Black dots represent data points for assay replicates within each algal treatment. Alg_t refers to the coral rubble treatment and Pey refers to the *Ramicrusta* sp. treatment.

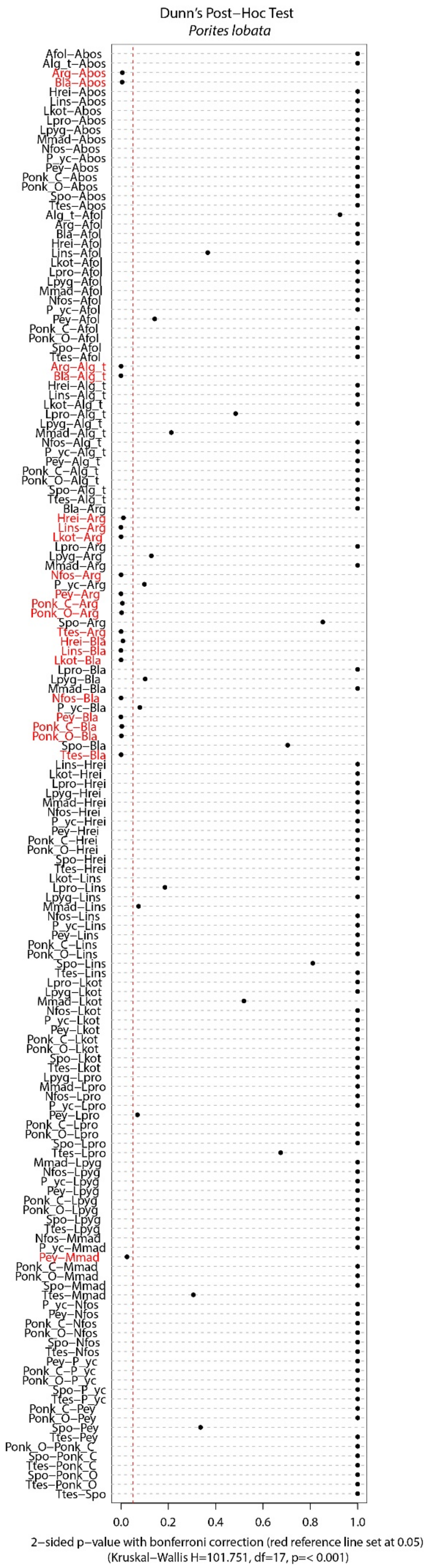

Figure S37: *Porites lobata*. Dunn's pairwise tests with Bonferroni correction. The red vertical dashed line indicates p-value of 0.05, with tests (dots) falling to the left the line showing significant tests. Alg_t refers to the coral rubble treatment and Pey refers to the *Ramicrusta* sp. treatment.

FUNGIIDAE

Fungia fungites

There was a significant effect of CCA treatments *F. fungites* larval settlement (Kruskal-Wallis: H = 58.958, df = 17. $p < 0.001$). Larval settlement was in general low for *F. fungites* across all CCA treatments tested and did not exceed 32% (Table 3 in main article, Figure S38A). The coral rubble treatment induced 13.7 % ± 5.2% settlement, with the highest settlement found in the *Sporolithon* sp. treatment (31.6 ± 9.1%) followed by *M.* cf. *madagascariensis* (24.4 ± 6.0%) and *Ramicrusta* sp. (23.7 ± 9.2%). Pairwise tests showed similar levels of settlement across all CCA treatments when compared to the coral rubble treatment, with only *M.* cf. *madagascariensis* and *Sporolithon* sp. inducing significantly higher settlement than the aragonite control (Dunn's test; $p < 0.05$, Figure S39). *F. fungites* larvae did not settle to the living surface of any of the CCA treatment chips (Figure S38B).

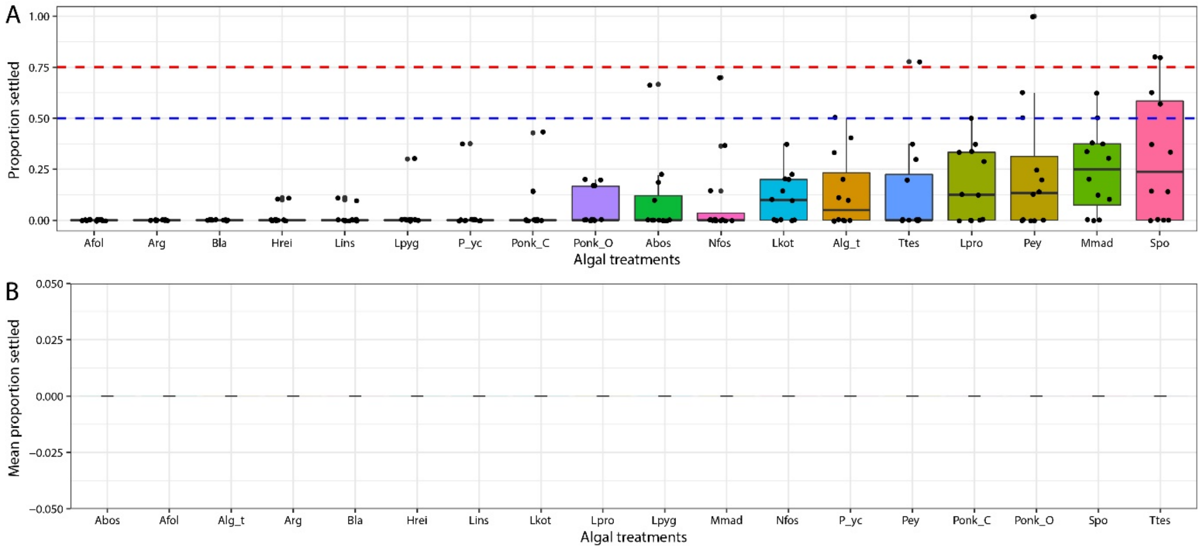

Figure S38: *Fungia fungites*. November. Larval settlement responses as proportion of total larvae counted at the end of the experiment that had successfully settled to the algal treatments, as A) box plots of total settlement and B) mean settlement (± SE) to the live surface of CCA chips. Responses to algal treatments are ordered in ascending order from left to right based on mean proportion settled. The horizontal blue dashed line on boxplots represents the 50% settlement threshold and horizontal red dashed line represents the 75% settlement threshold. Black dots represent data points for assay replicates within each algal treatment. Alg_t refers to the coral rubble treatment and Pey refers to the *Ramicrusta* sp. treatment.

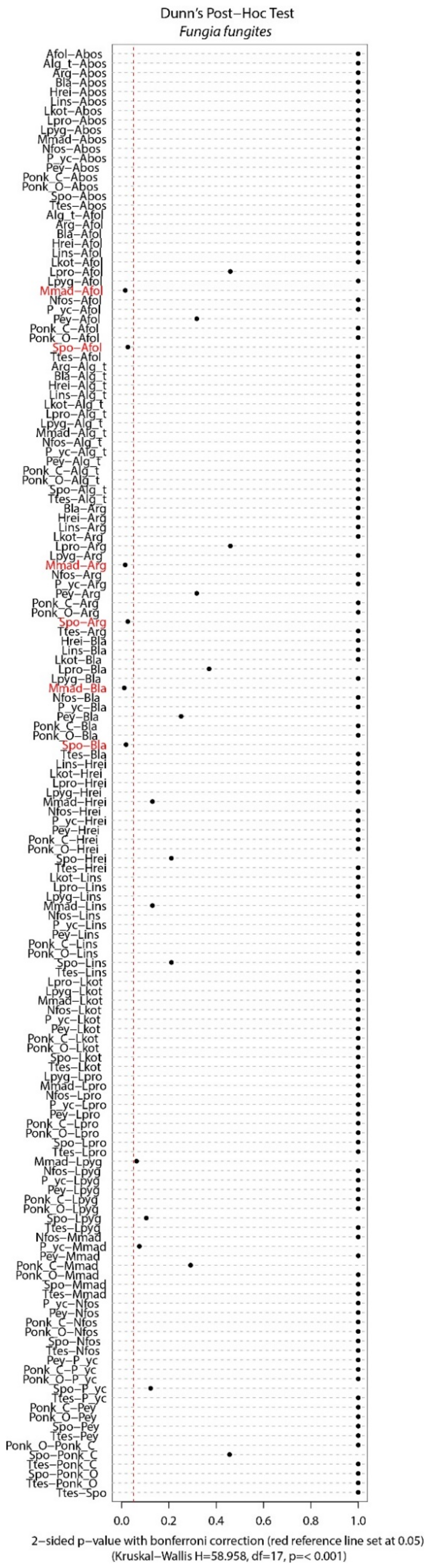

Figure S39: *Fungia fungites*. Dunn's pairwise tests with Bonferroni correction. The red vertical dashed line indicates p-value of 0.05, with tests (dots) falling to the left the line showing significant tests. Alg_t refers to the coral rubble treatment and Pey refers to the *Ramicrusta* sp. treatment.

Coral family-specific settlement behaviours to CCAs

CCA inductive properties within Acroporidae

Three *Acropora* spp. and one *Montipora* sp. were tested from the family Acroporidae (n_{assay} = 48 per CCA species Table 3 of main article). There was a significant effect of CCA treatment on larval settlement when all Acroporidae species were considered together (Kruskal-Wallis: H = 317.669, df = 17, p<0.001). The best performing CCA were *L.* cf. *insipidum* (85.7%), *T.* cf. *tessellatum* (81.3%), *L.* cf. *kotschyanum* (80.2%) and *P.* cf. *onkodes* "Yellow conceptacles" (72.4%; Table S3, Figure S40B). Dunn's pairwise tests showed that most CCA species induced settlement comparable to the coral rubble treatment except *A.* cf. *foliacea* and *L.* cf. *proliferum*, which induced lower settlement (p<0.05). The other *Porolithon* spp., including *P.* cf. *onkodes* "Chalky" and *P.* cf. *onkodes* "Orange" were moderately inductive for acroporid larvae with mean settlement of ~63%.

Inter-generic differences in settlement were apparent within the Acroporidae, with high settlement in response to the *L.*cf. *pygmaeum* treatment for all *Acropora* spp. (mean settlement range: 76.9–88.2%) but only 3.3% in *M. aequituberculata* (Table 3 of main article). Similarly, *Acropora* spp. also settled well in response to *P.* cf. *onkodes* "Chalky" (62.4–90.5%) and *T.* cf. *tessellatum* (80.6–96.0%), whereas *M. aequituberculata* settled less well in those treatments (22.7% and 59.7%, respectively; Table 3 of main article). By contrast, *L.* cf. *insipidum* was inductive across all species tested in the Acroporidae.

Some inter-specific settlement patterns were evident within the genus *Acropora*, whereby *A. hyacinthus* larvae were more selective than *A. tenuis* larvae (Table 3 of main article). While >75% settlement by both species occurred in the *L.* cf. *insipidum*, *L.* cf. *pygmaeum* and *T.* cf. *tessellatum* treatments, settlement was ~50% lower in *A. hyacinthus* in the presence of moderate–low light adapted CCA species *(Ramicrusta* sp., *L.* cf. *proliferum*, *M*. cf. *madagascariensis*, *A.* cf. *boscensei*, *H.* cf. *reinboldii* and *Sporolithon* sp.; Table 3 of main article). *Amphiroa* cf. *foliacea* did not perform well (<50% settlement) in any of the acroporid species tested.

All species of Acroporidae settled onto the living surface of at least some CCA species, with *A. tenuis* exhibiting live-surface settlement on most of CCA (13), followed by *A. anthocercis* (12), *A. hyacinthus* (10) and *M. aequituberculata* (5; Table S2). The CCA species that induced live surface settlement across all Acroporid species, and in descending level of success, were *T.* cf. *tessellatum*, *L.* cf. *kotschyanum*, *L.* cf. *insipidum* and *H.* cf. *reinboldii*, with the former 3 CCA belonging to the family Lithophyllaceae (Table S2).

CCA inductive properties within Merulinidae

Seven species from the family Merulinidae were tested (n_{assay} = 69 per CCA species), and CCA treatment had a significant effect on larval settlement at the family level (Kruskal-Wallis: H = 472.47, df = 17, p<0.001; Table S3, Figure S40C). Most CCAs induced similar levels of settlement when compared to the coral rubble treatment except *P.* cf. *onkodes* "Orange", *L.* cf. *pygmaeum*, *P.* cf. *onkodes* "Chalky" and *A.* cf. *foliacea,* which induced significantly lower settlement than coral rubble (Table S3, Figure S40C). Settlement to *A. cf. foliacea* was similar to that found in the sterile aragonite treatment (p>0.05).

Within the Merulinidae, a broad range of responses were found, with some species considered to be generalist settlers with little discrimination amongst CCA species (i.e. *P. daedalea* and *C. furcata*), while others were highly selective, responding to only 1 CCA species (i.e. *C. aspera* and *G. favulus*) (Table 3 of main article). Within-genus settlement variability was found in *Platygyra*, with *P. sinensis*

being more selective than *P. daedalea*. There were however, CCA species that were highly inductive within the genus *Platygyra*, with *A.* cf. *boscensei*, *L.* cf. *kotschyanum* and *T.* cf. *tessellatum* inducing >80% mean settlement across the two *Platygyra* spp. (Table 3 of main article).

Overall, *T.* cf. *tessellatum* was the best performing CCA (74.6%) which showed similar levels of mean settlement to the coral rubble treatment (73.3%; Table 3 of main article). *T.* cf. *tessellatum* induced >75% settlement in all the merulinid species tested, except for *C. aspera* (53.3%). Of note *L.* cf. *insipidum* induced the highest settlement in *C. aspera*, with the only other CCA inducing settlement of >70% for this coral species being *N.* cf. *fosliei*. *Porolithon* spp. and *L.* cf. *pygmaeum*, which were moderate–good inducers for acroporid species (66.9–76.1%), did not perform well for the Merulinidae, with mean settlement ranging from 25.2–53.1% (Table 3 of main article). *Amphiroa* cf. *foliacea* induced the lowest mean settlement (7.7%) across the Merulinidae.

Larvae did not show a strong preference for settlement onto live surfaces of CCA treatment chips, with 5 out of the 7 merulinid species settling in low numbers (mean <7%) on 1–5 CCA species (Table S1). *M. elephantotus* and *P. daedalea* never settled onto living surfaces of CCA, despite the latter species displaying high settlement in most treatments (Table S2).

CCA inductive properties within Lobophyllidae

There was a significant effect of CCA treatment on settlement across the two lobophyllid species tested (Kruskal-Wallis: H = 211.503, df = 17, $p<0.001$), with no settlement in the blank treatment detected (Table S3, Figure S40D). Dunn's pairwise tests showed that settlement was comparable to the coral rubble treatment across most CCA species except for *P*. cf. *onkodes* "Chalky" and *A.* cf. *foliacea,* which induced significantly lower settlement ($p<0.05$; Supplementary Material 2). Settlement to *L.* cf. *pygmaeum*, *P*. cf. *onkodes* "Chalky" and *A.* cf. *foliacea* were similar to that found in the sterile aragonite treatment ($p>0.05$).

There was a strong settlement response to the CCA *Sporolithon* sp. by both coral species, with mean settlement of 86.9% (Table S3, Figure S40D), while *Ramicrusta* sp. induced higher settlement in *E. aspera* (85.2%) when compared to *L. corymbosa* (66.5%); this was similarly true for *T.* cf. *tessellatum* with 80.6% settlement for *E. aspera* but only 62.5% for *L. corymbosa,* suggesting *L. corymbosa* is more selective. This is further reflected in the higher settlement levels of *E. aspera* to most CCAs tested in the study (Table 3 of main article). The CCA *M.* cf. *madagascariensis* was similarly inductive (~75%) for both species (Table 3 of main article). Out of all CCA species, *A.* cf. *foliacea* induced the lowest settlement (5.4%; Table 3 of main article).

Lobophyllid larvae did not settle onto the living surface of most CCAs; this only occurred in 2 and 4 treatments for *L. corymbosa* and *E. aspera*, respectively (Table S2). Live-surface settlement was typically low (<5%), however ~16% settlement to the living surface of *L.* cf. *pygmaeum* was seen in *E. aspera*.

CCA inductive properties in Porites lobata

CCA treatment had a significant effect on larval settlement in *P. lobata*. The coral rubble treatment induced 86.6% settlement while the highest CCA settlement was in the *Ramicrusta* sp. treatment (92.5%), followed by *L.* cf. *insipidum* (91.9%) and *T.* cf. *tessellatum* (90.4%; Kruskal-Wallis: H = 101.751, df = 17. $p < 0.001$; Table 3 of main article). There was no settlement of *P. lobata* larvae in the blank treatment and very low settlement (0.9%) found in the aragonite treatment (Table S3,

Figure S40E). Four other CCAs induced high larval settlement (>75%) in *P. lobata* that included *N.* cf. *fosliei* (85.5%), *L.* cf. *kotschyanum* (85.4%), *A.* cf. *boscensei* (78.8%) and *P.* cf. *onkodes* “Orange” (77.6%; Table S3, Figure S40E). The lowest settlement was found in the *L.* cf. *proliferum* treatment (40.3%). Pairwise test showed no difference in settlement to any of the CCAs when compared to the coral rubble treatment (Dunn’s test; $p > 0.05$, Supplementary Material 2). *P. lobata* settled onto the living surfaces of 12 of the CCA treatment chips, with highest settlement found in *T.* cf. *tessellatum* (46.2%), followed by coral rubble (19.4%) and *P.* cf. *onkodes* “Yellow conceptacle” (14.6%).

CCA inductive properties in Fungia fungites

There was a significant effect of CCA treatment on *F. fungites* settlement (Kruskal-Wallis: $H = 58.958$, $df = 17$. $p < 0.001$), although settlement was in general low across all treatments tested and never exceeded 32% (Table 3 of main article, Table S3, Figure S40F). The highest settlement was observed in the *Sporolithon* sp. treatment (32%) followed by *M.* cf. *madagascariensis* (25%) and *Ramicrusta* sp. (23.7%), while coral rubble induced 13.7% settlement. Pairwise tests showed similar levels of settlement across all CCA treatments when compared to the coral rubble treatment, with only *M.* cf. *madagascariensis* and *Sporolithon* sp. inducing significantly higher settlement than the aragonite control (Dunn’s test; $p < 0.05$, Supplementary Material 2). *F. fungites* larvae did not settle to the living surface of CCA (Table S2).

Table S2: Algal treatments, showing species and taxonomic families, ordered by mean settlement (% across all assays; SE in parentheses) across 1) all coral species tested (n_{assay} = 165 per CCA species), 2) coral species in the family Acroporidae only (n_{assay} = 48 per CCA species), 3) coral species in the family Merulinidae only (n_{assay} = 69 per CCA species), 4) corals species in the family Lobophyllidae only (n_{assay} = 24 per CCA species), 5) coral species in the family Poritidae only (n_{assay} = 12 per CCA species), and 6) coral species in the family Fungiidae only (n_{assay} = 12 per CCA species). ***Abbreviations*** – Bla – blank (negative control), Arg – aragonite chip (substrate control), Rub – coral rubble, Pey - *Ramicrusta* sp., Lpro – *Lithothamnion proliferum*, Mmad – *Melyvonnea* cf. *madagascariensis*, Abos – *Adeylithon bosencei*, Hrei – *Hydrolithon reinboldii*, Afol – *Amphiroa foliacea*, Lins – *Lithophyllum insipidum*, Lkot – *Lithophyllum kotchyanum*, Lpyg – *Lithophyllum pygmaeum*, Ttes – *Titanoderma tessellatum*, Nfos – *Neogoniolithon fosliei*, Ponk_O – *Porolithon* cf. *onkodes* "Orange", Ponk_C – *Porolithon* cf. *onkodes* "Chalky", P_yc – *Porolithon* sp. "Yellow conceptacles" and Spo – *Sporolithon* sp. Dunn's pairwise tests were performed to evaluate differences in total settlement to CCA species pairs for all coral species and at the coral family level (Figure S41).

All species ($n_{species}$ = 15)		
**CCA species**	**CCA families**	**Mean settlement (%)**
Ttes	Lithophyllaceae	76.5 (2.3)
Lkot	Lithophyllaceae	70.9 (2.4)
Lins	Lithophyllaceae	68.8 (2.4)
Alg_t	NA	64.8 (2.5)
Pey	Peyssonneliaceae	62.9 (2.6)
Mmad	Mesophyllumaceae	60.6 (2.5)
Abos	Hydrolithaceae	60.5 (2.6)
P_yc	Porolithaceae	58.3 (2.6)
Hrei	Hydrolithaceae	58.2 (2.6)
Spo	Sporolithaceae	56.7 (2.8)
Nfos	Spongitaceae	54.9 (2.7)
Ponk_O	Porolithaceae	51.0 (2.9)
Lpro	Hapalidiaceae	45.2 (2.8)
Lpyg	Lithophyllaceae	40.8 (2.8)
Ponk_C	Porolithaceae	40.5 (3.2)
Afol	Lithophyllaceae	14.5 (2.0)
Arg	NA	4.9 (1.1)
Bla	NA	2.9 (0.9)

Acroporidae ($n_{species}$ = 4)		
**CCA species**	**CCA families**	**Mean settlement (%)**
Lins	Lithophyllaceae	85.7 (2.2)
Ttes	Lithophyllaceae	81.3 (3.5)
Lkot	Lithophyllaceae	80.2 (3.0)
P_yc	Porolithaceae	72.4 (3.6)
Ponk_C	Porolithaceae	63.2 (5.1)
Ponk_O	Porolithaceae	63.0 (4.4)
Lpyg	Lithophyllaceae	61.8 (5.3)
Hrei	Hydrolithaceae	61.0 (4.4)
Alg_t	NA	58.7 (5.0)
Abos	Hydrolithaceae	58.4 (4.7)
Nfos	Spongitaceae	53.7 (4.4)
Pey	Peyssonneliaceae	51.8 (4.2)
Mmad	Mesophyllumaceae	48.3 (4.3)
Spo	Sporolithaceae	37.0 (4.4)
Lpro	Hapalidiaceae	33.4 (4.5)
Afol	Lithophyllaceae	17.4 (3.5)
Arg	NA	15.3 (3.2)
Bla	NA	10.0 (2.9)

Merulinidae ($n_{species}$ = 7)		
**CCA species**	**CCA families**	**Mean settlement (%)**
Ttes	Lithophyllaceae	83.4 (2.4)
Lkot	Lithophyllaceae	75.5 (3.2)
Alg_t	NA	75.4 (3.1)
Mmad	Mesophyllumaceae	73.1 (3.7)
Abos	Hydrolithaceae	68.7 (3.9)
Pey	Peyssonneliaceae	67.7 (4.0)
Lins	Lithophyllaceae	66.4 (3.2)
Spo	Sporolithaceae	64.0 (4.4)
Hrei	Hydrolithaceae	63.2 (4.2)
Nfos	Spongitaceae	62.1 (3.9)
P_yc	Porolithaceae	61.4 (3.9)
Lpro	Hapalidiaceae	55.3 (4.7)
Ponk_O	Porolithaceae	45.8 (4.7)
Ponk_C	Porolithaceae	33.2 (5.0)
Lpyg	Lithophyllaceae	32.4 (4.5)
Afol	Lithophyllaceae	11.1 (2.9)
Arg	NA	0.6 (0.4)
Bla	NA	0.0

Lobophyllidae ($n_{species}$ = 2)		
**CCA species**	**CCA families**	**Mean settlement (%)**
Spo	Sporolithaceae	86.9 (3.8)
Pey	Peyssonneliaceae	75.8 (4.2)
Mmad	Mesophyllumaceae	73.5 (4.4)
Ttes	Lithophyllaceae	71.6 (6.3)
Lins	Lithophyllaceae	63.9 (5.4)
Lkot	Lithophyllaceae	61.6 (6.5)
Alg_t	NA	61.3 (5.3)
Hrei	Hydrolithaceae	57.9 (4.9)
Abos	Hydrolithaceae	57.5 (6.1)
Lpro	Hapalidiaceae	55.9 (6.4)
Ponk_O	Porolithaceae	50.8 (7.6)
P_yc	Porolithaceae	45.4 (5.7)
Nfos	Spongitaceae	44.1 (8.0)
Lpyg	Lithophyllaceae	32.3 (5.7)
Ponk_C	Porolithaceae	19.6 (6.8)
Afol	Lithophyllaceae	5.4 (2.3)
Arg	NA	0.4 (0.4)
Bla	NA	0.0

Poritidae ($n_{species}$ = 1)		
**CCA species**	**CCA families**	**Mean settlement (%)**
Pey	Peyssonneliaceae	92.5 (4.5)
Lins	Lithophyllaceae	91.9 (3.3)
Ttes	Lithophyllaceae	90.4 (2.4)
Alg_t	NA	86.6 (6.3)
Nfos	Spongitaceae	85.5 (3.8)
Lkot	Lithophyllaceae	85.4 (5.9)
Abos	Hydrolithaceae	78.8 (5.1)
Ponk_O	Porolithaceae	77.6 (6.5)
Hrei	Hydrolithaceae	74.8 (6.3)
Ponk_C	Porolithaceae	69.5 (11.5)
P_yc	Porolithaceae	65.1 (8.2)
Lpyg	Lithophyllaceae	60.4 (10.5)
Spo	Sporolithaceae	58.4 (7.5)
Afol	Lithophyllaceae	53.4 (9.3)
Mmad	Mesophyllumaceae	49.0 (7.0)
Lpro	Hapalidiaceae	40.3 (12.3)
Arg	NA	0.8 (0.8)
Bla	NA	0.0

Fungiidae ($n_{species}$ = 1)		
**CCA species**	**CCA families**	**Mean settlement (%)**
Spo	Sporolithaceae	31.6 (9.1)
Mmad	Mesophyllumaceae	24.4 (6.0)
Pey	Peyssonneliaceae	23.7 (9.1)
Lpro	Hapalidiaceae	17.3 (5.3)
Ttes	Lithophyllaceae	13.8 (7.0)
Alg_t	NA	13.7 (5.2)
Lkot	Lithophyllaceae	11.2 (3.5)
Nfos	Spongitaceae	10.1 (6.3)
Abos	Hydrolithaceae	9.8 (5.7)
Ponk_O	Porolithaceae	6.1 (2.6)
Ponk_C	Porolithaceae	4.8 (3.7)
P_yc	Porolithaceae	3.1 (3.1)
Lpyg	Lithophyllaceae	2.5 (2.5)
Hrei	Hydrolithaceae	1.8 (1.2)
Lins	Lithophyllaceae	1.8 (1.2)
Bla	NA	0.0
Arg	NA	0.0
Afol	Lithophyllaceae	0.0

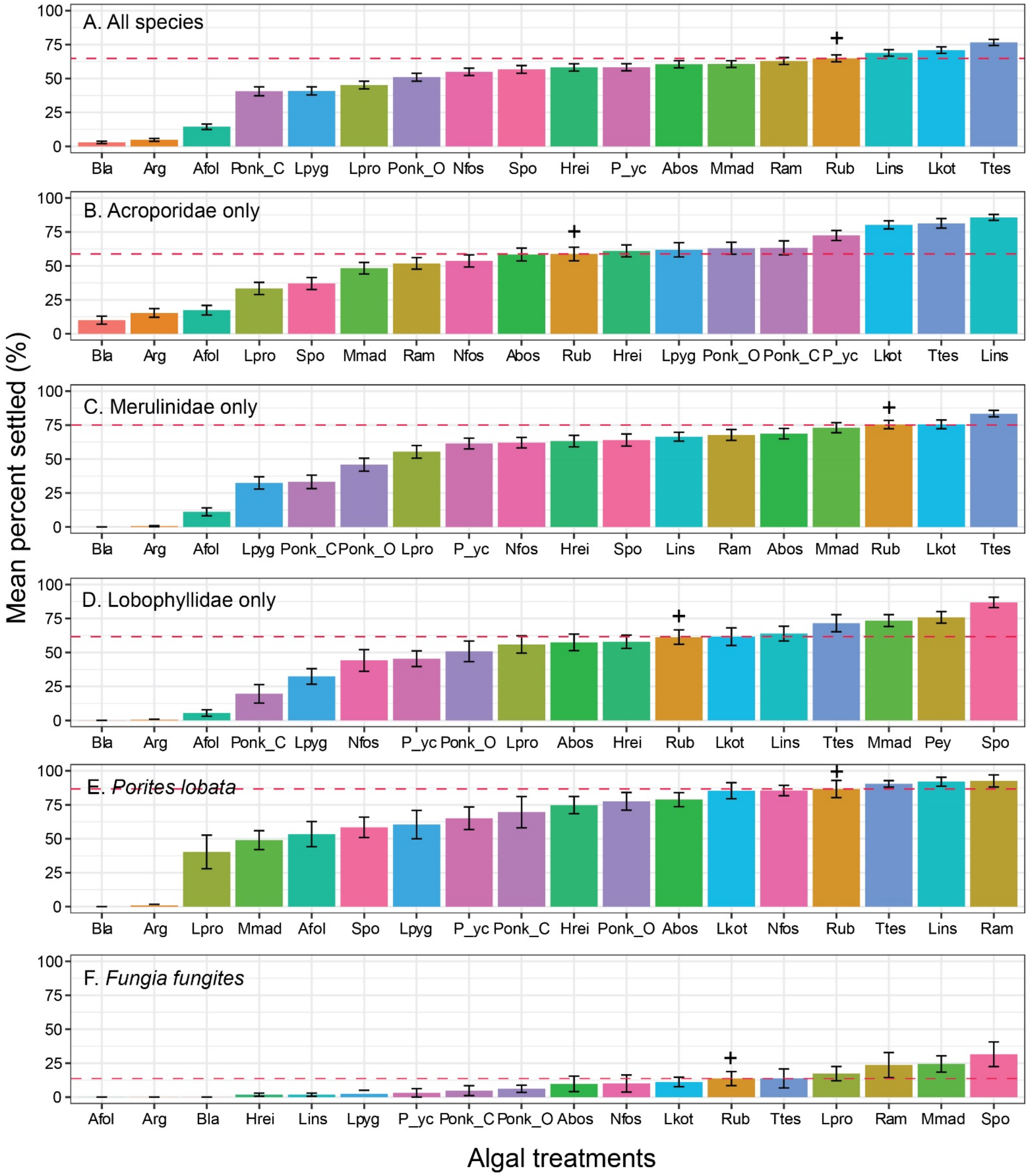

Figure S40: Mean settlement (±SE) for A) all species combined ($n_{species}$ = 15, n_{assay} = 165 per CCA species), B) Acroporidae species ($n_{species}$ = 4, n_{assay} = 48 per CCA species), C) Merulinidae species ($n_{species}$ = 7, n_{assay} = 69 per CCA species), D) Lobophyllidae species ($n_{species}$ = 2, n_{assay} = 24 per CCA species), E) *Porites lobata* (n_{assay} = 12 per CCA species) and F) *Fungia fungites* (n_{assay} = 12 per CCA species). Responses to algal treatments are ordered in ascending order from left to right based on mean percent settled. Colours correspond to algal treatments and algal abbreviations are as defined in Table 2 of main article. Horizontal red dashed lines represent settlement level in the coral rubble treatment (see cross hairs) for each coral family plots. Significant Dunn's pairwise tests plots are provided in Figure S41.

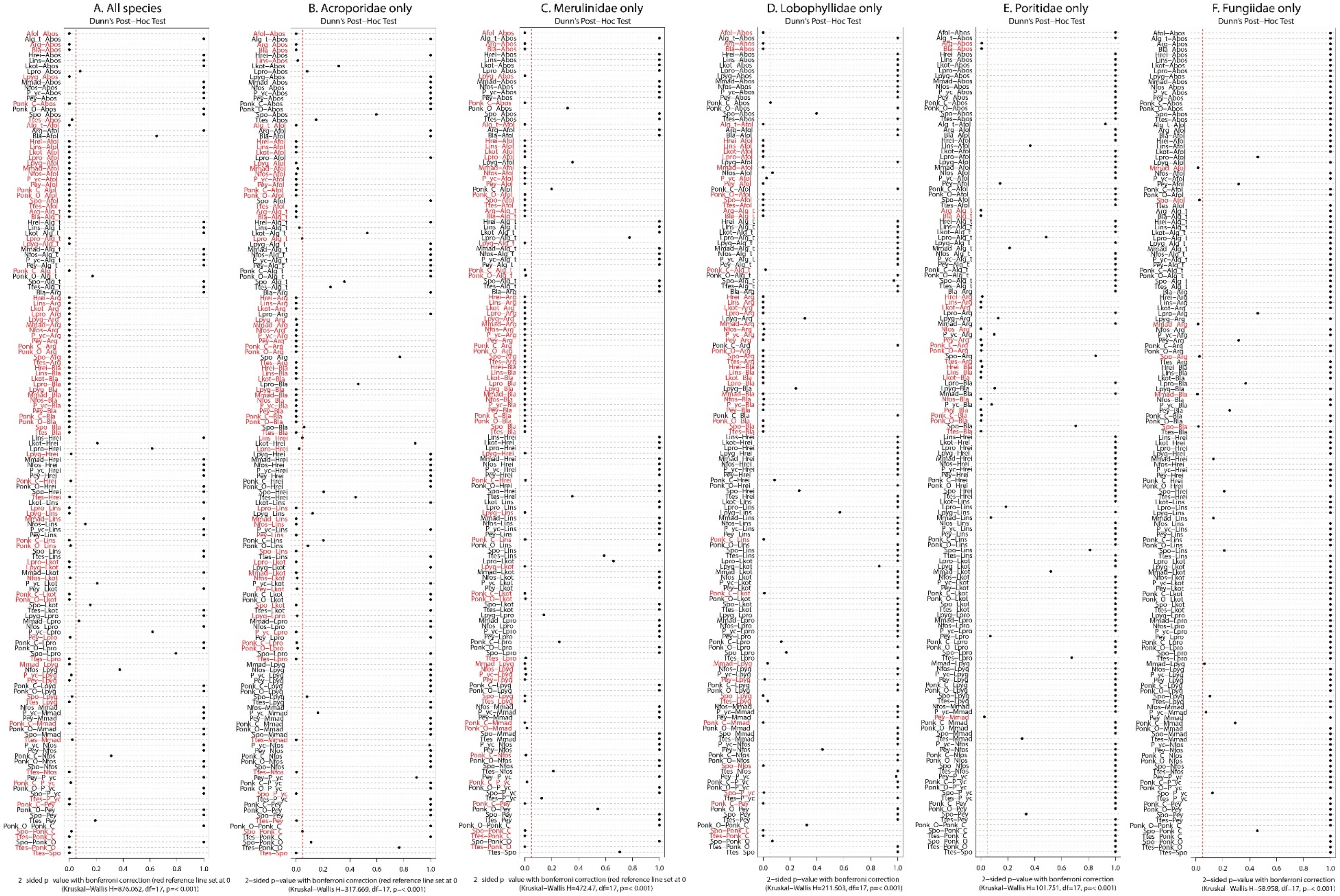

Figure S41: Dunn's pairwise comparisons for A) all species combined ($n_{species}$ = 15, n_{assay} = 165 per CCA species), B) Acroporidae species only ($n_{species}$ = 4, n_{assay} = 48 per CCA species), C) Merulinidae species only ($n_{species}$ = 7, n_{assay} = 69 per CCA species), D) Lobophyllidae species only ($n_{species}$ = 2, n_{assay} = 24 per CCA species), E) Poritidae species only ($n_{species}$ = 1, n_{assay} = 12 per CCA species) and F) Fungiidae species only ($n_{species}$ = 1, n_{assay} = 12 per CCA species).. Red-dashed lines in Dunn's pairwise tests represent p = 0.05. Kruskal=Wallis test statistics are provided below Dunn's pairwise plots. Alg_t refers to the coral rubble treatment and Pey refers to the *Ramicrusta* sp. treatment.

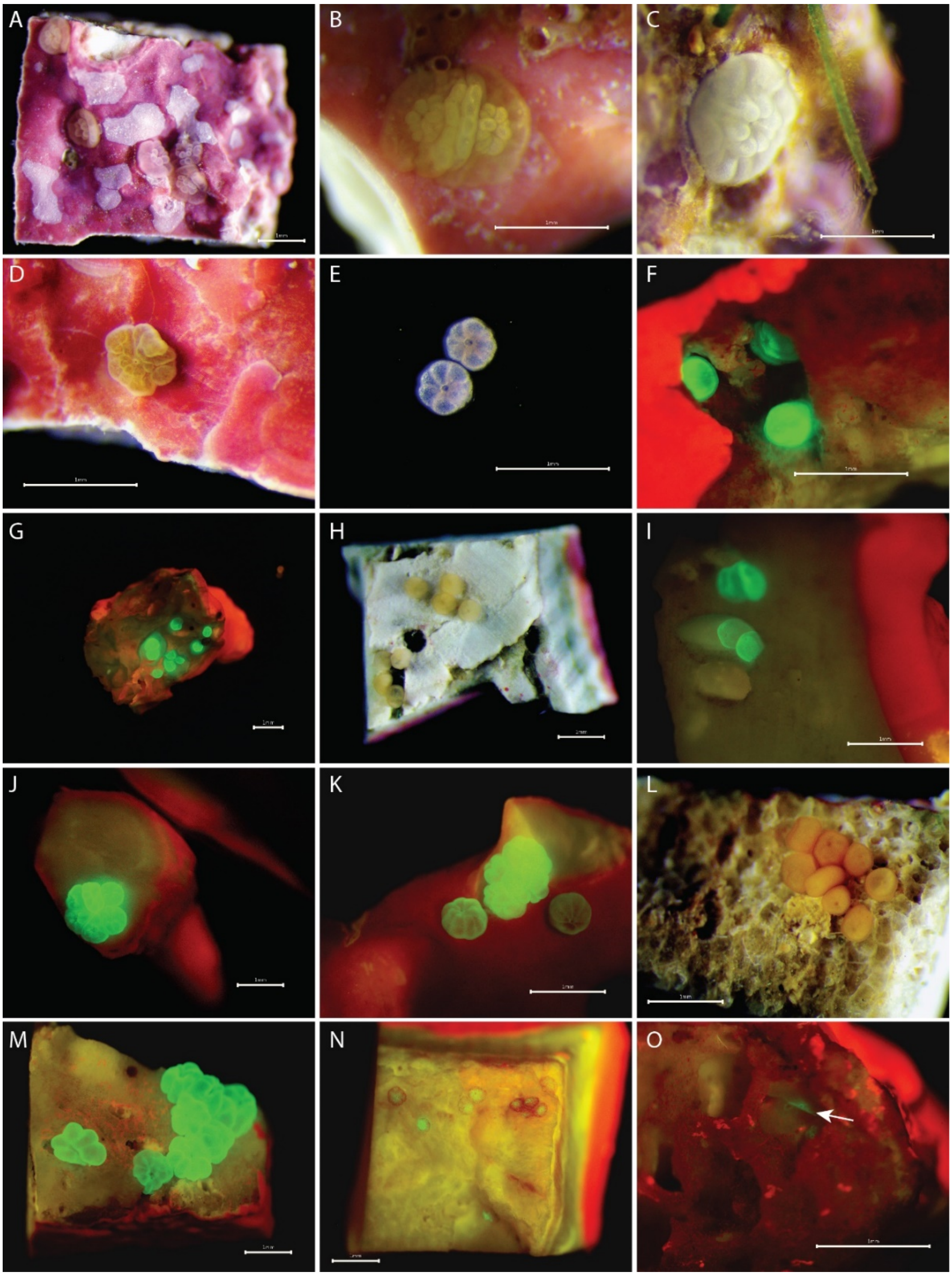

Figure S42: Successful settlement of larval species tested in settlement assays in this study when presented with live chips of algal treatments. A) *A. tenuis* on living tissue of *H.* cf. *reinboldii*, B) *A. anthocercis* on living tissue of *L.* cf. *pygmaeum*, C) *A. hyacinthus* on living tissue of *L.* cf. *insipidum*, D) *M. aequituberculata* on living tissue of *T.* cf. *tessellatum*, E) *C. aspera* on the bottom of well underneath *L.* cf. *proliferum*, F) *C. furcata* in the matrix of *M.* cf. *madagascariensis*, G) *D. favus* on living tissue of *A.* cf. *foliacea*, H) *G. favulus* on the underside of *P.* cf. *onkodes* "Orange", I) *M. elephantotus* in the matrix of *L.* cf. *proliferum*, J) *P. sinensis* on the side of *L.* cf. *pygmaeum*, K) *P. daedalea* on the living tissue of *L.* cf. *pygmaeum*, L) *E. aspera* on the underside of *H.* cf. *reinboldii*, M) *L. corymbosa* on the underside of *M.* cf. *madagascariensis*, N) *P. lobata* on the underside of *Sporolithon* sp., and O) *F. fungites* in the matrix of *M.* cf. *madagascariensis* (white arrow). Excitation of green autofluorescence of some larval species was used to aid the detection of settlers in assays. Scale bars = 1 mm.
